# Statins Diversity Revealed by the Deep-Sea-Derived Fungus *Penicillium viridicatum*

**DOI:** 10.3390/md23020087

**Published:** 2025-02-17

**Authors:** Meng Zhang, Rong Chao, Jia-Jian Wang, Zi-Han Xu, Ji-Hong Zhang, Da-Li Meng, Tai-Zong Wu, Xian-Wen Yang

**Affiliations:** 1 Hainan Academy of Medical Sciences, Hainan Medical University, 3 Xueyuan Road, Haikou 571199, China; 13889240729@163.com (M.Z.); xuzihan@njust.edu.cn (Z.-H.X.); 2 Key Laboratory of Marine Genetic Resources, Third Institute of Oceanography, Ministry of Natural Sources, 184 Daxue Road, Xiamen 361005, China; chaorong@tio.org.cn; 3 School of Traditional Chinese Materia Medica, Shenyang Pharmaceutical University, Wenhua Road 103, Shenyang 110016, China; mengdl@163.com; 4 Laboratory of Molecular Pharmacology, Medical School, Kunming University of Science and Technology, Kunming 650500, China; jiawang2025@163.com (J.-J.W.); zhjihong2000@kust.edu.cn (J.-H.Z.)

**Keywords:** deep-sea-derived fungus, statins, *Penicillium viridicatum*, mutant p53, cancer

## Abstract

Seven new (**1**–**7**) and six known (**8**–**13**) statin derivatives were obtained from the deep-sea-derived fungus *Penicillium viridicatum* MCCC 3A00265. The structures assigned to the new compounds were based on a comprehensive analysis of the spectroscopic data, with absolute configurations established by Mosher analysis and biogenetic consideration. Most of the new compounds (**1**–**5** and **7**) share an octohydronaphthalene backbone, except that viridecalin F (**6**) possesses an uncommon naphthalene core. Viridecalins C (**3**) and F (**6**) and the two known compounds **9** and **11** exhibit considerable ability in reactivating mutant p53 protein at 10 μM, while viridecalin C showcases the most potent reactivation activity, indicating the potential of application in cancer therapy.

## 1. Introduction

**Figure 1:**
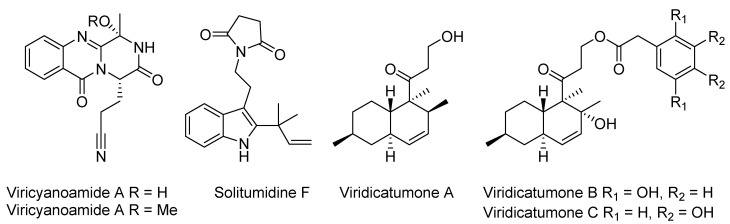
Compounds isolated from the deep-sea-derived *Penicillium viridicatum* in the literature.

Statins are well-known agents for the treatment of hyperlipidemia, both as monotherapy and in combination therapy, representing a successful example of natural product-derived drug discovery, more specifically, from filamentous fungi [1]. Natural statins share a common hexahydro-naphthalene core and a 3,5-dihydroxy-hepta(e)noic acid side chain, which mimic the 3-hydroxy-3-methylglutaryl-coenzyme A(HMG-CoA), and act by occupying a portion of the binding site of the enzyme, thereby blocking access of this substrate (HMG-CoA) to the active site [2]. The modifications in the core structure, including substitution and dehydrogenation at the hydro-naphthalene ring and oxidation at the hydroxycarboxylate side chain, were thought to contribute to their bioactivity, such as half-life elimination and bioavailability [3]. Beyond their lipid-lowering effects, statins and their derivatives have demonstrated a wide range of additional pharmacological activities, including anti-inflammatory, antioxidant, and anticancer properties, which spurred interest in exploring structurally diverse statin-like compounds from novel sources [4,5]. Marine microorganisms, especially deep-sea-derived (>1000 m) fungi, are an enormous reservoir of structurally new and bioactive metabolites due to their extremely different environment compared to their terrestrial counterparts [6,7]. As an example, a previous chemical investigation of the deep-sea-derived *Penicillium viridicatum* resulted in the discovery of (i) viricyanoamides A and B, the sole representatives of quinazolinones that feature a nitrile group and exhibit significant mutant p53 reactivating activity; (ii) solitumidine F, an indole terpenoid with a rare pyrrolidinedione unit incorporated; (iii) and the herbicidal decline polyketides viridicatumones A–C [8,9] (Figure 1).

**Figure 2:**
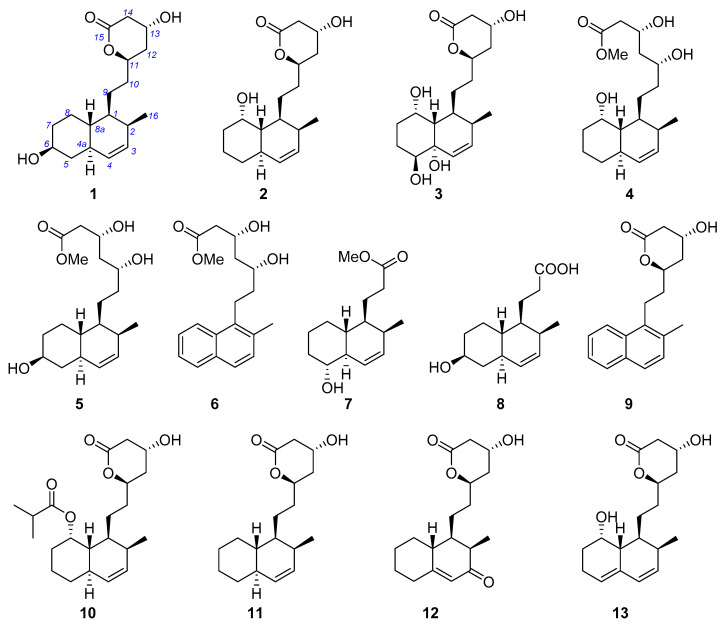
Compounds **1**–**13** isolated from the deep-sea-derived *Penicillium viridicatum* MCCC 3A00265.

In a search of intriguing natural products from marine fungi, we identified an Atlantic abyssal sediment-derived *Penicillium viridicatum* as a statin derivative producer. A systematic investigation into this strain led to the discovery of seven new (**1**–**7**) and six known (**8**–**13**) decalin-type polyketides (Figure 2). Herein is an account of the isolation, structure elucidation, and bioactivities of the isolates.

## 2. Results and Discussion

The EtOAc extract of a scaled-up rice cultivation of *Penicillium viridicatum* MCCC 3A00265 was subjected to sequential solvent partitioning, followed by isolation and purification by column chromatography (CC) over silica gel, ODS, Sephadex LH-20, and by preparative thin-layer chromatography (PTLC) to yield 13 compounds (**1**–**13**). By comparison of the NMR, MS, and OR data with those reported in the references, six known compounds were identified, eujavanoic acid B (**8**) [10], solistatin (**9**) [11], dihydrocompactin (**10**) [10,12,13], desmethyl-dihydromonacolin L (**11**) [14], 3,5-dihydro-3-oxo-ML-236C (**12**) [10], and 6-demethylmonacolin J (**13**) [15], while seven new ones (**1**–**7**) were elucidated by detailed spectroscopic analysis and biogenetic considerations as summarized below.

**Figure 3:**
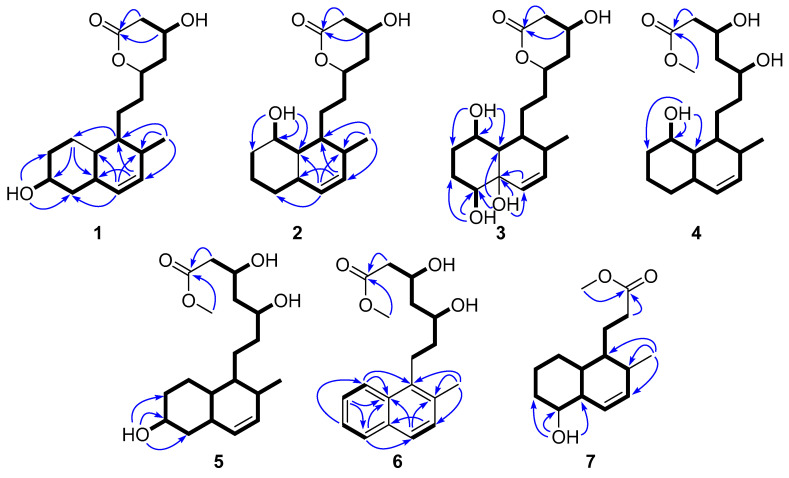
^1^H-^1^H COSY (bold line) and HMBC (blue arrow) correlations of **1**–**7**.

**Figure 4:**
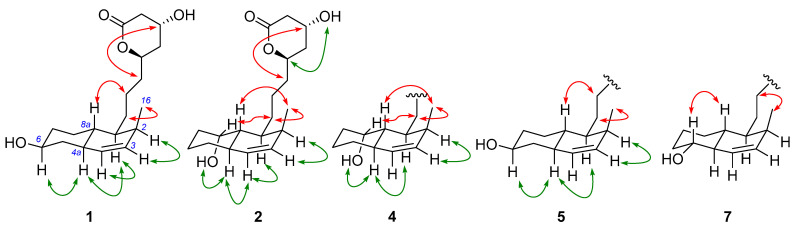
NOESY correlations of **1**–**2**, **4**–**5**, and **7** (red: correlations among β-oriented protons; green: correlations among α-oriented protons).

**Figure 5:**
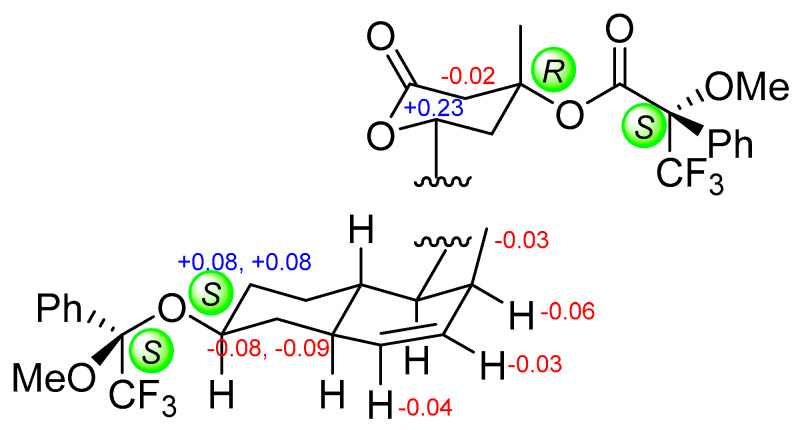
Mosher’s analysis of **1**, with the Δ*_S_*_−*R*_ value highlighted in blue (positive) and red (negative).

The positive HRESIMS analysis of **1** revealed the molecular formula C_18_H_28_O_4_, requiring five degrees of unsaturation. The ^1^H and ^13^C NMR spectroscopic data of **1** (Table 1, Figures S1 and S2) showcased resonances for one methyl doublet, seven *sp*^3^ methylene, seven *sp*^3^ methine, two *sp*^2^ methine, and one carbonyl carbon, accounting for five degrees of unsaturation. Therefore, **1** incorporated three rings. A suite of ^1^H-^1^H COSY correlations and diagnostic HMBC cross-peaks observed from H_3_-16 to C-1/2/3, H-1 to C-8, H_2_-8 to C-6/4a, 6-OH to C-5/6/7, as well as H-4 to C-2/5/8a allowed for the connection of a 2-methyl-6-hydroxy-Δ^3^-octohydronaphthalene segment and a side chain terminated with an ester unit supported by HMBC signals of H-13/H_2_-14 to the carbonyl carbon C-15 (Figure 3). Considering the deshielding of the oxygenated methine C-11 and the remaining one unsaturation, the lactone ring was inferred through a (C-11)-O-(C-15) linkage. In regards to the stereochemistry of **1**, the *trans* configuration of the dihydrodecalin was indicated by the NOESY correlations observed from H-1 to H-5 and H-8a to H_2_-10 (Figure 4). Additionally, the correlations of H-4a/H-6, H-4a/H-1 indicated those protons were on the same side, while H_3_-16/H_2_-9 suggested they were toward the opposite side of the former (Figure 4). The relative configuration of the lactone ring was implied by the NOESY signal between H_2_-10 and H-13. Due to a convergent biosynthetic pathway of the *trans*-octohydronaphthalene and the hydroxycarboxylate side chain shared in this type of known statin derivative [3], the absolute configuration of C-4a/8a, C-11/13 was deduced to be the same as that of the cometabolites dihydrocompactin (**10**) and demethyl-dihydromonacolin L (**11**), for which the configurations of rest chiral centers could be indicated accordingly. To confirm this assignment, a modified Mosher’s analysis was performed. The deshielding of H_2_-7 and H-11 (Δ*_S_*_-*R*_ > 0) and the shielding of H-2, H-3, H-4, H_2_-5, and H_2_-14 (Δ*_S_*_-*R*_ < 0) in the *S*-MTPA ester compared to the *R*-MTPA derivative unambiguously established the absolute configurations of C-6 and C-13 as 7*S*, 13*R* (Figure 5), thereby inferring the configurations of C-1/2/4a/8a/11. Therefore, the complete structure of **1** was established and named viridecalin A.

The positive HRESIMS spectrum of **2** returned an identical molecular formula, C_18_H_28_O_4_, as that of **1**. A close comparison of their NMR data revealed a high degree of similarities, with principal differences on the carbon chemical shifts of the octohydronaphthalene part. A scrutiny of the ^1^H-^1^H COSY and HMBC spectra of **2** indicated that the hydroxy group was substituted at C-8, which was evidenced by the COSY correlations of 8-OH/H-8/H-1/H-16 as well as HMBC cross-peaks from 8-OH to C-7/8/8a (Figure 3). The relative configuration of 8-OH was determined by the NOESY spectrum. The signals among H-8/H_2_-9, H-8a/H_2_-9/H_3_-16 suggested these protons were on the same side of the plane, while 8-OH/H-4a/H-1 were on the opposite side, evident by correlations of 8-OH/H-4a, H-4a/H-4/H-1 (Figure 4). The absolute configuration of **2** was deduced to be the same as **1** and the known cometabolites based on a biogenetic consideration. Therefore, the structure of **2** was assigned and named viridecalin B.

**Table 1:** ^1^H (400 Hz) and ^13^C (100 Hz) NMR spectroscopic data of **1**–**3** in DMSO-*d*_6_.

Pos.	1		2		3	
	^1^H *^a^*	^13^C	^1^H *^a^*	^13^C	^1^H *^a^*	^13^C
1	1.38, m	40.5 d	1.60, m	37.5 d	2.06, tt (11.8, 3.5)	32.2 d
2	2.21, m	31.5 d	2.20, m	31.5 d	2.23, qd (7.0, 5.0)	31.8 d
3	5.57, ddd (9.8, 4.6, 2.6)	132.6 d	5.52, m	132.4 d	5.68, dd (9.7, 5.0)	136.7 d
4	5.31, d (9.8)	130.5 d	5.32, d (9.8)	132.0 d	5.30, d (9.7)	131.6 d
4a	1.65, m	41.3 d	2.15, m	35.5 d		73.7 s
5	0.88, m; 1.82, m	42.0 t	0.90, dd (12.7, 2.8); 1.64, m	33.4 t	3.48, br s	72.2 d
6	3.41, m	68.8 d	1.39, m; 1.65, m	20.6 t	1.34, d (13.9) 2.03, tt (13.9, 3.3)	23.2 t
7	1.09, m; 1.90, m	35.9 t	1.35, m; 1.72, m	34.4 t	1.54, d (13.9); 1.71, m	28.5 t
8	0.91, m; 1.76, m	27.1 t	3.93, br s	64.1 d	3.89 *^b^*	66.9 d
8a	0.92, m	38.3 d	0.98, dd (10.8, 10.8)	42.9 d	1.58, dd (12.0, 2.1)	36.1 d
9	1.18, m; 1.54, tt (12.2, 3.8)	23.6 t	1.16, m; 1.71, m	23.1 t	1.19, m; 1.68, m	23.4 t
10	1.40, m; 1.63, m	32.5 t	1.40, m; 1.68, m	33.0 t	1.44, m; 1.66, m	33.1 t
11	4.54, tt (11.0, 3.8)	75.6 d	4.54, m	76.6 d	4.55, m	77.1 d
12	1.66, m; 1.80, m	35.2 t	1.68, m; 1.81, m	35.8 t	1.68, m; 1.82, d (13.9)	35.5 t
13	4.09, m	61.3 d	4.10, m	61.8 d	4.11 *^b^*	62.0 d
14	2.36, dd (3.3, 1.6) 2.62, dd (3.3, 1.6)	38.6 t	2.38, d (17.2) 2.62, dd (17.2, 4.4)	39.0 t	2.36 d (17.5) 2.61 dd (17.5, 4.6)	38.9 t
15		170.4 s		170.9 s		172.0 s
16	0.79, d (7.0)	14.6 q	0.78, d (7.0)	15.1 q	0.73, d (7.0)	13.1 q
4a-OH					5.53, s	
5-OH					4.77, d (4.2)	
6-OH	4.52, d (4.5)					
8-OH			4.13 *^b^*		5.05, d (7.1)	
13-OH	5.15, d (3.0)		5.17, d (2.9)		5.47, d (2.8)	

*^a^ δ* in ppm, *J* in Hz within parentheses. *^b^* Resonance obscured by solvent peak.

Compound **3** was assigned the molecular formula C_18_H_28_O_6_, accounting for five degrees of unsaturation. Comparison of the ^1^H and ^13^C NMR spectroscopic data of **3** with **2** revealed great similarity, except for the major difference on the octohydronaphthalene unit: two additional hydroxy groups showing up at the C-4a and C-5 positions in **3**. The assumption was supported by the COSY correlation of 6-OH/H-6 as well as HMBC correlations from 4a-OH to C-4/4a/5/8a, from H-3 to C-4a/5, and from 5-OH to C-4a/5/6 (Figure 3). On the basis of the small coupling constant of ^3^*J*_H-8/H-8a_ (2.1 Hz) and the broad singlet status of H-5, 5-OH and 8-OH were assigned as axial oriented. Due to a biogenetic ground, the absolute configurations of C-4a/8a and C-11/13 were deduced to be the same as those of **1** and **2**. Thus, the structure of **3** was assigned and named viridecalin C.

The molecular formula of **4** was identified as C_19_H_32_O_5_ based on an analysis of its positive HRESIMS spectrum, requiring four degrees of unsaturation. The ^1^H and ^13^C NMR spectroscopic data of **4** (Table 2) revealed very similar resonances as those of **2**, except for the presence of an additional methoxy group as well as the shielding effect of H-11 (Δ*δ*_H_ − 1.0) and C-11 (Δ*δ*_H_ − 8.1) in **4**. This, along with the observation of a free hydroxy unit at C-11 that was evident from the ^1^H-^1^H COSY correlation of 11-OH/H-11, confirmed the opening of the lactone ring in **4**. A scrutiny of the 2D NMR spectra of **4** established its planar structure as shown in Figure 3. The 8-OH was determined as axial based on the broad singlet peak of H-8 (*δ*_H_ 3.93, br s) and the NOESY correlation among 8-OH, H-4a, and H-1. All the chiral centers were assumed to retain the same absolute configurations as those of **2** based on the convergent biosynthetic consideration. Accordingly, the structure of **4** was determined and was given a trivial name, viridecalin D.

Analysis of the positive HRESIMS spectrum of **5** returned the same isomeric molecular formula as that of **4**. Moreover, the ^1^H and ^13^C NMR spectroscopic data of **5** showed almost the same as those of **4**, except that the hydroxy group was located at the C-6 position in **4** instead of the C-8 position in **5**. This was supported by the COSY correlation of 6-OH/H-6 and HMBC correlations from 6-OH to C-5/6/7 (Figure 3). The NOESY correlation of H-6/H-4a/H-1 suggested they were on the same side (Figure 4). On the basis of the above evidence, the structure of **5** was then assigned and name viridecalin E.

**Table 2:** ^1^H (400 Hz) and ^13^C (100 Hz) NMR spectroscopic data of **4**–**7** in DMSO-*d*_6_.

Pos.	4		5		6		7	
	^1^H *^a^*	^13^C	^1^H *^a^*	^13^C	^1^H *^a^*	^13^C	^1^H *^a^*	^13^C
1	1.56, m	37.1 d	1.35, m	40.8 d		135.7 s	1.40, m	40.3 d
2	2.16, m	31.0 d	2.20, m	31.5 d		132.5 s	2.16, dd (9.0, 6.3)	30.9 d
3	5.51, m	132.1 d	5.57, m	132.9 d	7.32, d (8.4)	129.1 d	5.60, m	132.4 d
4	5.31, d (9.8)	131.4 d	5.31, d (9.8)	130.4 d	7.65, d (8.4)	125.7 d	5.90, d (10.0)	127.0 d
4a	2.14, m	35.0 d	1.65, dd (9.8, 9.0)	41.4 d		132.1 s	1.48, ddd (10.4, 10.4, 1.6)	50.8 d
5	0.88, m; 1.61, m	32.9 t	0.89, m; 1.83, d (12.9)	42.1 t	7.83, d (8.1)	128.4 d	2.96, ddd (14.5, 10.4, 4.8)	74.5 d
6	1.37, m; 1.64, m	20.1 t	3.42 *^b^*	68.9 d	7.42, m	124.5 d	1.14, dd (10.4, 10.4); 1.81, m	35.7 t
7	1.35, m; 1.72, d (13.0)	33.9 t	1.10, m; 1.90, d (12.0)	36.0 t	7.50, m	125.9 d	1.26, m	25.7 t
8	3.93, br s	63.7 d	0.90, m; 1.77, m	27.1 d	8.07, d (8.4)	123.5 d	0.83, dd (12.3, 3.1); 1.69, m	28.0 t
8a	0.94, m	42.6 d	0.90, m	38.4 d		131.6 s	1.03, m	36.6 d
9	1.09, m; 1.64, m	22.8 t	1.13, m; 1.50, m	23.9 t	2.98, td (13.3, 5.3) 3.17, td (9.9, 5.3)	24.0 t	1.26, m; 1.78, m	24.0 t
10	1.19, m; 1.34, m	34.4 t	1.22, m; 1.35, m	34.4 t	1.53, m; 1.65, m	37.3 t	2.20, dd (9.6, 6.5) 2.35, ddd (15.4, 10.0, 5.2)	31.2 t
11	3.54 *^b^*	68.5 d	3.55 *^b^*	68.0 d	3.78, m	67.9 d		173.6 s
12	1.47, m	44.3 t	1.48, m	44.2 t	1.58, m	44.0 t	3.59, s	51.3 q
13	4.00, m	65.8 d	4.00, m	65.8 d	4.05, m	65.6 d	0.77, d (7.1)	14.5 q
14	2.29, dd (15.0, 8.6) 2.45, dd (15.0, 4.4)	42.2 t	2.29, dd (14.5, 8.2) 2.47, dd (14.5, 4.3)	42.3 t	2.34, dd (14.6, 8.6)	42.3 t	1.41, m	
15		171.8 s		170.9 s	2.50 *^b^*	171.8 s		
16	3.57, s	51.1 q	3.57, s	51.1 q	3.57, s	51.1 q		
17	0.76, d (7.0)	14.7 q	0.79, d (7.0)	15.1 q	2.46, s	19.6 q		
5-OH							4.59, d (5.6)	
6-OH			4.52, d (3.8)					
8-OH	4.05, m							
11-OH	4.45, d (4.7)		4.45, d (4.7)		4.79, d (5.0)			
13-OH	4.77, d (4.8)		4.76, d (4.8)		4.85, d (5.0)			

*^a^ δ* in ppm, *J* in Hz within parentheses. *^b^* Resonance obscured by solvent peak.

The positive HRESIMS spectrum of **6** assigned its molecular formula as C_19_H_24_O_4_, requiring eight degrees of unsaturation. Analysis of the ^1^H and ^13^C NMR spectroscopic data of **6** revealed resonances for an ortho-di-substituted naphthalene ring and a similar methoxylated hydroxycarboxylate side chain as **5**, accounting for all the eight degrees of unsaturation. The key HMBC correlations observed from H_3_-16 to C-1/2/3 and H_2_-9 to C-1 determined the planar structure of **6**. The absolute configurations of C-11 and C-13 were deduced to be the same as those of **5** due to the biogenetic ground. Accordingly, the structure of **6** was determined and named viridecalin F.

The positive HRESIMS spectrum of **7** revealed the molecular formula C_15_H_24_O_3_, requiring four degrees of unsaturation. A suite of ^1^H-^1^H COSY correlations together with HMBC cross-peaks from H_3_-16 to C-1/2/3, from 5-OH to C-4a/5/6, and from H_2_-10 to the carbonyl carbon C-11 conformed a similar octohydronaphthalene segment as that of **6** and a C_3_ hydroxycarboxylate chain. The connection of the two parts was implied by the COSY signals of H-1/H_2_-9. Accordingly, the structure of **7** was established and was named viridecalin G.

**Figure 6:**
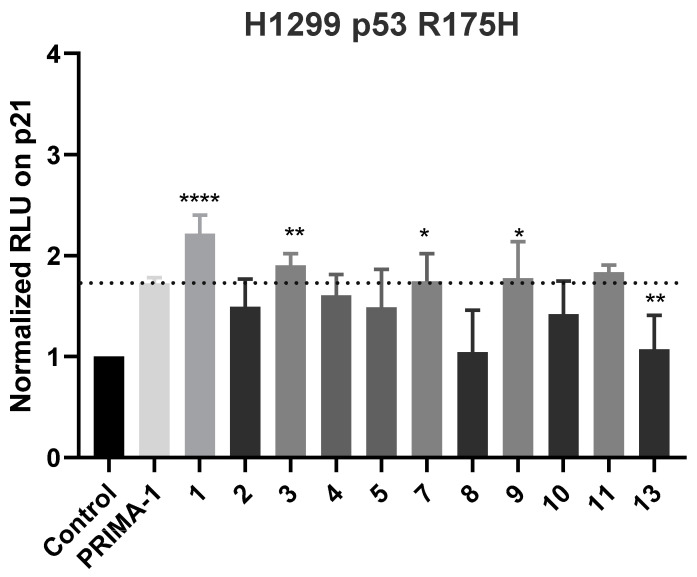
The reactivation screening of mutant p53 activity for the isolates. Results are represented as means ± SD of three independent experiments. * *p* < 0.0132, ** *p* < 0.0293, **** *p* < 0.0001 vs. the control group.

Referred to as the “guardian of the genome,” p53 is a tumor suppressor protein encoded by the TP53 gene in humans and reported mutated in over 50% of tumor cells [16]. These mutations result in the loss of p53’s tumor-suppressing capabilities and contribute to tumor progression. Consequently, activating the p53 signaling pathway in tumor cells represents a promising strategy for cancer therapy [17]. Statins have been identified as degradation inducers for misfolded or conformationally incorrect p53 mutants and can also activate the p53 signaling pathway [18,19]. The antitumor potential of 11 statins (**6** and **12** were not included due to a low quantity) from MCCC 3A00265 was evaluated by analyzing their ability to reactivate mutant p53 using relative fluorescence intensity as we reported previously [20,21]. Of note, compound **1** significantly enhanced the activity of the p53 response element p21 (>2-fold) in p53-deficient H1299 cells at 10 μM, while compounds **3**, **7**, **9**, and **11** exhibited responsivity comparable to the positive control PRIMA-1, a known mutant p53 reactivator (Figure 6). The cytotoxicity of the new compounds **1**–**7** was also evaluated on the adenocarcinomic human alveolar basal epithelial cells A549 using the CCK-8 assay, returning no visible inhibition at the concentration of 20 μM.

## 3. Materials and Methods

### 3.1. General Experimental Procedures

The NMR spectra were recorded in DMSO-*d*_6_ on Bruker 400 MHz or 600 MHz spectrometers (Billerica, Massachusetts, USA). The HRESIMS spectra were measured on a Waters Xevo G2 Q-TOF mass spectrometer (Milford, Massachusetts, USA). Optical rotations were measured with an Anton Paar MCP100 polarimeter (Graz, Austria). The semi-preparative HPLC was conducted on an Agilent technologies 1260 infinity instrument (California, USA) equipped with a DAD detector. The medium-pressure liquid chromatography (MPLC) was conducted on a Buchi Sepacore. Column chromatography (CC) was performed on silica gel (100–200 m, 200–300 m, 300–400 m; Qiaodao Marine Chemistry Co., Ltd. Qingdao, China), Sephadex LH-20 (Amersham Pharmacia Biotech AB, Uppsala, Sweden), and ODS (octadecylsilyl; 50 μm, Daiso), respectively.

### 3.2. Fungal Identification, Fermentation, and Extraction

The fungus strain *Penicillium viridicatum* was isolated from the deep-sea sediment collected in 2005 at a depth of 3039 m in the Atlantic. It was cultivated on PDA plates at 25 °C for 3 days. Then, the fresh mycelia were sliced into squares (0.5 × 0.5 × 0.5 cm^3^) and incubated in seed medium (PDB) on a rotating shaker (160 rpm). Large fermentation was carried out in 100 Erlenmeyer flasks (1 L), with each containing rice (200 g) and tape water (120 mL, 3% marine salt). After incubation for 30 days at 25 °C, the fermented broth was extracted three times with EtOAc. The organic solvent was evaporated to dryness and dissolved in MeOH. The filtrate was concentrated under reduced pressure to give a crude extract (53.4 g).

### 3.3. Isolation and Purification

The crude extract was subjected to MPLC (460 mm × 46 mm) over silica gel (200–300 mesh) using gradient PE/EtOAc (100% → 0%, 8 h, 10 mL/min) to provide four fractions (Fr.1−Fr.4). Fraction Fr.2 (18.1 g) was further separated by CC over ODS (460 mm × 26 mm, MeOH-H_2_O, 5% → 50%, 1 h, 50% → 100%, 8 h, 10 mL/min) to give 15 subfractions (Fr.2.1–Fr.2.15). Subfraction Fr.2.4 (2.0 g), Fr.2.6 (130.3 mg), and Fr.2.14 (340.5 mg) were subsequently separated by CC over Sephadex LH-20 (150 cm × 2 cm, MeOH), silica gel (46 mm × 20 mm, PE-EtOAc), and PTLC (CH_2_Cl_2_-MeOH) to yield **7** (5.2 mg), **8** (12.5 mg), and **11** (37.6 mg). Compounds **6** (3.4 mg), **9** (6.1 mg), and **10** (11.3 mg) were separated by CC over Sephadex LH-20 (150 cm × 2 cm, MeOH), semi-prep HPLC (C-18, chiral column C4-5, 10 mm × 250 mm, MeOH-H_2_O, 60% → 100%, 3 mL/min), and Prep. TLC (CH_2_Cl_2_-MeOH) from subfraction Fr.2.9 (439.0 mg), Fr.2.10 (234.5 mg), and Fr.2.15 (80.0 mg), respectively. Fraction Fr.3 (14.1 g) was isolated by reversed-phase MPLC (460 mm × 26 mm, MeOH-H_2_O, 60% → 100%, 8 h, 10 mL/min) and Sephadex LH-20 (150 cm × 2 cm, MeOH), followed by semi-prep HPLC (5-PFP, 10 mm × 250 mm, MeOH-H_2_O, 50% → 80%, 3 mL/min) to yield **12** (37.3 mg). Fraction Fr.4 (8.0 g) was further separated by CC over ODS (460 mm × 15 mm, MeOH-H_2_O, 5% → 20%, 1 h, 20% → 90%, 8 h, 10 mL/min) to give seven subfractions (Fr.4.1–Fr.4.7). Compounds **3** (8.9 mg), **1** (9.1 mg), **2** (33.4 mg), **4** (5.1 mg), and **5** (6.2 mg) were separated by CC over Sephadex LH-20 (150 cm × 2 cm, MeOH) and Prep. TLC (CH_2_Cl_2_-MeOH, 10:1, 10:1, 15:1, 15:1, 20:1, *v*/*v*) from fraction Fr.4, respectively. Subfraction Fr.4.5 was separated by CC over Sephadex LH-20 (2 cm × 150 cm, MeOH), semi-prep HPLC (chiral column C4-5, 10 mm × 250 mm, MeOH-H_2_O, 50% → 80%, 3 mL/min), and Prep. TLC to yield **13** (2.8 mg).

*Viridecalin A (**1**)*: White solid; [*α*${]}_{D}^{25}$ +78.0 (c 0.1, MeOH); ^1^H and ^13^C NMR data, see Table 1. HRESIMS *m*/*z* 331.1879 [M + Na]^+^ (calcd for C_18_H_28_O_4_Na, 331.1885).

*Viridecalin B (**2**)*: Colorless oil; [*α*${]}_{D}^{25}$ +71.0 (*c* 0.1, MeOH); ^1^H and ^13^C NMR data, see Table 1. HRESIMS *m*/*z* 331.1879 [M + Na]^+^ (calcd for C_18_H_28_O_4_Na, 331.1885).

*Viridecalin C (**3**):* White solid; [*α*${]}_{D}^{25}$ +100 (*c* 0.1, MeOH); ^1^H and ^13^C NMR data, see Table 1. HRESIMS *m*/*z* 363.1768 [M + Na]^+^ (calcd for C_18_H_28_O_6_Na, 363.1778).

*Viridecalin D (**4**)*: Colorless oil; [*α*${]}_{D}^{25}$ +87.0 (*c* 0.1, MeOH); ^1^H and ^13^C NMR data, see Table 2. HRESIMS *m*/*z* 363.2139 [M + Na]^+^ (calcd for C_19_H_32_O_5_Na, 363.2147).

*Viridecalin E (**5**)*: Colorless oil; [*α*${]}_{D}^{25}$ +44.0 (*c* 0.1, MeOH); ^1^H and ^13^C NMR data, see Table 2. HRESIMS *m*/*z* 363.2149 [M + Na]^+^ (calcd for C_19_H_32_O_5_Na, 363.2147).

*Viridecalin F (**6**)*: Colorless oil; [*α*${]}_{D}^{25}$ +18.0 (*c* 0.1, MeOH); ^1^H and ^13^C NMR data, see Table 2. HRESIMS *m*/*z* 339.1572 [M + Na]^+^ (calcd for C_19_H_24_O_4_Na, 339.1572).

*Viridecalin G (**7**)*: Colorless oil; [*α*${]}_{D}^{25}$ +62.0 (*c* 0.2, MeOH); ^1^H and ^13^C NMR data, see Table 2 HRESIMS *m*/*z* 275.1676 [M + Na]^+^ (calcd for C_15_H_24_O_3_Na, 275.1623).

### 3.4. Luciferase Reporter Assay

H1299 cells (1 × 10^3^) were inoculated into 96-well plates and cultured for 24 h. Transfection was performed using EZ Trans (Life iLab Biotech, Shanghai, China) according to the manufacturer’s instructions. For transfection of 100 wells, 7500 ng of p53 R175H Expression Plasmid, 2500 ng of Luciferase Reporter Plasmid, and 120 ng of Renilla Plasmid were mixed thoroughly with 250 μL of Opti-MEM, while 30 μL of EZ Trans Transfection Reagent was mixed with 250 μL of Opti-MEM. Subsequently, the two parts of the mixture were mixed and incubated at room temperature for 10 min, and then, the mixture was added to 9 mL of room temperature DMEM medium and mixed thoroughly. All media in the 96-well plate was replaced with the mixture (100 μL per well). After transfection for 24 h, the cells were treated with the compounds for 24 h. The luciferase signal was detected using a luciferase assay kit (Vazyme, DL101-01, Nanjing, China). The luminescence signal of firefly luciferase for each sample was normalized to the luminescence signal of Renilla luciferase. Statistical analysis was performed using GraphPad Prism (version 10.4.1). Data are presented as the mean ± SD (n = 3), and significance was determined using one-way ANOVA. A *p*-value < 0.05 was considered statistically significant.

### 3.5. CCK8 Assay

A549 cells were cultured in DMEM supplemented with 10% fetal bovine serum and 1% antibiotics at 37 °C in a humidified incubator with 5% CO_2_ until they reached the logarithmic growth phase. A549 cells were seeded into a 96-well plate at a density of 5 × 10^3^ cells per well and allowed to adhere overnight. The following day, cells were pre-treated with the compounds of interest for 24 h. Then, cell viability was assessed using the CCK8 assay, and absorbance at 480 nm was recorded with a Multimode Mithras LB 943 microplate reader (Berthold, Bad Wildbad, Germany), following the manufacturer’s protocol.

## 4. Conclusions

In summary, our investigation on the chemistry of a deep-sea-derived *Penicillium viridicatum* MCCC 3A00265 led to the discovery, characterization, and structure elucidation (including absolute configuration) of seven new and six known decalin-type polyketides. All isolates share an octohydronaphthalene backbone, except that viridecalin F (**6**) possesses a rare naphthalene core. Of particular note, viridecalin A (**1**) exhibits a potent reactivation activity of mutant p53 protein at 10 μM and no cytotoxicity activity, superior to the known mutant p53 reactivator PRIMA-1 that served as the positive control in the same study. This finding underscores the value of marine-derived fungi as a source of novel bioactive compounds and provides a promising starting point for the development of targeted cancer therapies. The discovery of these compounds not only enriches the structural diversity of decalin-type polyketides but also highlights the untapped potential of deep-sea-derived fungi in drug discovery.

## Data Availability

The original data presented in the study are included in the article/Supplementary Material; further inquiries can be directed to the corresponding authors.

## Supplementary Materials

The following are available online at https://www.mdpi.com/article/10.3390/md23020087/s1, Figures S1–S69: One-dimensional and two-dimensional NMR spectra of all compounds.
