# Supplementary material for: Statins Diversity Revealed by the Deep-Sea-Derived Fungus Penicillium viridicatum
Source: Mar Drugs. 2025 Feb 17;23(2):87. doi: 10.3390/md23020087 (PMC11857571; doi:10.3390/md23020087)
Supplement: Supplementary file 1 [file marinedrugs-23-00087-s001.zip › marinedrugs-3465299-supplementary.pdf]

Legend of Figures

Figure S1. ^{1}H NMR (400 MHz, DMSO-d_6) spectrum of **1**......1
Figure S2. ^{13}C NMR (100 MHz, DMSO-d_6) spectrum of **1**......1
Figure S3. 2D HSQC (DMSO-d_6) spectrum of **1**......2
Figure S4. 2D COSY (DMSO-d_6) spectrum of **1**......2
Figure S5. 2D HMBC (DMSO-d_6) spectrum of **1**......3
Figure S6. 2D NOESY (DMSO-d_6) spectrum of **1**......3
Figure S7. HRMS spectrum and measurement of **1**......4
Figure S8. Specific rotation data of compound **1**......4
Figure S9. ^{1}H NMR (400 MHz, DMSO-d_6) spectrum of **2**......5
Figure S10. ^{13}C NMR (100 MHz, DMSO-d_6) spectrum of **2**......5
Figure S11. 2D HSQC (DMSO-d_6) spectrum of **2**......6
Figure S12. 2D COSY (DMSO-d_6) spectrum of **2**......6
Figure S13. 2D HMBC (DMSO-d_6) spectrum of **2**......7
Figure S14. 2D NOESY (DMSO-d_6) spectrum of **2**......7
Figure S15. HRMS spectrum and measurement of **2**......8
Figure S16. Specific rotation data of compound **2**......8
Figure S17. ^{1}H NMR (400 MHz, DMSO-d_6) spectrum of **3**......9
Figure S18. ^{13}C NMR (100 MHz, DMSO-d_6) spectrum of **3**......9
Figure S19. 2D HSQC (DMSO-d_6) spectrum of **3**......10
Figure S20. 2D COSY (DMSO-d_6) spectrum of **3**......10
Figure S21. 2D HMBC (DMSO-d_6) spectrum of **3**......11
Figure S22. 2D NOESY (DMSO-d_6) spectrum of **3**......11
Figure S23. HRMS spectrum and measurement of **3**......12
Figure S24. Specific rotation data of compound **3**......12
Figure S25. ^{1}H NMR (400 MHz, DMSO-d_6) spectrum of **4**......13
Figure S26. ^{13}C NMR (100 MHz, DMSO-d_6) spectrum of **4**......13
Figure S27. 2D HSQC (DMSO-d_6) spectrum of **4**......14
Figure S28. 2D COSY (DMSO-d_6) spectrum of **4**......14
Figure S29. 2D HMBC (DMSO-d_6) spectrum of **4**......15
Figure S30. 2D NOESY (DMSO-d_6) spectrum of **4**......15
Figure S31. HRMS spectrum and measurement of **4**......16
Figure S32. Specific rotation data of compound **4**......16
Figure S33. ^{1}H NMR (400 MHz, DMSO-d_6) spectrum of **5**......17
Figure S34. ^{13}C NMR (100 MHz, DMSO-d_6) spectrum of **5**......17

Figure S35. 2D HSQC (DMSO-d_6) spectrum of **5**. 18
Figure S36. 2D COSY (DMSO-d_6) spectrum of **5**. 18
Figure S37. 2D HMBC (DMSO-d_6) spectrum of **5**. 19
Figure S38. 2D NOESY (DMSO-d_6) spectrum of **5**. 19
Figure S39. HRMS spectrum and measurement of **5**. 20
Figure S40. Specific rotation data of compound **5**. 20
Figure S41. ^{1}H NMR (400 MHz, DMSO-d_6) spectrum of **6**. 21
Figure S42. ^{13}C NMR (100 MHz, DMSO-d_6) spectrum of **6**. 21
Figure S43. 2D HSQC (DMSO-d_6) spectrum of **6**. 22
Figure S44. 2D COSY (DMSO-d_6) spectrum of **6**. 22
Figure S45. 2D HMBC (DMSO-d_6) spectrum of **6**. 23
Figure S46. 2D NOESY (DMSO-d_6) spectrum of **6**. 23
Figure S47. HRMS spectrum and measurement of **6**. 24
Figure S48. Specific rotation data of compound **6**. 24
Figure S49. ^{1}H NMR (400 MHz, DMSO-d_6) spectrum of **7**. 25
Figure S50. ^{13}C NMR (100 MHz, DMSO-d_6) spectrum of **7**. 25
Figure S51. 2D HSQC (DMSO-d_6) spectrum of **7**. 26
Figure S52. 2D COSY (DMSO-d_6) spectrum of **7**. 26
Figure S53. 2D HMBC (DMSO-d_6) spectrum of **7**. 27
Figure S54. 2D NOESY (DMSO-d_6) spectrum of **7**. 27
Figure S55. Specific rotation data of compound **7**. 28
Figure S56. ^{1}H NMR (400 MHz, MeOD) spectrum of **8**. 28
Figure S57. ^{13}C NMR (100 MHz, MeOD) spectrum of **8**. 29
Figure S58. ^{1}H NMR (400 MHz, MeOD) spectrum of **9**. 29
Figure S59. ^{13}C NMR (100 MHz, MeOD) spectrum of **9**. 30
Figure S60. ^{1}H NMR (400 MHz, $CDCl_3$) spectrum of **10**. 30
Figure S61. ^{13}C NMR (100 MHz, $CDCl_3$) spectrum of **10**. 31
Figure S62. ^{1}H NMR (400 MHz, $CDCl_3$) spectrum of **11**. 31
Figure S63. ^{13}C NMR (100 MHz, $CDCl_3$) spectrum of **11**. 32
Figure S64. ^{1}H NMR (400 MHz, MeOD) spectrum of **12**. 32
Figure S65. ^{13}C NMR (100 MHz, MeOD) spectrum of **12**. 33
Figure S66. ^{1}H NMR (400 MHz, MeOD) spectrum of **13**. 33
Figure S67. ^{13}C NMR (100 MHz, MeOD) spectrum of **13**. 34
Figure S68. Stacked ^{1}H NMR spectra of 6,13-di-*S*-Mosher ester (red) and 6,13-di-*R*-Mosher ester of compound **1**. 34
Figure S69. The cytotoxicity of compounds **1**–**7** against A549 cell lines. 35

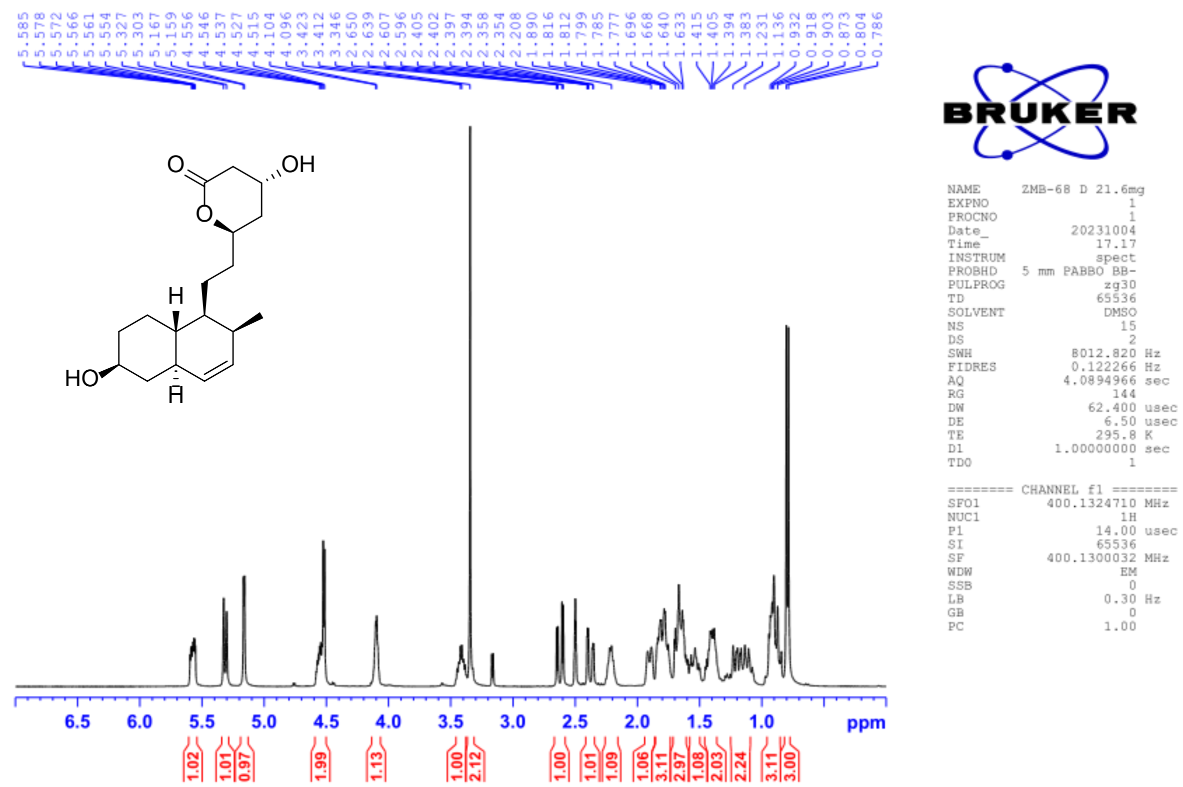

Figure S1. ^{1}H NMR (400 MHz, DMSO-d_6) spectrum of **1**.

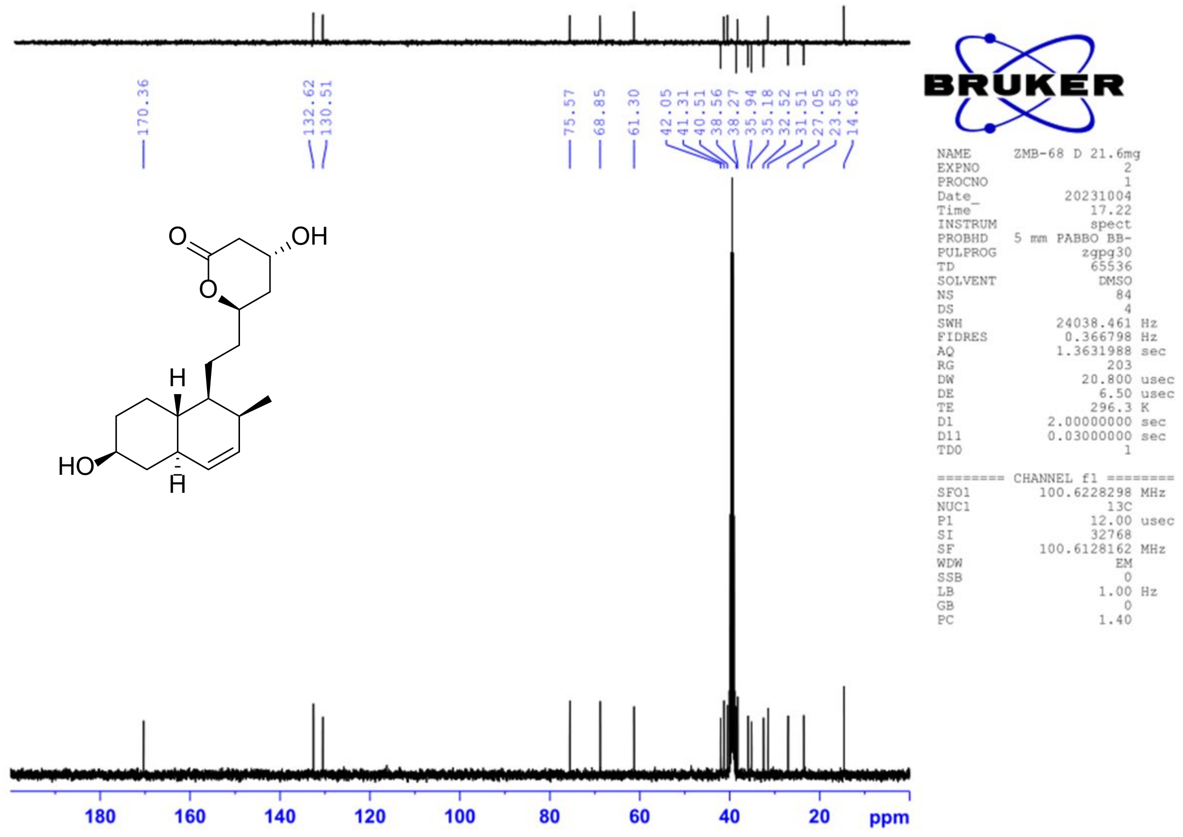

Figure S2. ^{13}C NMR (100 MHz, DMSO-d_6) spectrum of **1**.

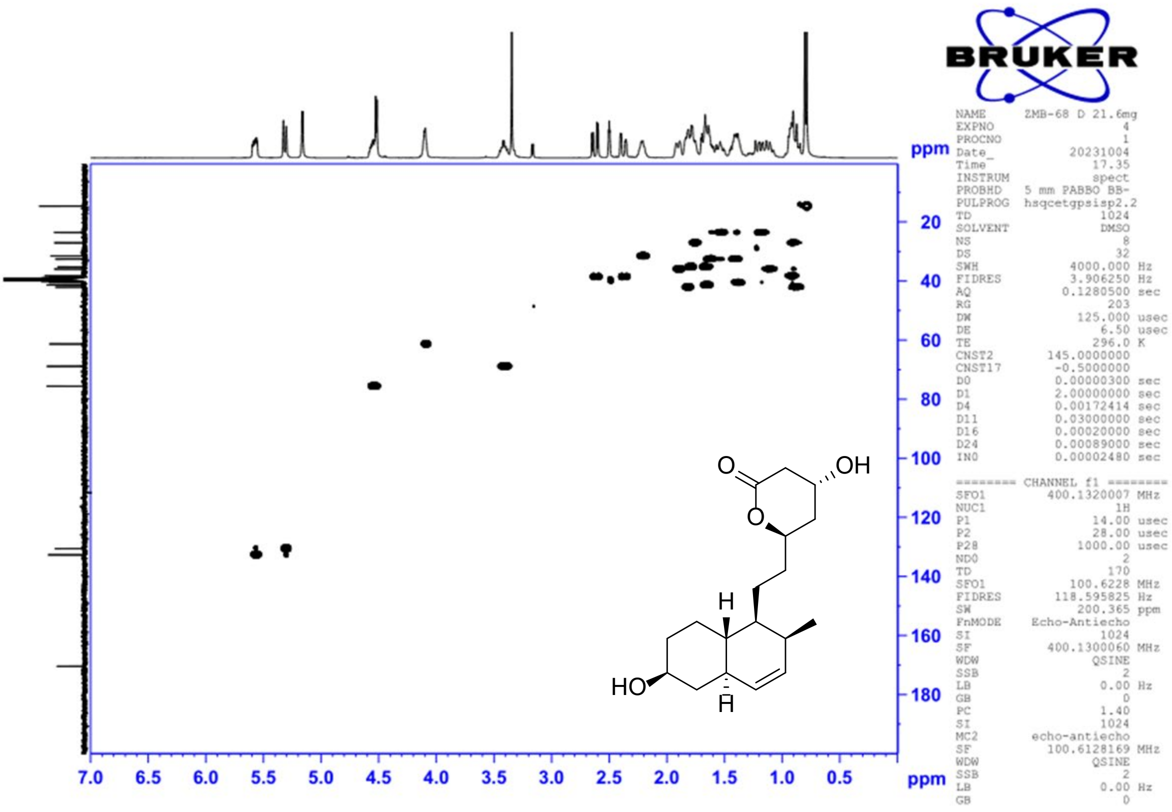

Figure S3. 2D HSQC (DMSO-d_6) spectrum of **1**.

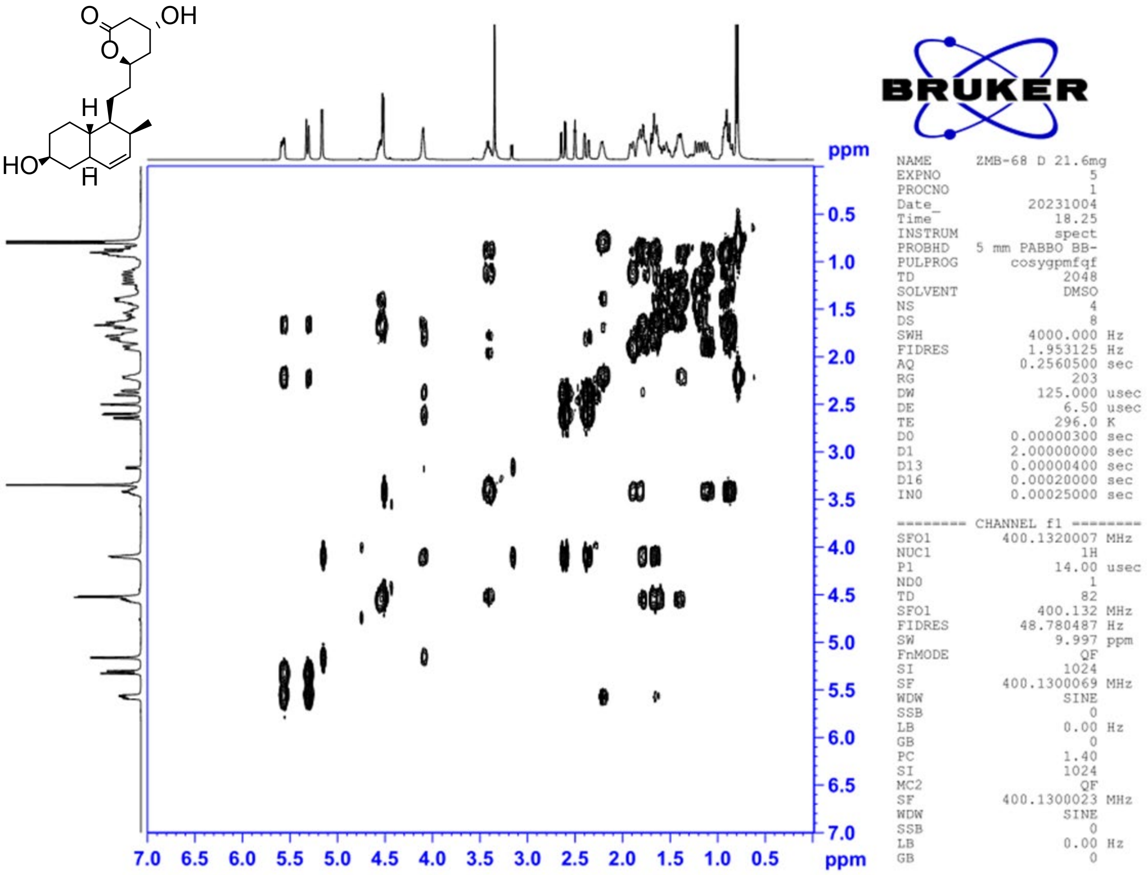

Figure S4. 2D COSY (DMSO-d_6) spectrum of **1**.

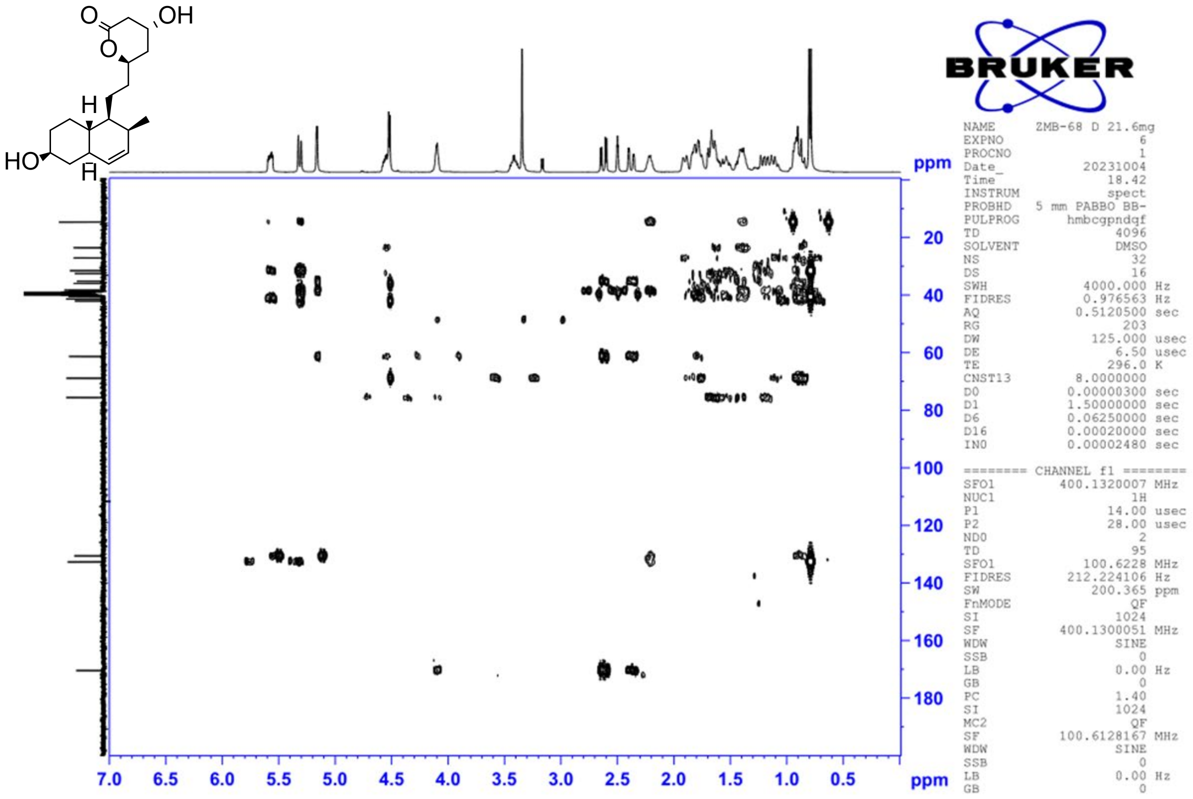

Figure S5. 2D HMBC (DMSO-d_6) spectrum of **1**.

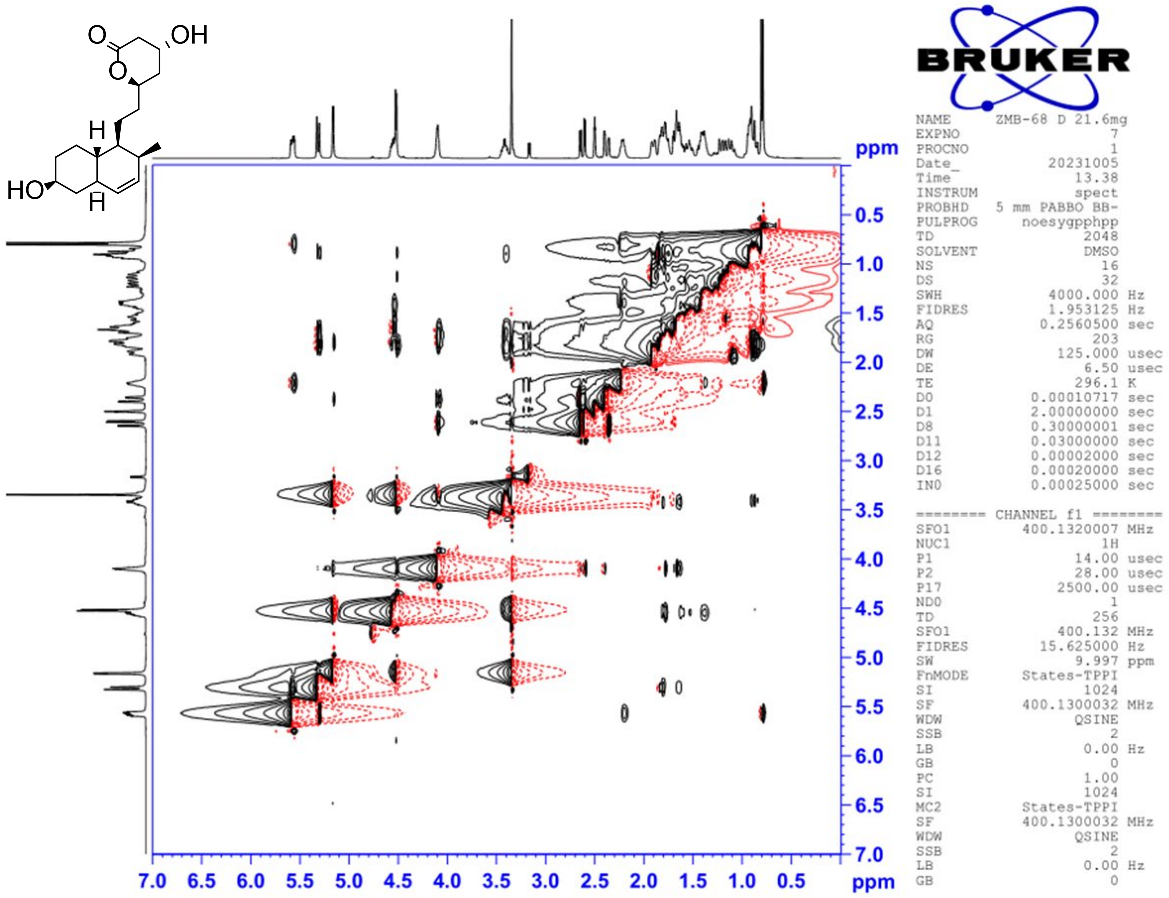

Figure S6. 2D NOESY (DMSO-d_6) spectrum of **1**.

Elemental Composition Report

Single Mass Analysis
Tolerance = 40.0 mDa / DBE: min = -1.5, max = 50.0
Element prediction: Off
Number of isotope peaks used for i-FIT = 3

Monoisotopic Mass, Even Electron Ions
22 formula(e) evaluated with 6 results within limits (up to 50 best isotopic matches for each mass)
Elements Used:
C: 0-18 H: 0-32 O: 0-7 Na: 0-1
ZMB-68 120 (0.468) Cm (90:165)
1: TOF MS ES+

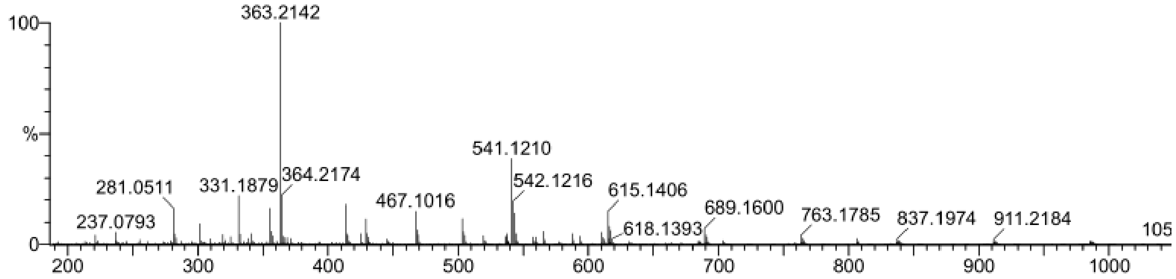

Minimum:				-1.5				
Maximum:		40.0	10.0	50.0				

Mass	Calc. Mass	mDa	PPM	DBE	i-FIT	Norm	Conf(%)	Formula
331.1879	331.1885	-0.6	-1.8	4.5	1701.4	0.287	75.07	C18 H28 O4 Na
	331.2121	-24.2	-73.1	2.5	1703.0	1.912	14.78	C17 H31 O6
	331.1521	35.8	108.1	5.5	1703.6	2.489	8.30	C17 H24 O5 Na
	331.1757	12.2	36.8	3.5	1705.3	4.190	1.51	C16 H27 O7
	331.2097	-21.8	-65.8	-0.5	1707.1	5.977	0.25	C15 H32 O6 Na
	331.1733	14.6	44.1	0.5	1708.1	7.003	0.09	C14 H28 O7 Na

Figure S7. HRMS spectrum and measurement of **1**.

Anton Paar GmbH
Anton Paar Strasse 10
8054 Graz
Austria

Anton Paar

Anton Paar Polarimeter - Measurement(s)

MCP 100
Software Version: 1.50.4098.87
Serial Number: 99032784

Unique Id 2308

- Sample Name: ZMB-68
- Date: 12/08/2023 - 03:07 PM
- Username: Administrator
- Sample State: Ok

- Measurement Mode: Specific Rotation
- Measurement Result: 78.000 °
- Concentration: 0.100 g/100ml
- Optical Rotation: 0.078 °
- Set Temperature: 25.0 °C
- Temperature: 25.0 °C

Figure S8. Specific rotation data of compound **1**.

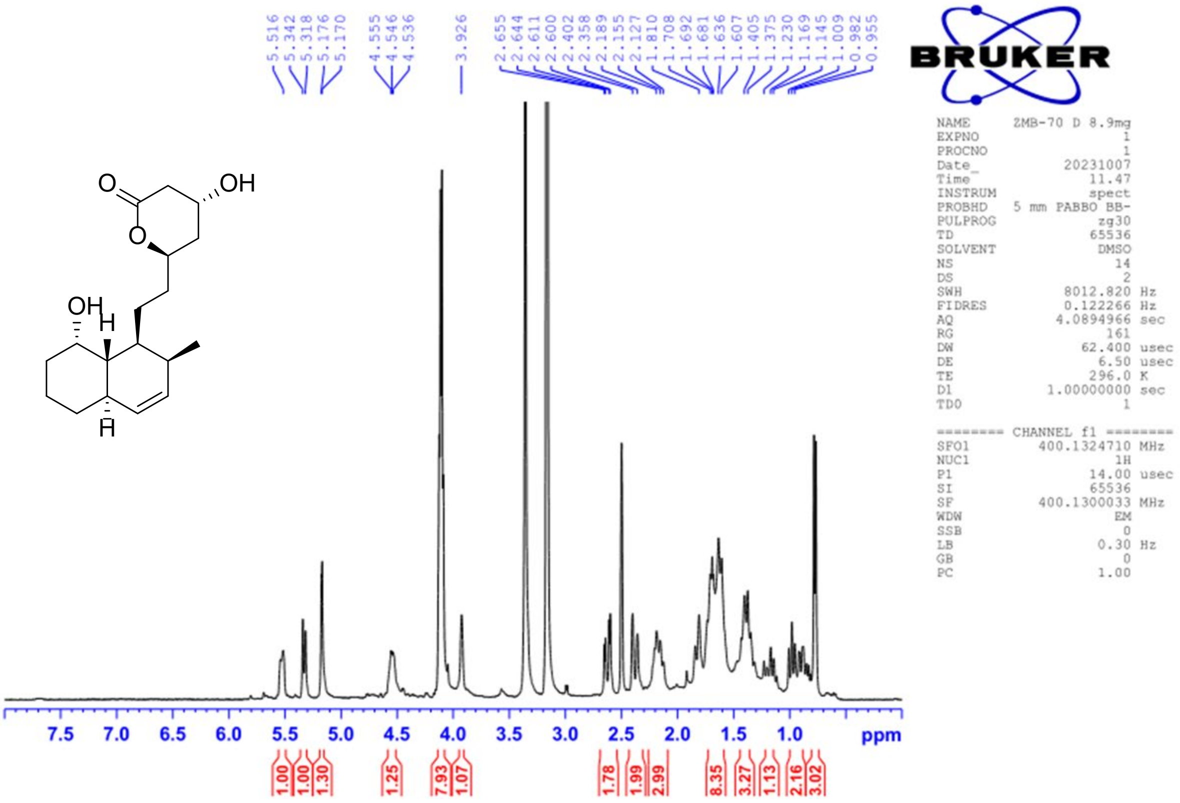

Figure S9. ^{1}H NMR (400 MHz, DMSO-d_6) spectrum of **2**.

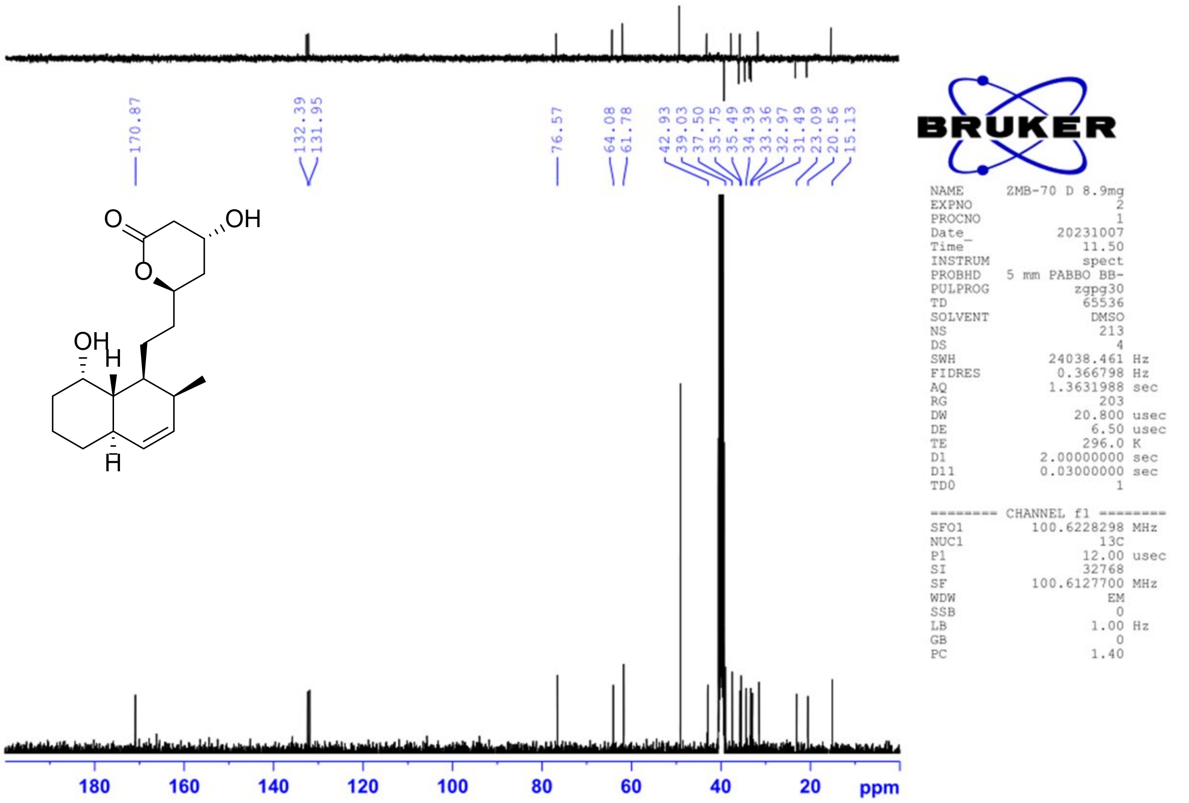

Figure S10. ^{13}C NMR (100 MHz, DMSO-d_6) spectrum of **2**.

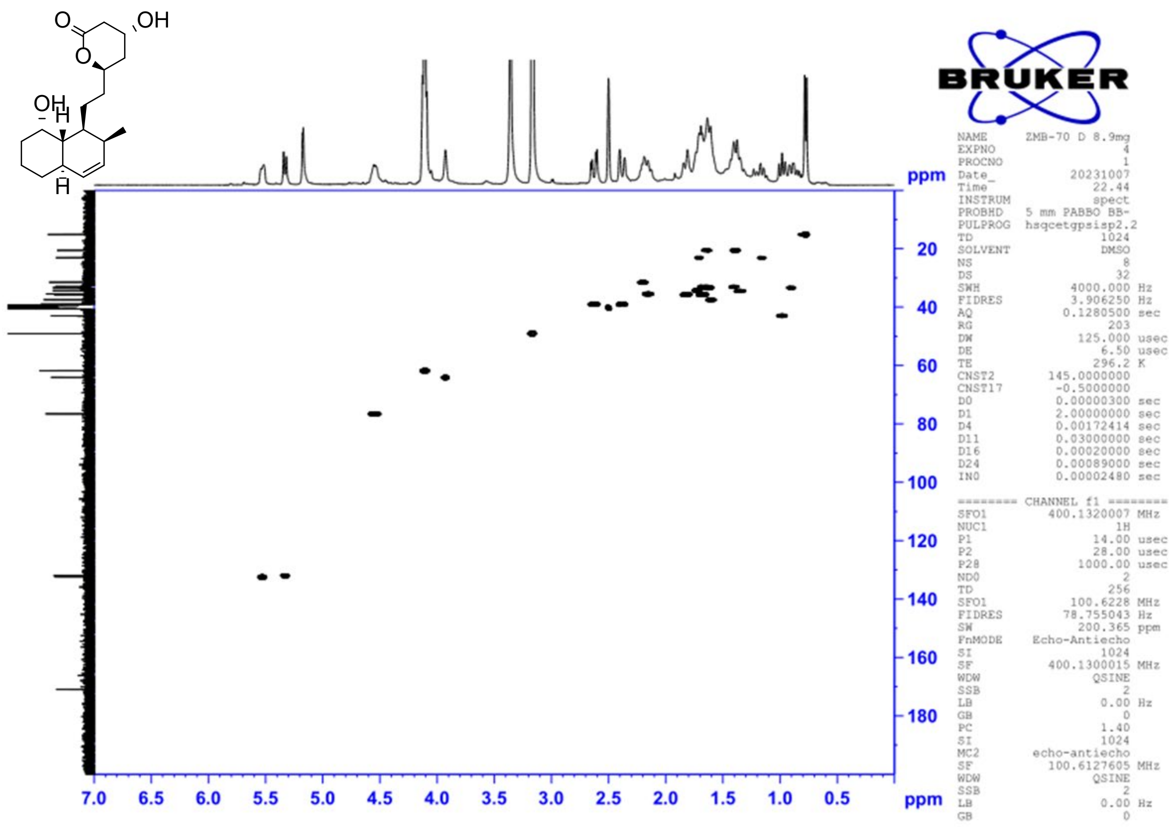

Figure S11. 2D HSQC (DMSO-d_6) spectrum of **2**.

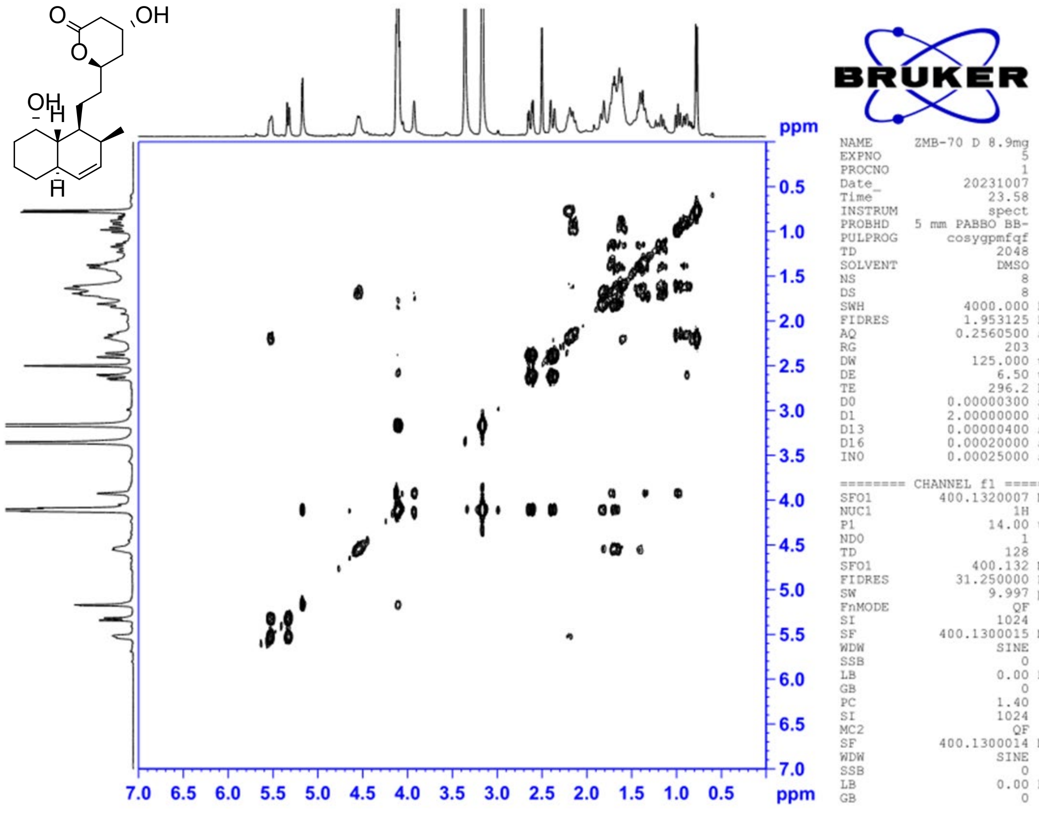

Figure S12. 2D COSY (DMSO-d_6) spectrum of **2**.

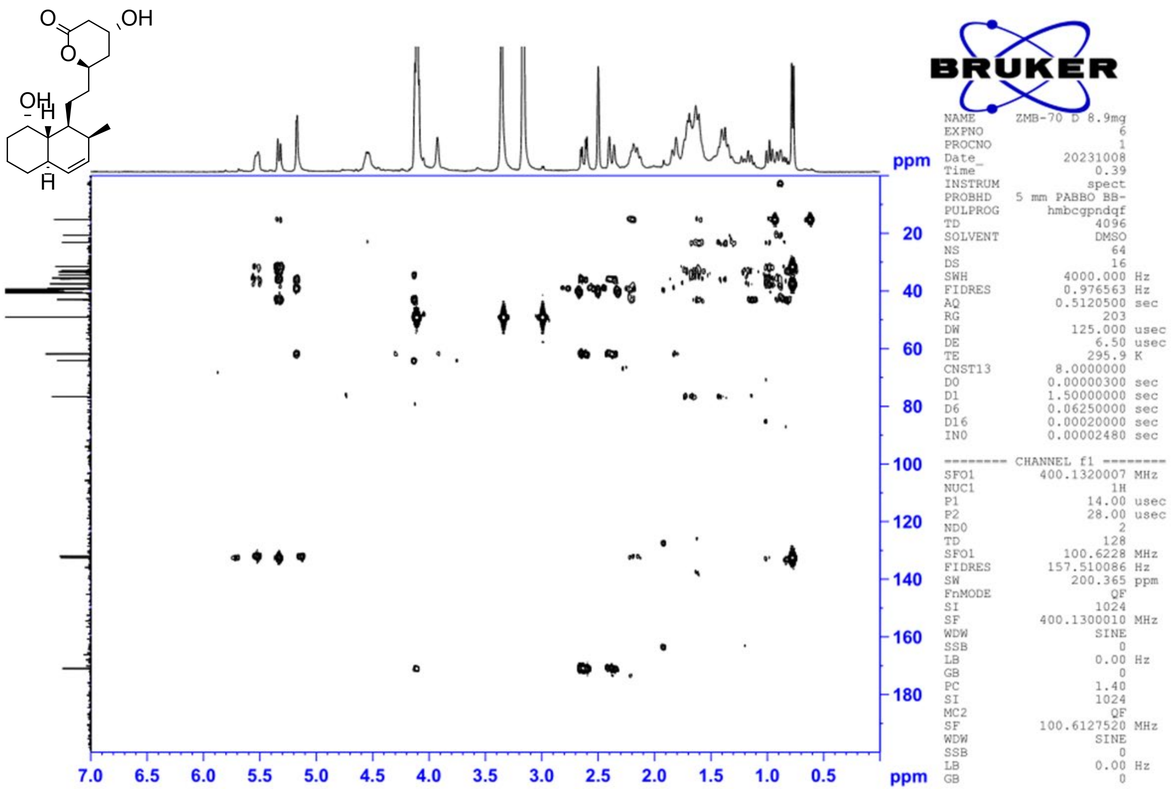

Figure S13. 2D HMBC (DMSO-d_6) spectrum of **2**.

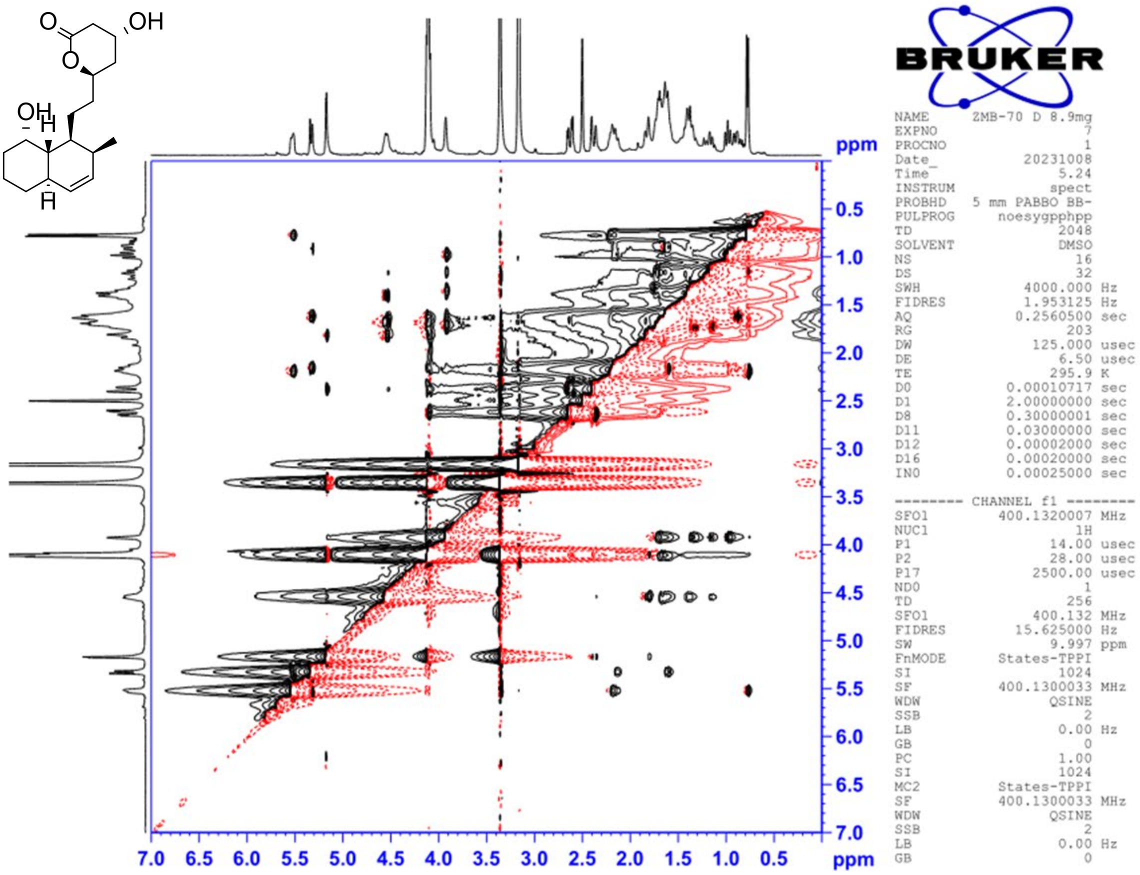

Figure S14. 2D NOESY (DMSO-d_6) spectrum of **2**.

Elemental Composition Report

Single Mass Analysis

Tolerance = 50.0 mDa / DBE: min = -1.5, max = 50.0
Element prediction: Off
Number of isotope peaks used for i-FIT = 3

Monoisotopic Mass, Even Electron Ions
11 formula(e) evaluated with 2 results within limits (up to 50 best isotopic matches for each mass)
Elements Used:
C: 15-18 H: 0-30 O: 0-5 Na: 0-1
ZMB-70 118 (0.461) Cm (101:142)
1: TOF MS ES+

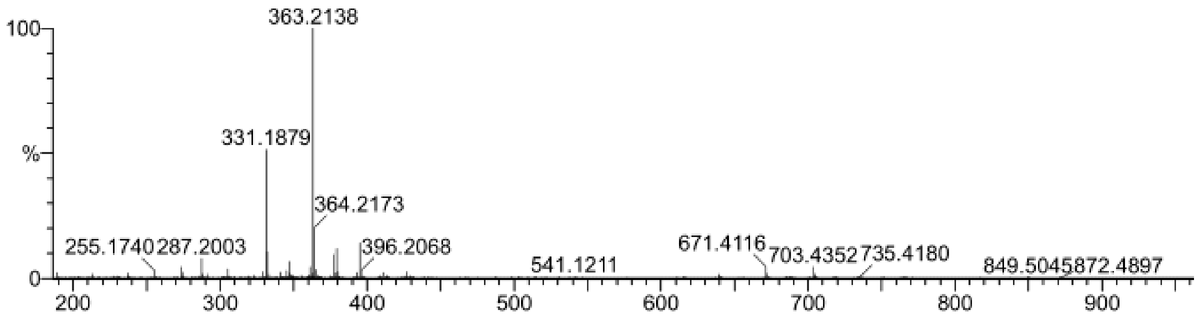

	Mass	Calc. Mass	mDa	PPM	DBE	i-FIT	Norm	Conf(%)	Formula
Minimum:					-1.5				
Maximum:			50.0	10.0	50.0				
	331.1879	331.1885	-0.6	-1.8	4.5	2013.0	0.008	99.19	C18 H28 O4 Na
		331.1521	35.8	108.1	5.5	2017.8	4.820	0.81	C17 H24 O5 Na

Figure S15. HRMS spectrum and measurement of **2**.

Anton Paar GmbH
Anton Paar Strasse 10
8054 Graz
Austria

Anton Paar Polarimeter - Measurement(s)

MCP 100
Software Version: 1.50.4098.87
Serial Number: 99032784

Unique Id 2309

- Sample Name: ZMB-70
- Date: 12/08/2023 - 03:16 PM
- Username: Administrator
- Sample State: Ok

- Measurement Mode: Specific Rotation
- Measurement Result: 71.000 °
- Concentration: 0.100 g/100ml
- Optical Rotation: 0.071 °
- Set Temperature: 25.0 °C
- Temperature: 25.0 °C

Figure S16. Specific rotation data of compound **2**.

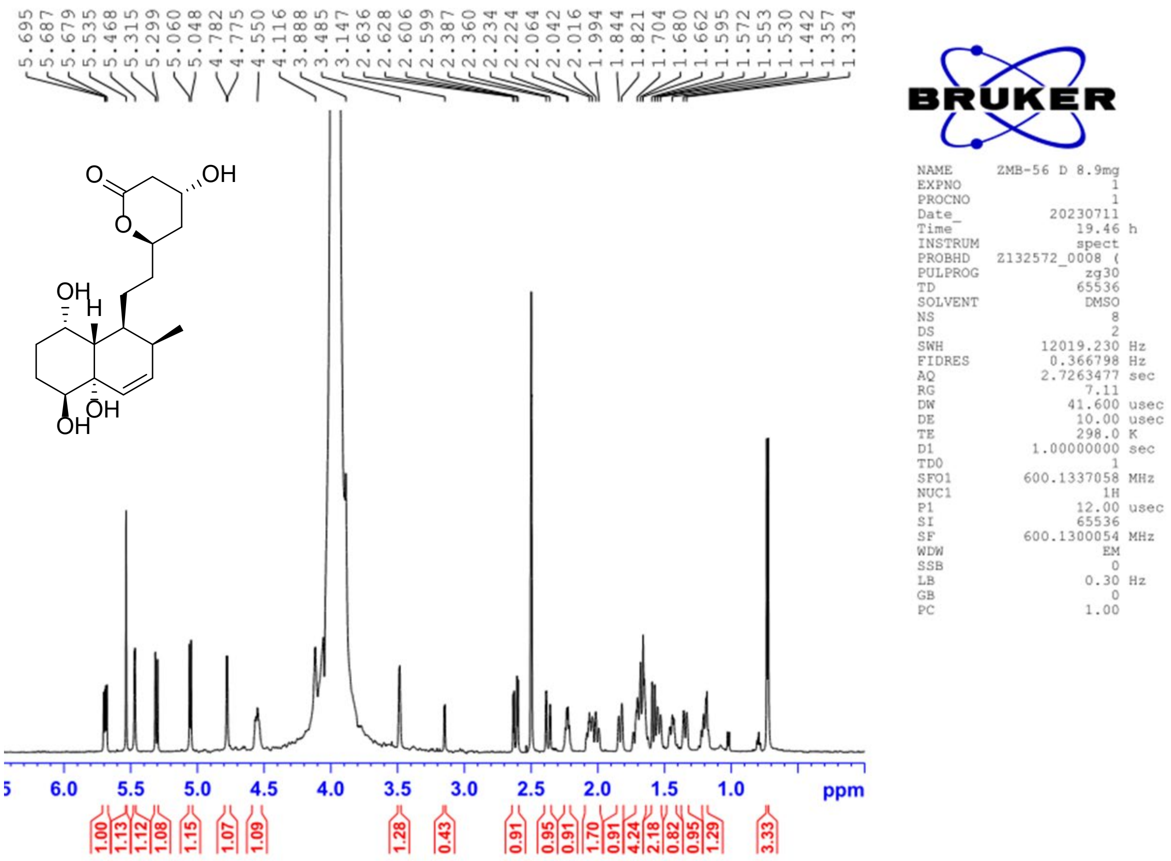

Figure S17. ^{1}H NMR (400 MHz, DMSO-d_6) spectrum of **3**.

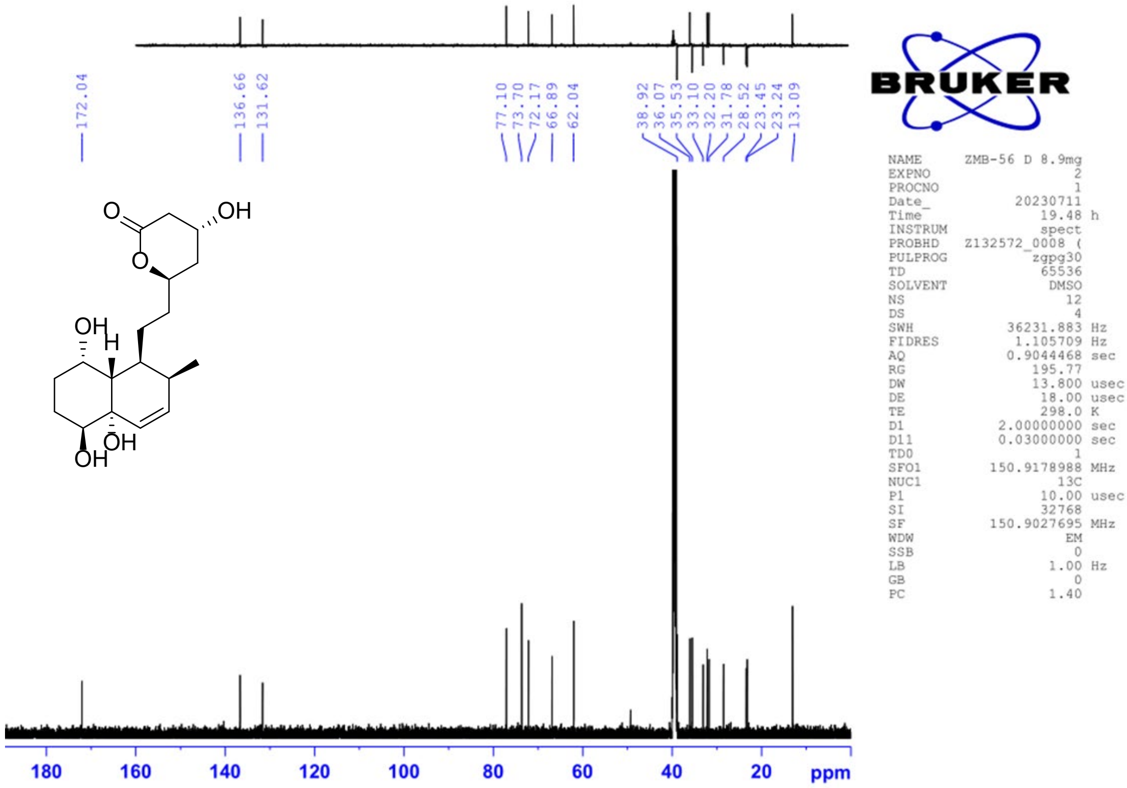

Figure S18. ^{13}C NMR (100 MHz, DMSO-d_6) spectrum of **3**.

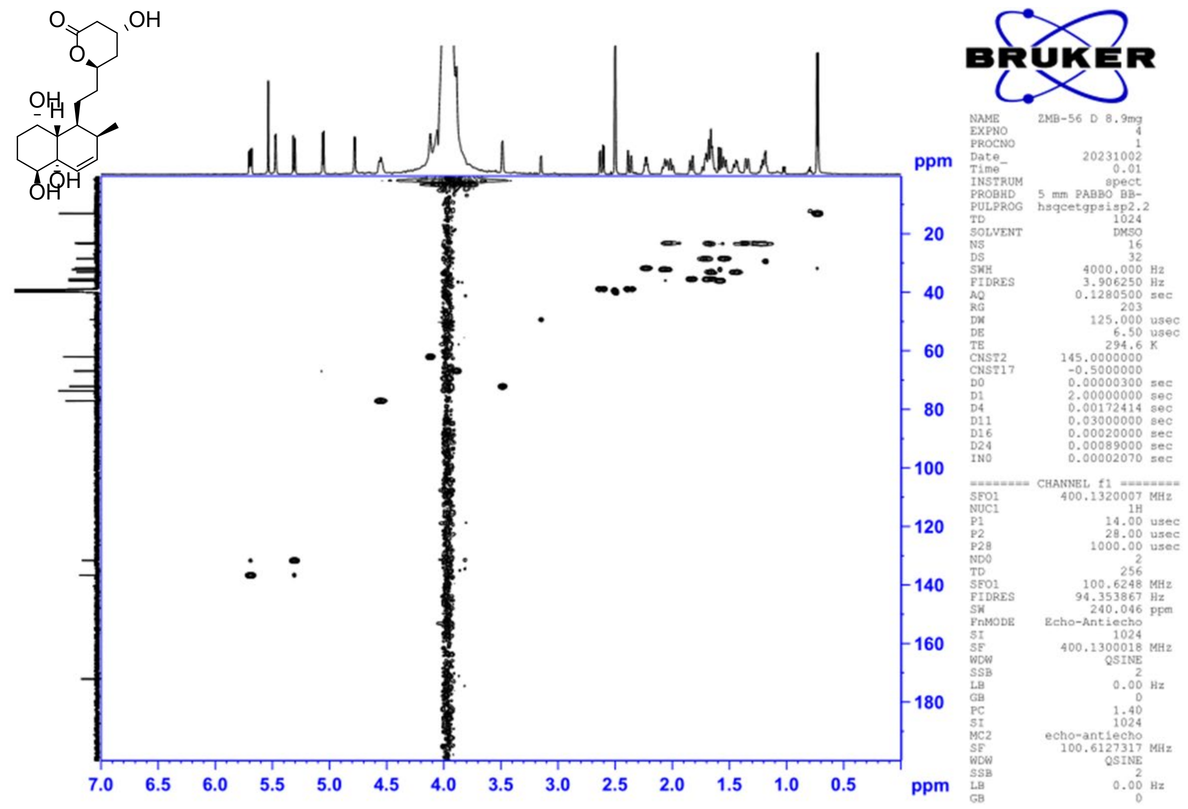

Figure S19. 2D HSQC (DMSO-d_6) spectrum of **3**.

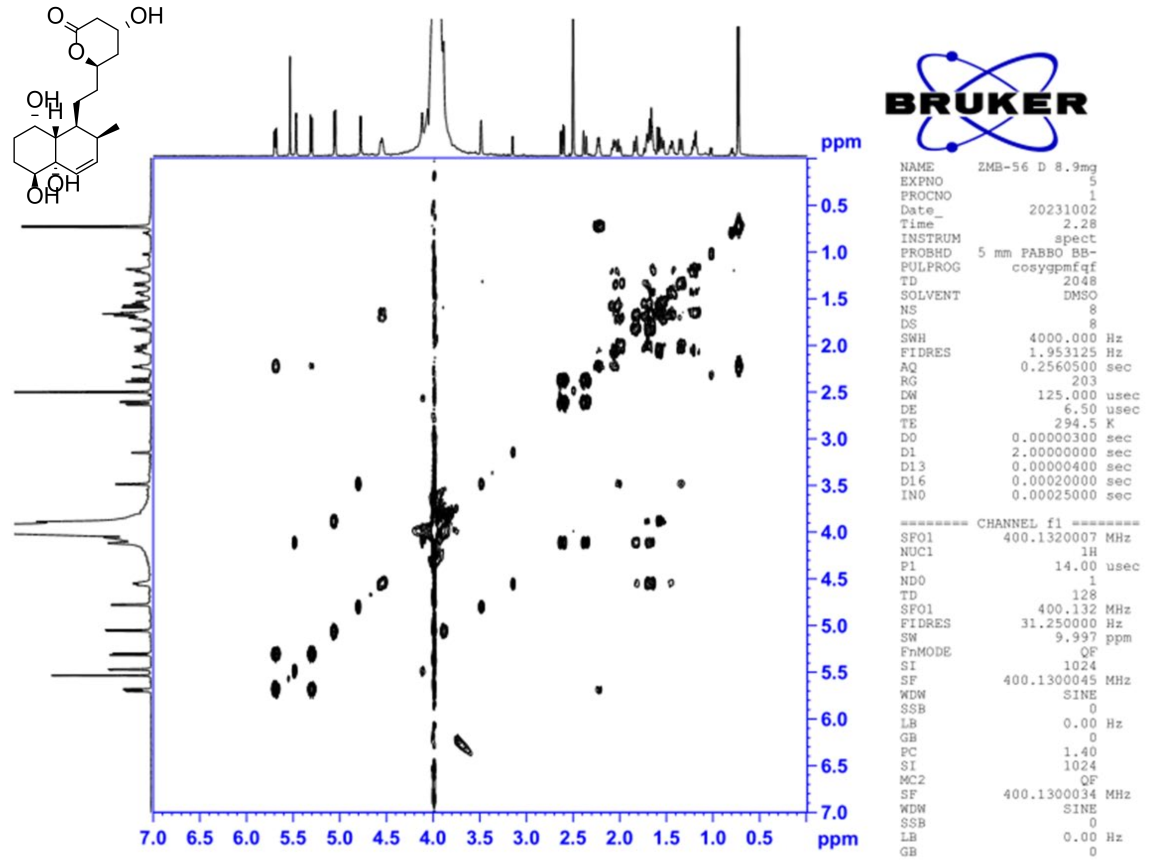

Figure S20. 2D COSY (DMSO-d_6) spectrum of **3**.

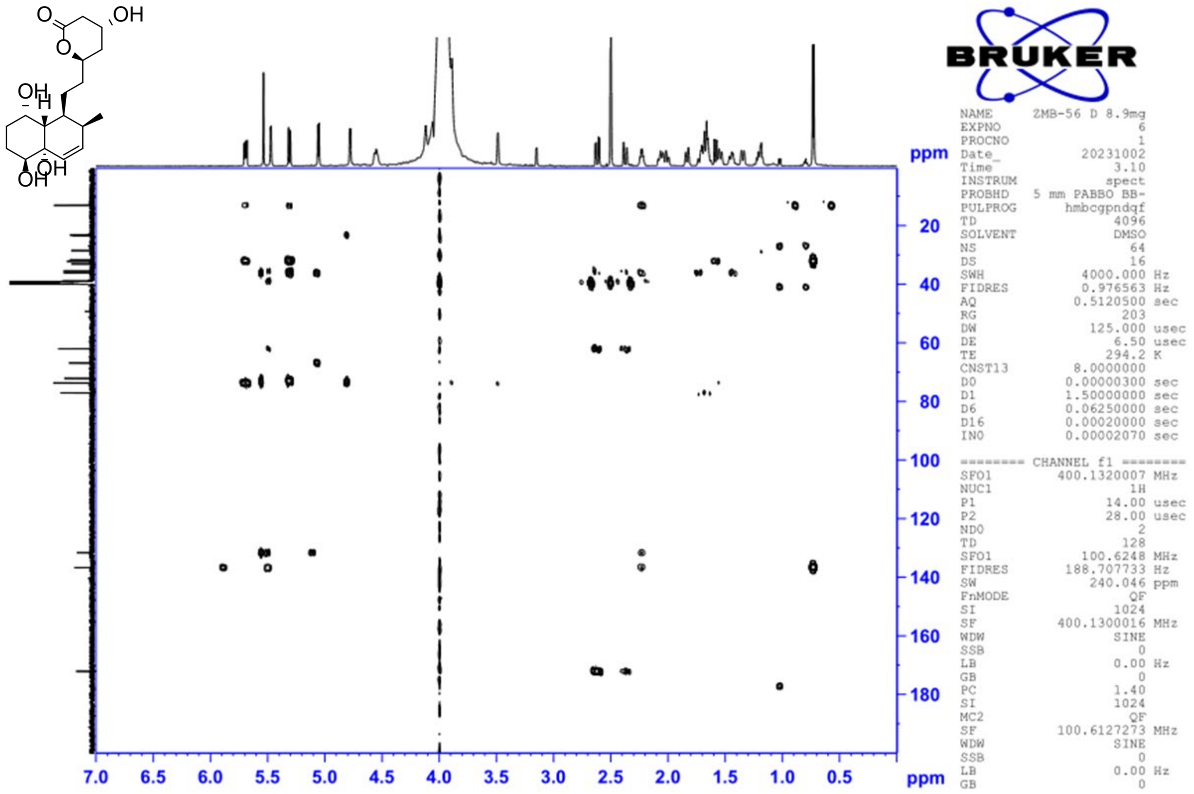

Figure S21. 2D HMBC (DMSO-d_6) spectrum of **3**.

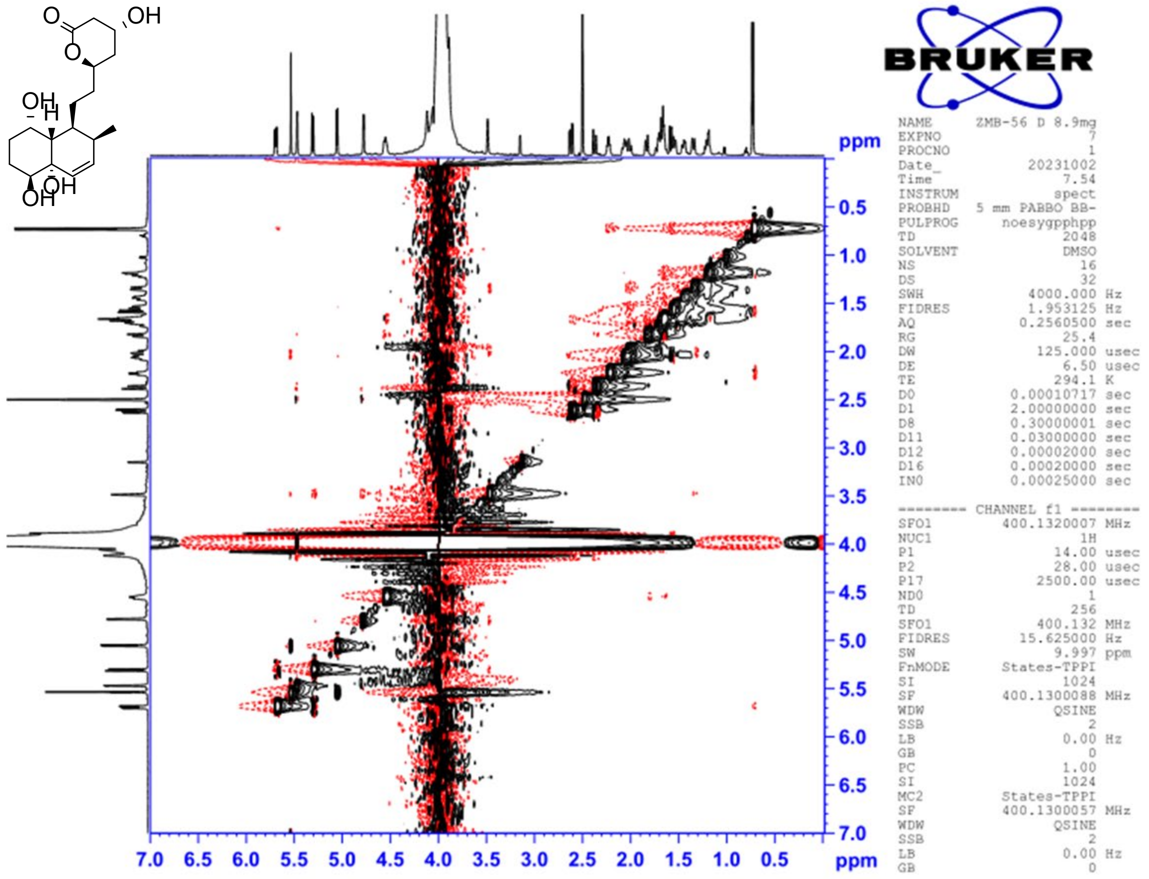

Figure S22. 2D NOESY (DMSO-d_6) spectrum of **3**.

Elemental Composition Report

Single Mass Analysis
Tolerance = 40.0 mDa / DBE: min = -1.5, max = 50.0
Element prediction: Off
Number of isotope peaks used for i-FIT = 3

Monoisotopic Mass, Even Electron Ions
1 formula(e) evaluated with 0 results within limits (up to 50 best isotopic matches for each mass)
Elements Used:
C: 0-16 H: 0-61 N: 0-4 O: 0-7 Na: 0-1
ZMB-56 127 (0.493) Cm (89:227)
1: TOF MS ES+

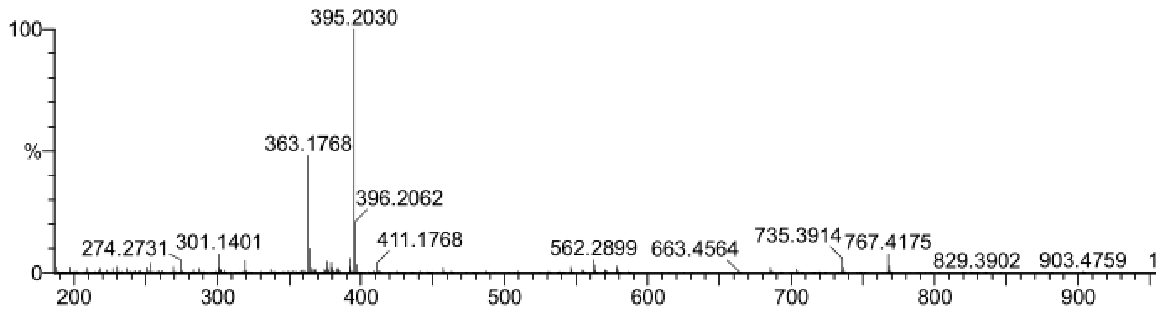

Minimum:			-1.5
Maximum:	40.0	10.0	50.0

Figure S23. HRMS spectrum and measurement of **3**.

Anton Paar GmbH
Anton Paar Strasse 10
8054 Graz
Austria

Anton Paar Polarimeter - Measurement(s)

MCP 100
Software Version: 1.50.4098.87
Serial Number: 99032784

Unique Id 2305

- Sample Name: ZMB-56
- Date: 12/08/2023 - 02:39 PM
- Username: Administrator
- Sample State: Ok

- Measurement Mode: Specific Rotation
- Measurement Result: 100.000 °
- Concentration: 0.100 g/100ml
- Optical Rotation: 0.100 °
- Set Temperature: 25.0 °C
- Temperature: 25.0 °C

Figure S24. Specific rotation data of compound **3**.

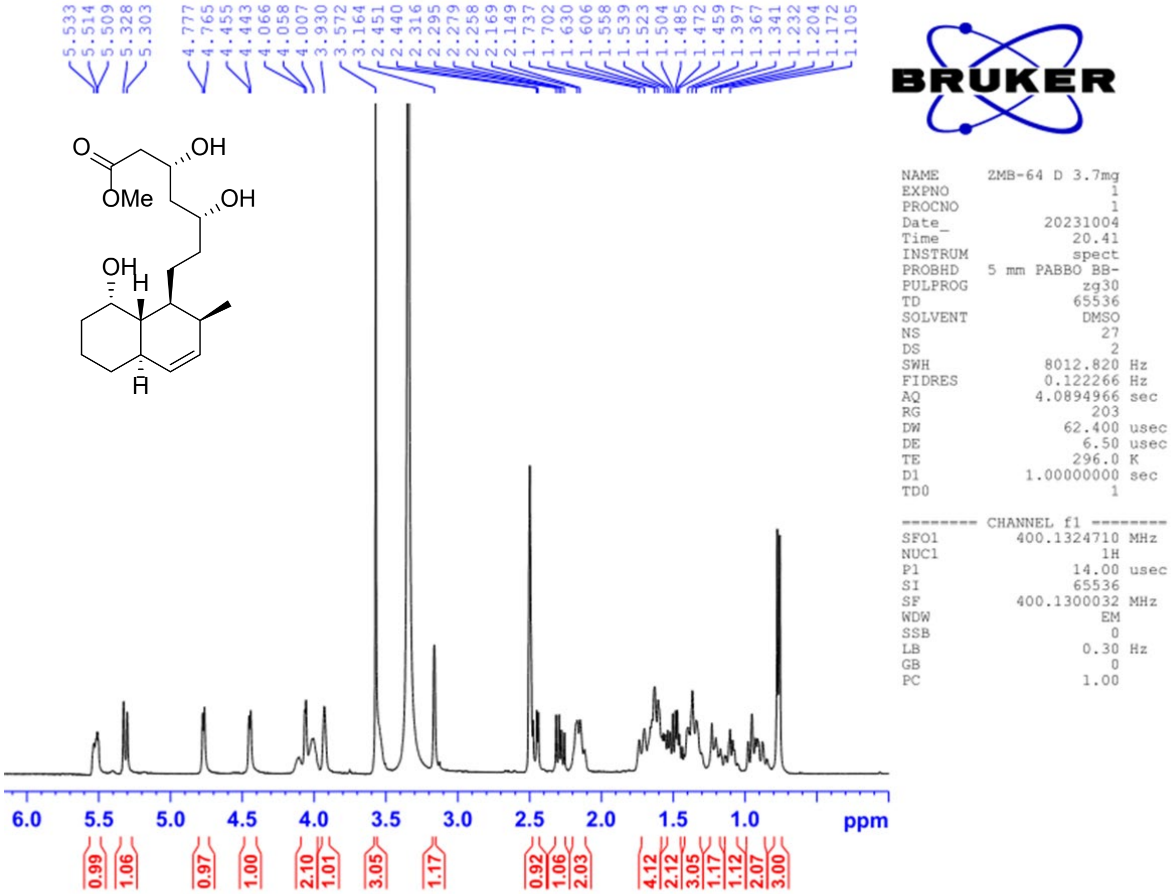

Figure S25. ^{1}H NMR (400 MHz, DMSO-d_6) spectrum of **4**.

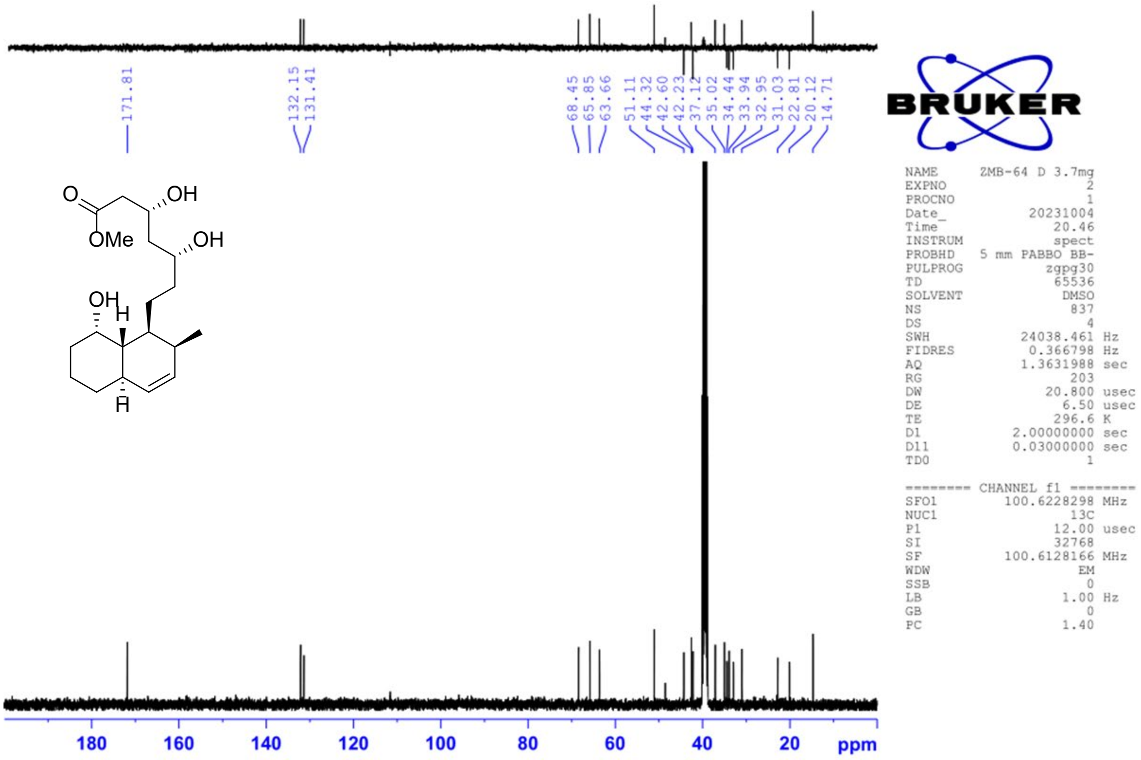

Figure S26. ^{13}C NMR (100 MHz, DMSO-d_6) spectrum of **4**.

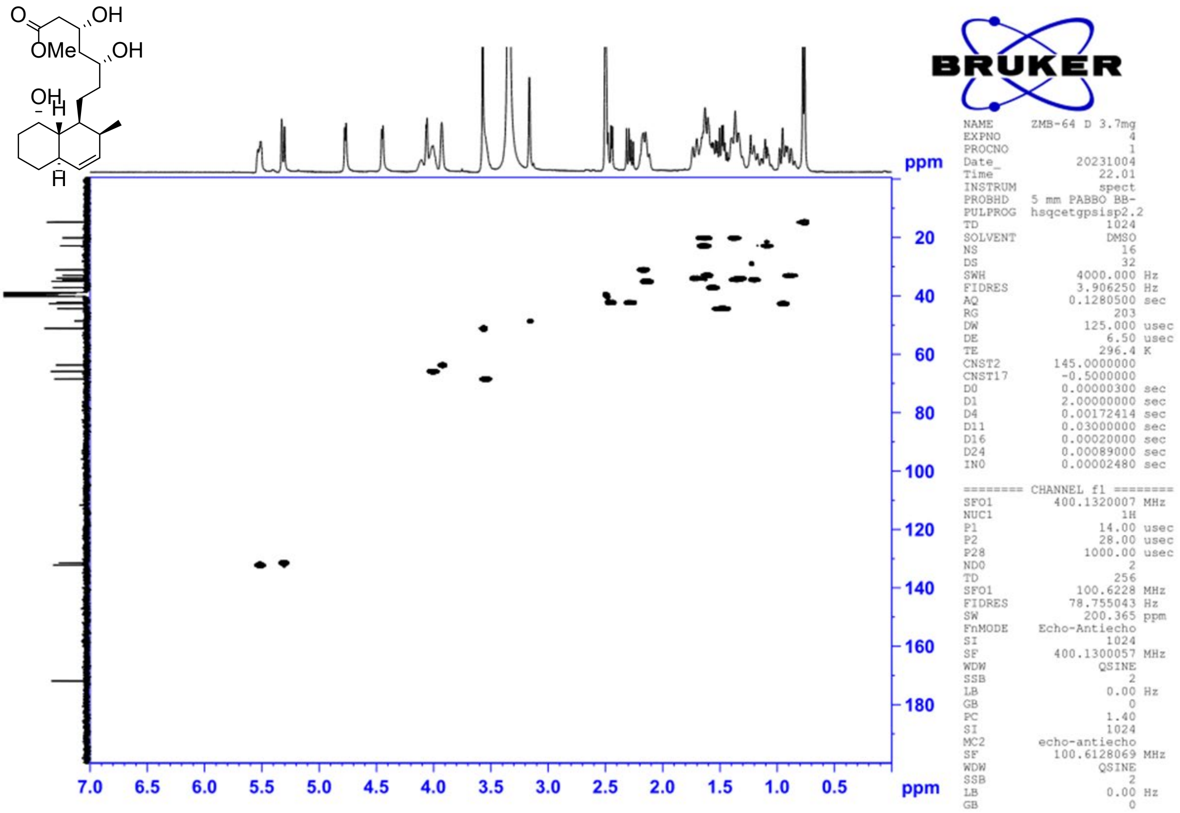

Figure S27. 2D HSQC (DMSO-d_6) spectrum of **4**.

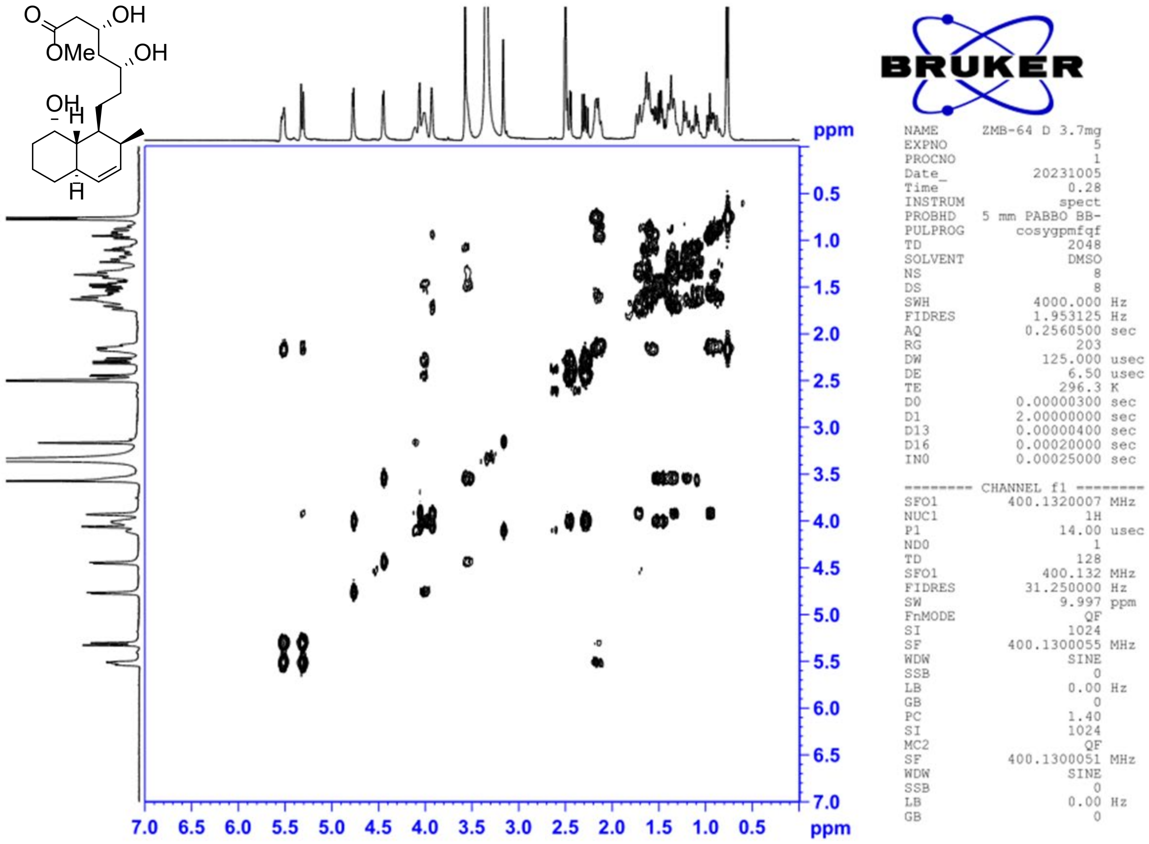

Figure S28. 2D COSY (DMSO-d_6) spectrum of **4**.

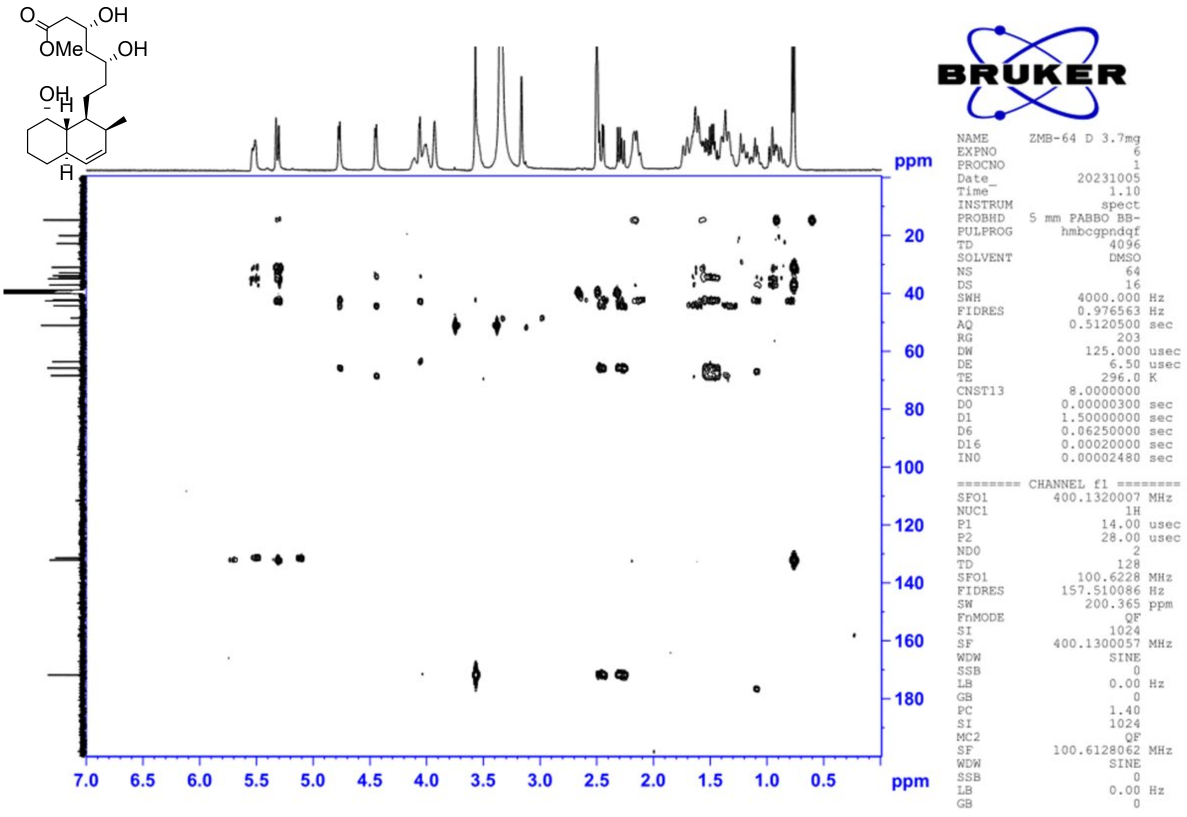

Figure S29. 2D HMBC (DMSO-d_6) spectrum of **4**.

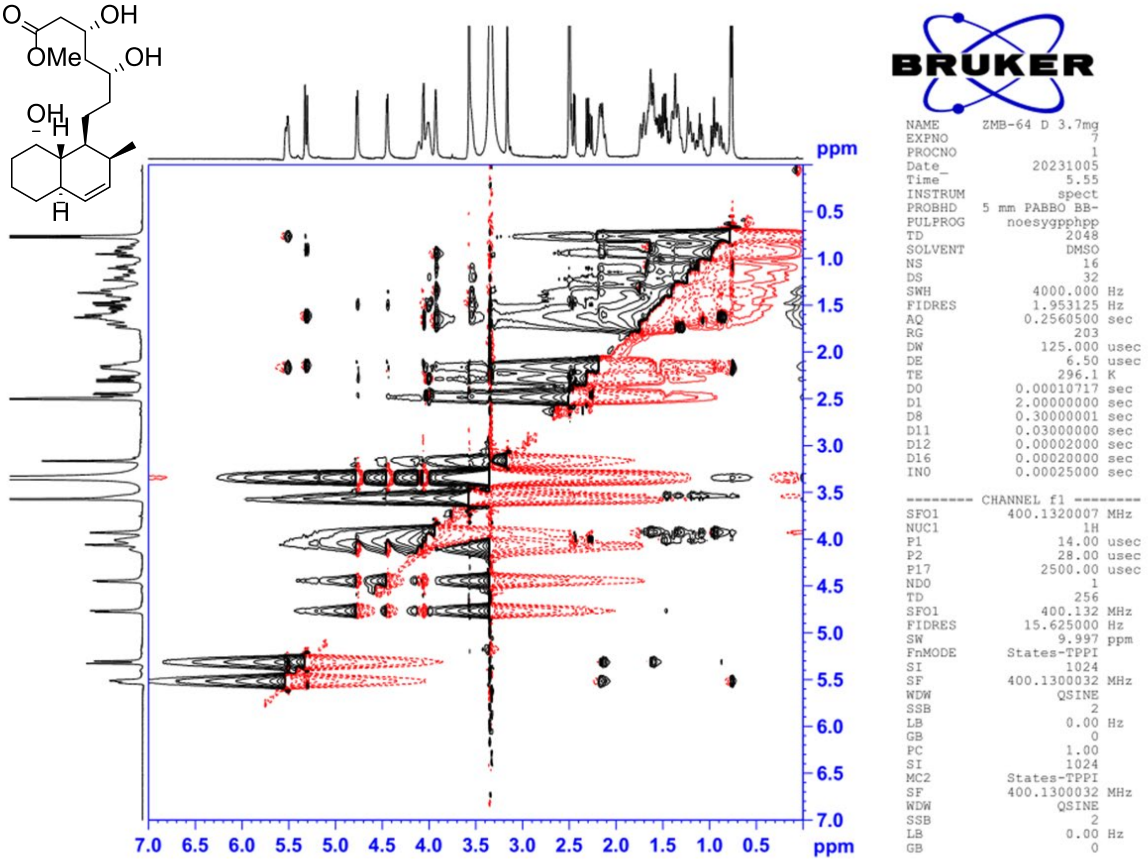

Figure S30. 2D NOESY (DMSO-d_6) spectrum of **4**.

Elemental Composition Report

Single Mass Analysis
Tolerance = 40.0 mDa / DBE: min = -1.5, max = 50.0
Element prediction: Off
Number of isotope peaks used for i-FIT = 3

Monoisotopic Mass, Even Electron Ions
23 formula(e) evaluated with 4 results within limits (up to 50 best isotopic matches for each mass)
Elements Used:
C: 0-19 H: 0-61 O: 0-7 Na: 0-1
ZMB-64 119 (0.465) Cm (93:142)
1: TOF MS ES+

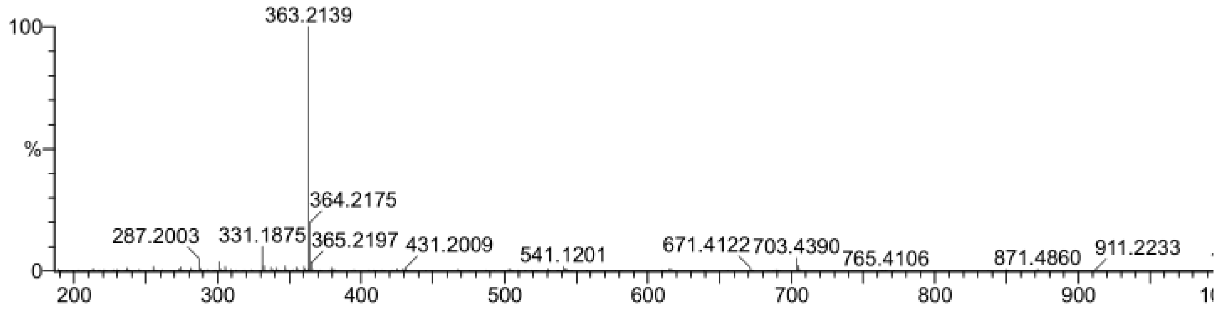

Minimum:				-1.5				
Maximum:		40.0	10.0	50.0				
Mass	Calc. Mass	mDa	PPM	DBE	i-FIT	Norm	Conf(%)	Formula
363.2139	363.1784	35.5	97.7	4.5	2253.7	0.405	66.73	C18 H28 O6 Na
	363.2383	-24.4	-67.2	1.5	2255.0	1.725	17.83	C18 H35 O7
	363.2147	-0.8	-2.2	3.5	2255.4	2.110	12.12	C19 H32 O5 Na
	363.2359	-22.0	-60.6	-1.5	2256.7	3.404	3.33	C16 H36 O7 Na

Figure S31. HRMS spectrum and measurement of **4**.

Anton Paar GmbH
Anton Paar Strasse 10
8054 Graz
Austria

Anton Paar

Anton Paar Polarimeter - Measurement(s)
MCP 100
Software Version: 1.50.4098.87
Serial Number: 99032784

Unique Id 2306

- Sample Name: ZMB-64
- Date: 12/08/2023 - 02:44 PM
- Username: Administrator
- Sample State: Ok

- Measurement Mode: Specific Rotation
- Measurement Result: 87.000 °
- Concentration: 0.100 g/100ml
- Optical Rotation: 0.088 °
- Set Temperature: 25.0 °C
- Temperature: 25.0 °C

Figure S32. Specific rotation data of compound **4**.

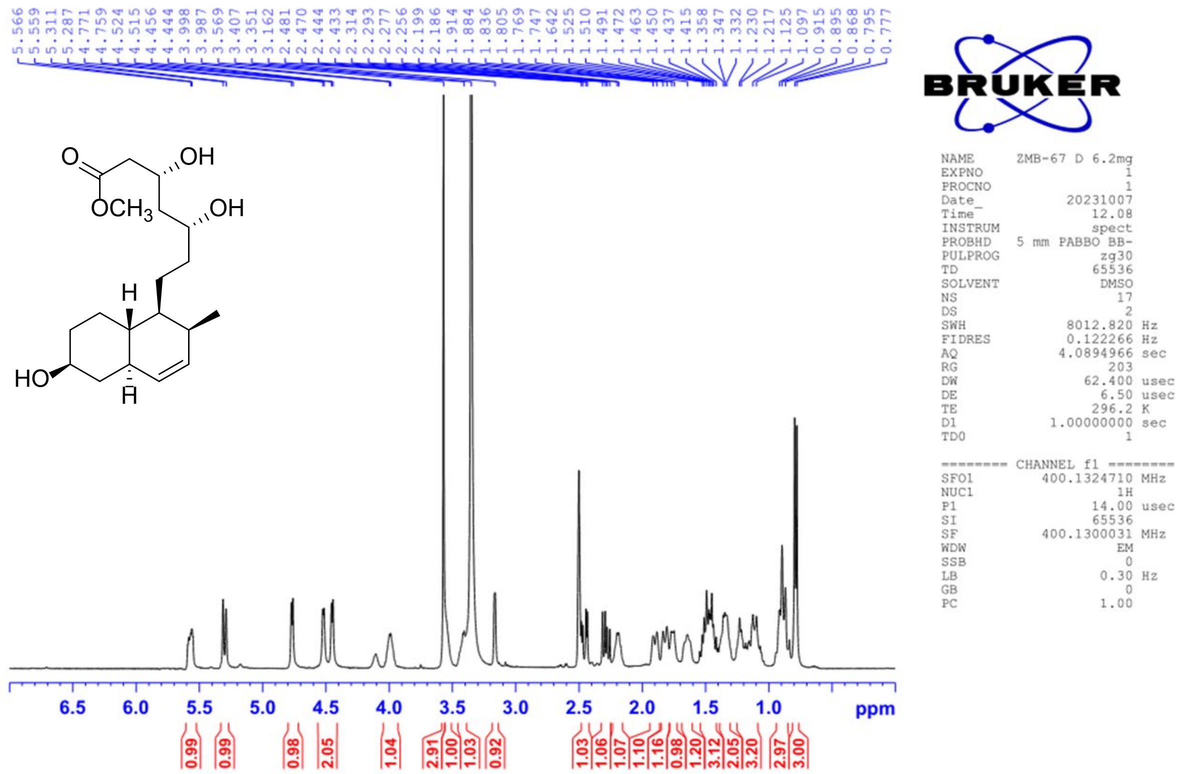

Figure S33. ^{1}H NMR (400 MHz, DMSO-d_6) spectrum of **5**.

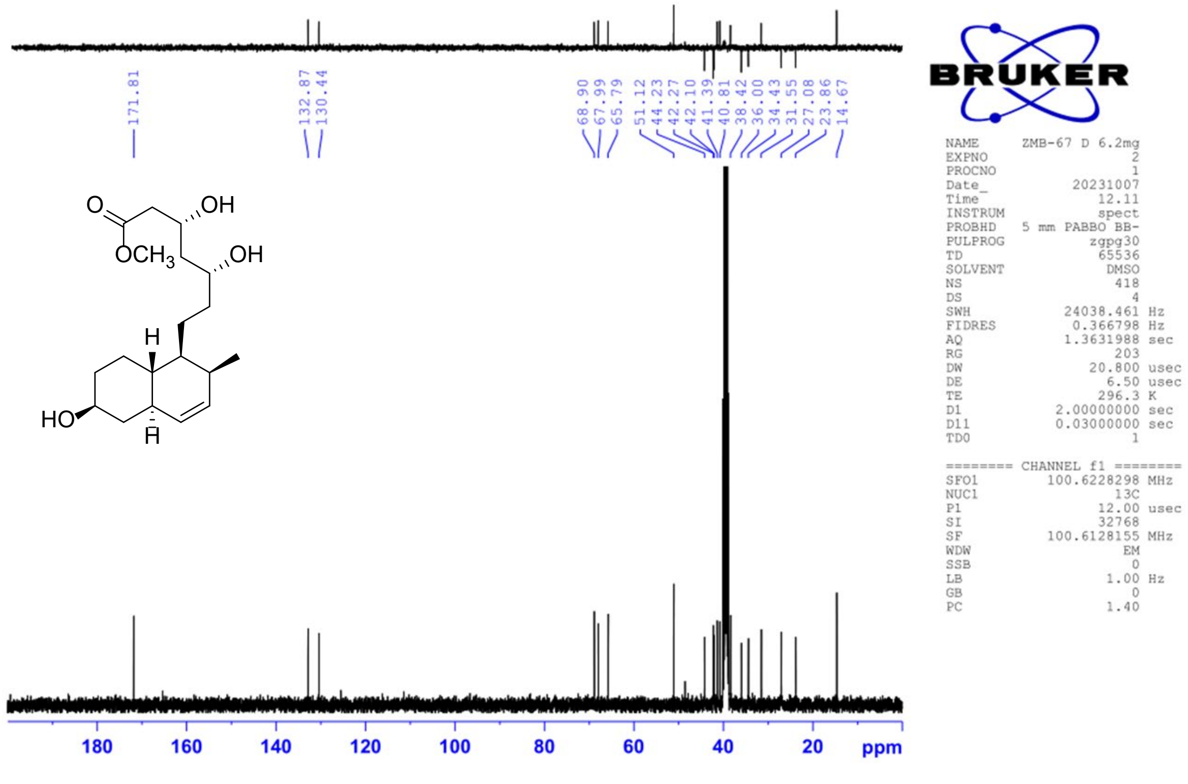

Figure S34. ^{13}C NMR (100 MHz, DMSO-d_6) spectrum of **5**.

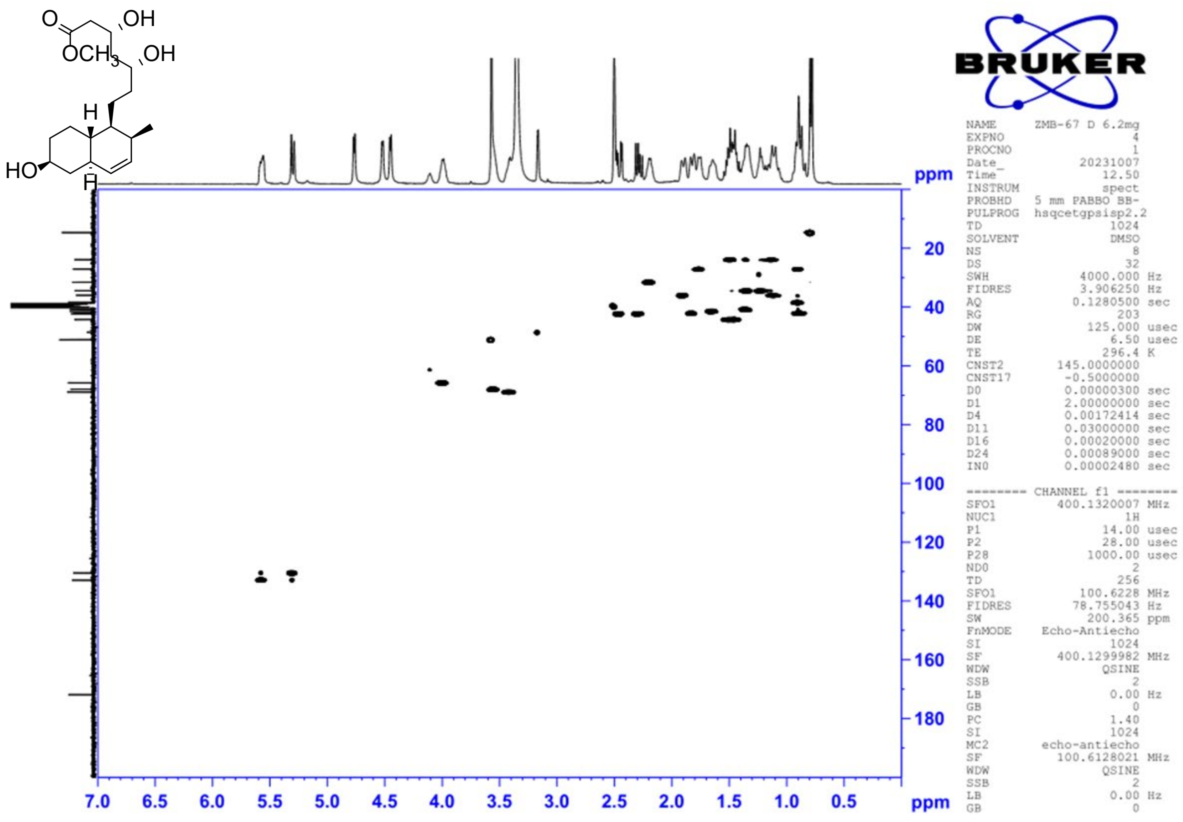

Figure S35. 2D HSQC (DMSO-d_6) spectrum of **5**.

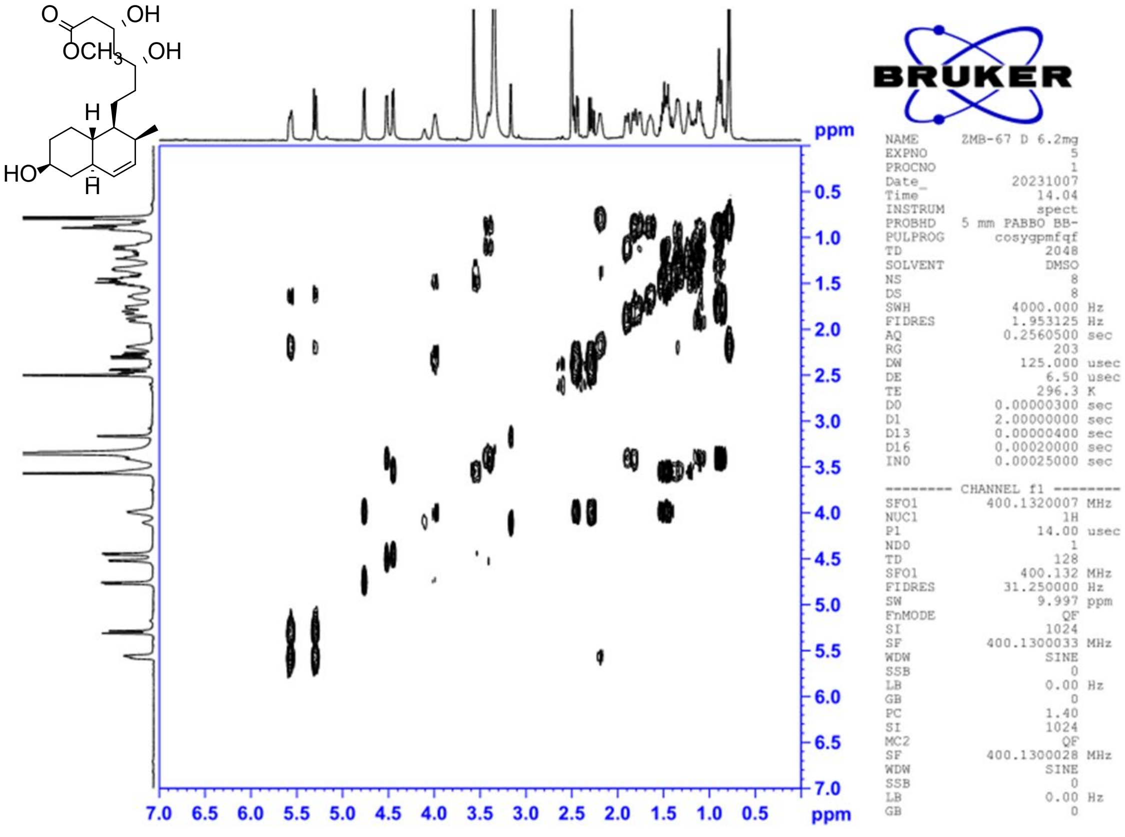

Figure S36. 2D COSY (DMSO-d_6) spectrum of **5**.

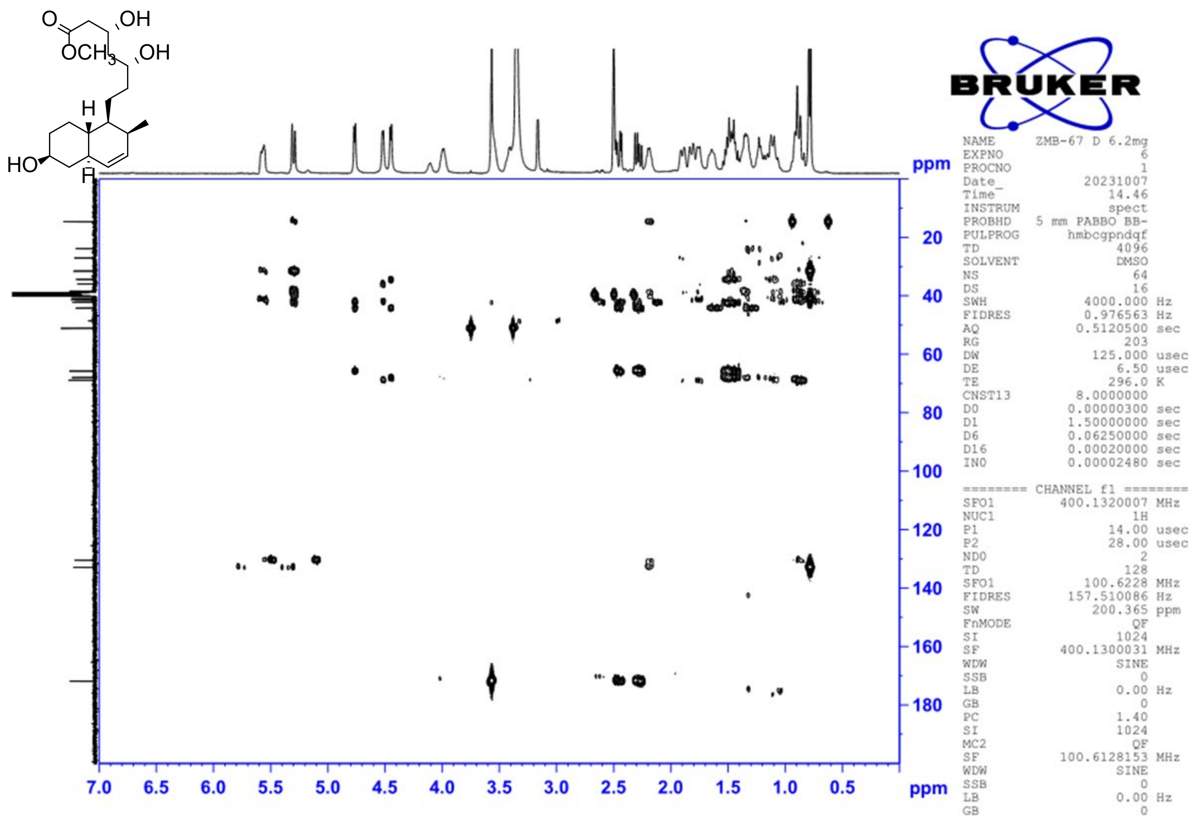

Figure S37. 2D HMBC (DMSO-d_6) spectrum of **5**.

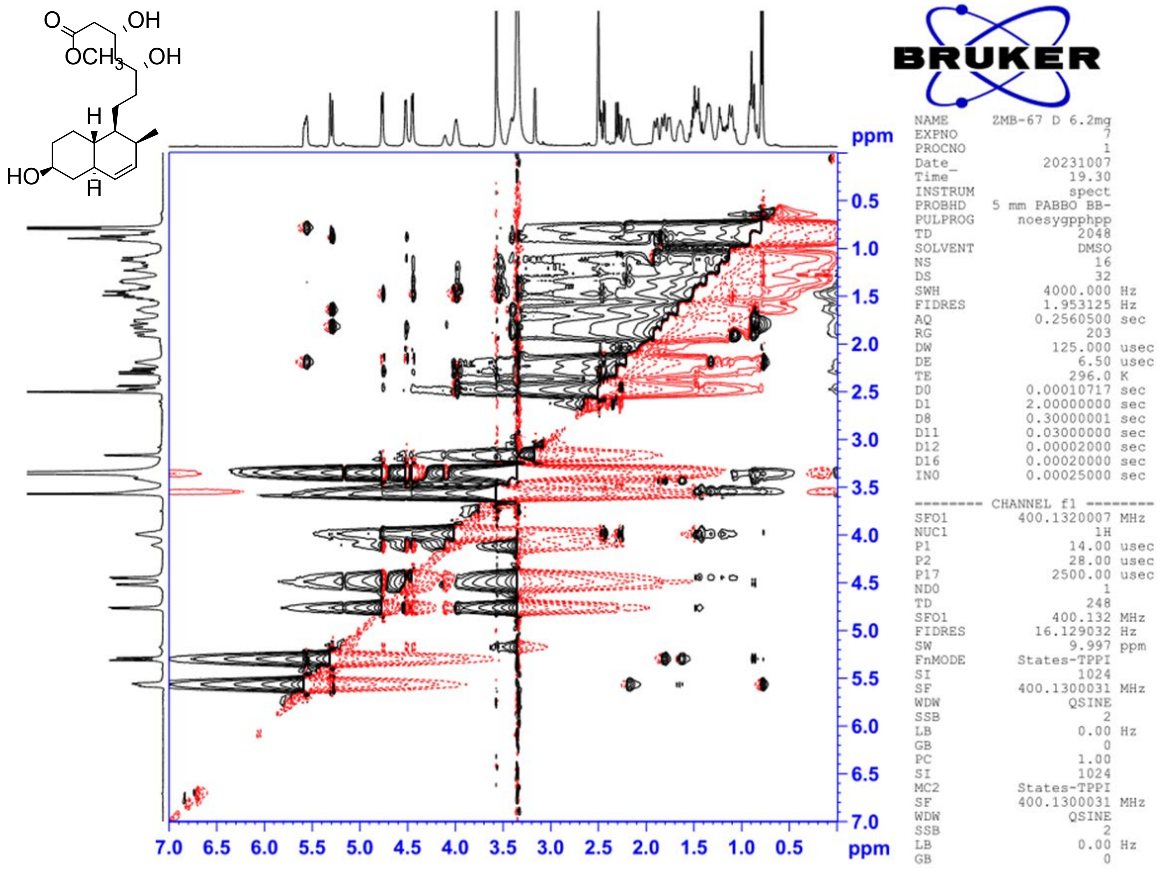

Figure S38. 2D NOESY (DMSO-d_6) spectrum of **5**.

Elemental Composition Report

Single Mass Analysis

Tolerance = 40.0 mDa / DBE: min = -1.5, max = 50.0
Element prediction: Off
Number of isotope peaks used for i-FIT = 3

Monoisotopic Mass, Even Electron Ions
17 formula(e) evaluated with 2 results within limits (up to 50 best isotopic matches for each mass)
Elements Used:
C: 0-19 H: 0-32 O: 0-7 Na: 0-1
ZMB-67 122 (0.475) Cm (86:162)
1: TOF MS ES+

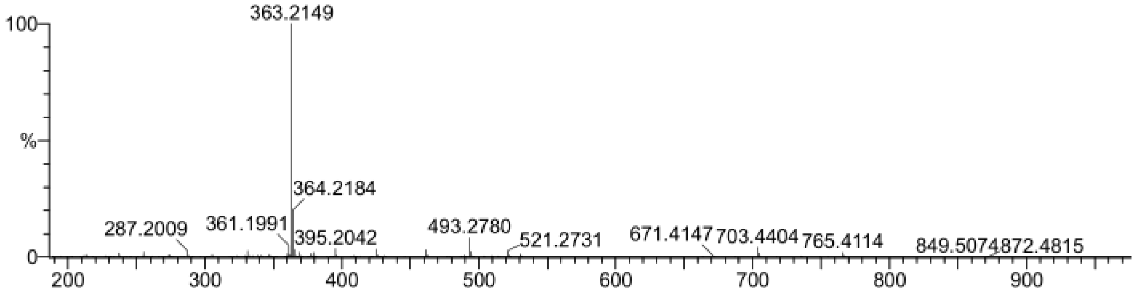

Minimum:				-1.5				
Maximum:		40.0	10.0	50.0				
Mass	Calc. Mass	mDa	PPM	DBE	i-FIT	Norm	Conf(%)	Formula
363.2149	363.1784	36.5	100.5	4.5	2277.2	0.006	99.43	C18 H28 O6 Na
	363.2147	0.2	0.6	3.5	2282.3	5.163	0.57	C19 H32 O5 Na

Figure S39. HRMS spectrum and measurement of **5**.

Anton Paar GmbH
Anton Paar Strasse 10
8054 Graz
Austria

Anton Paar

Anton Paar Polarimeter - Measurement(s)

MCP 100
Software Version: 1.50.4098.87
Serial Number: 99032784

Unique Id 2307

- Sample Name: ZMB-67
- Date: 12/08/2023 - 02:53 PM
- Username: Administrator
- Sample State: Ok

- Measurement Mode: Specific Rotation
- Measurement Result: 44.000 °
- Concentration: 0.100 g/100ml
- Optical Rotation: 0.044 °
- Set Temperature: 25.0 °C
- Temperature: 25.0 °C

Figure S40. Specific rotation data of compound **5**.

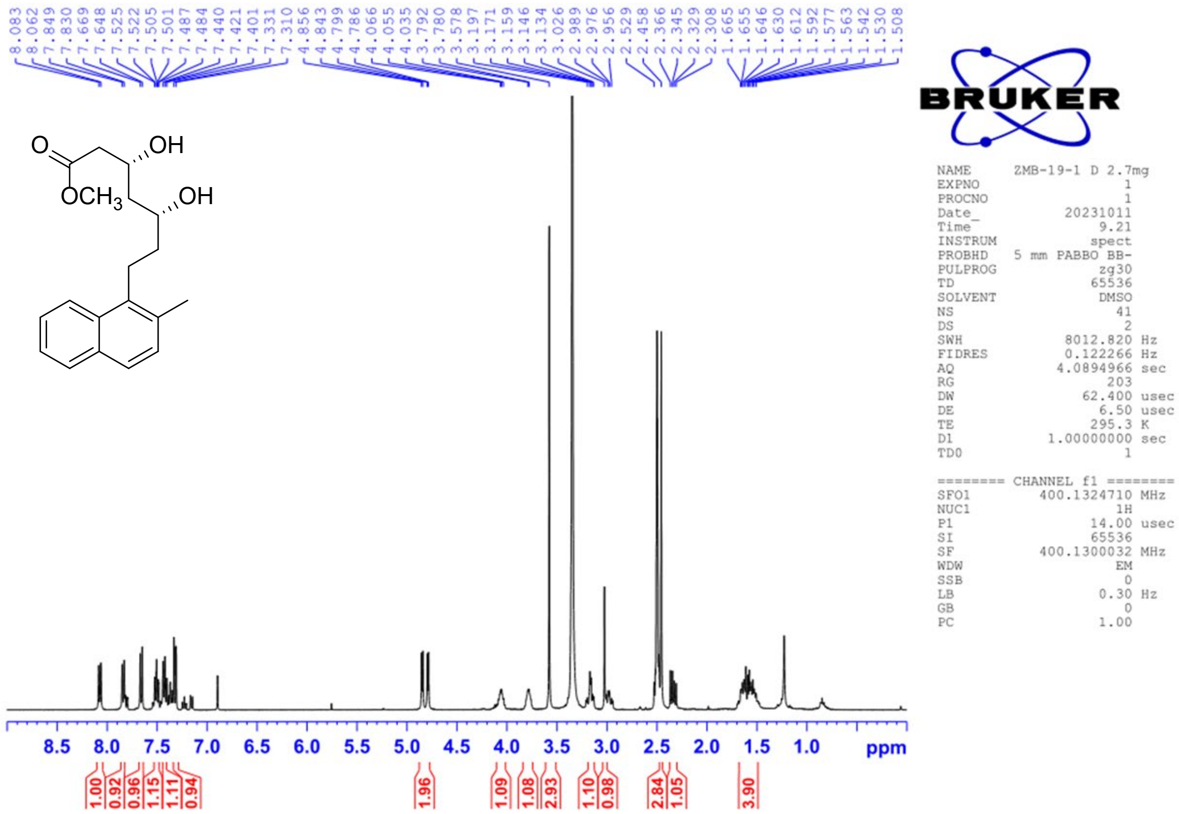

Figure S41. ^{1}H NMR (400 MHz, DMSO-d_6) spectrum of **6**.

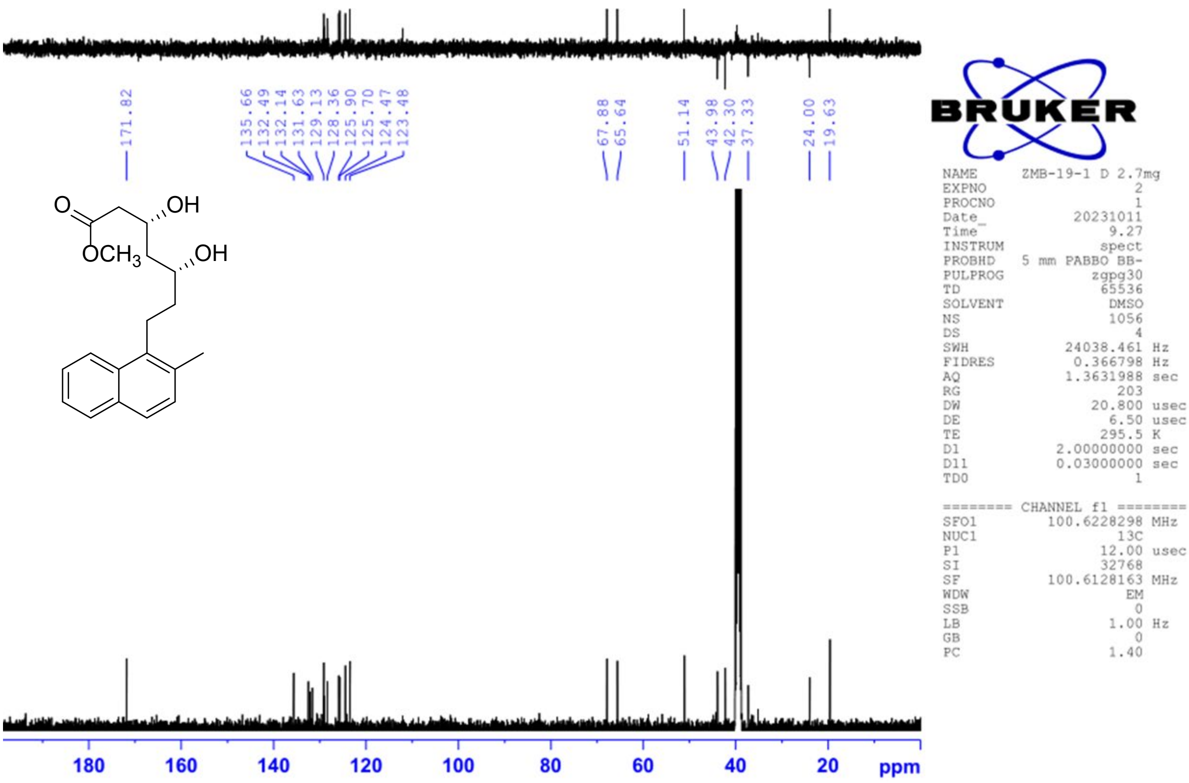

Figure S42. ^{13}C NMR (100 MHz, DMSO-d_6) spectrum of **6**.

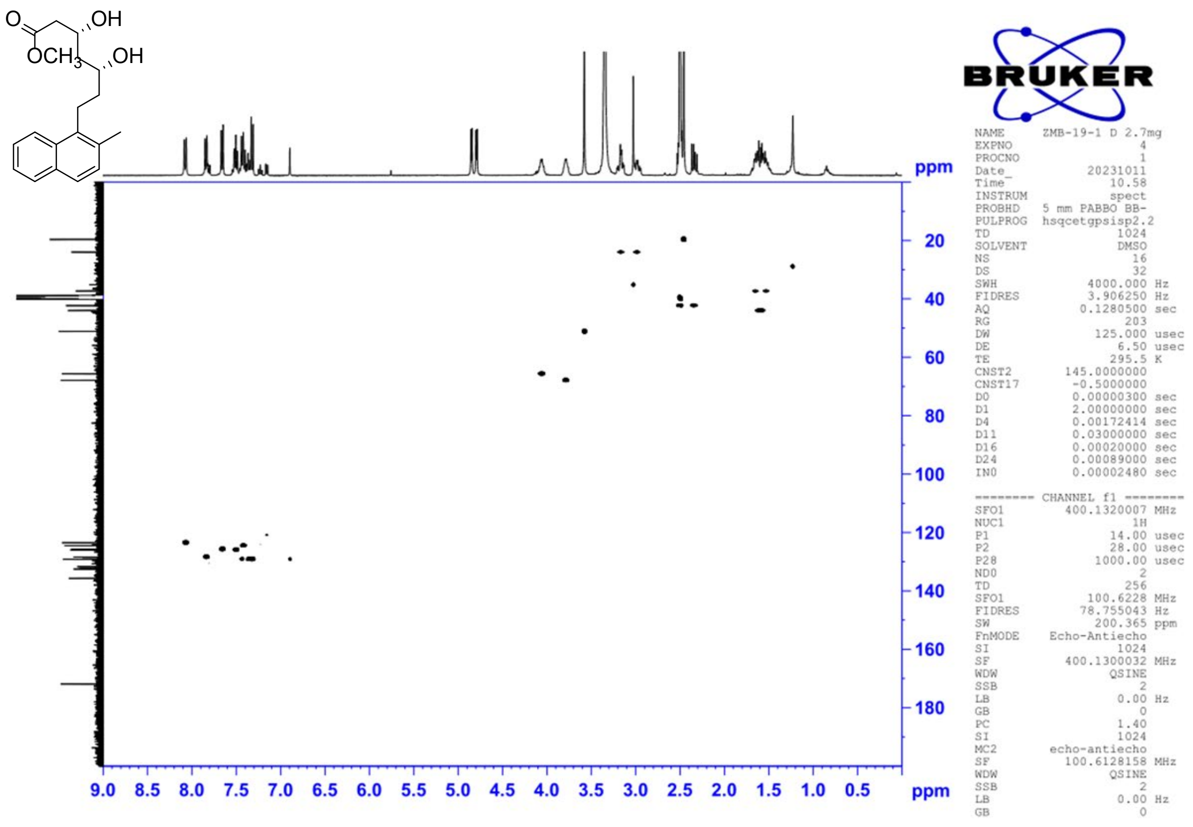

Figure S43. 2D HSQC (DMSO-d_6) spectrum of **6**.

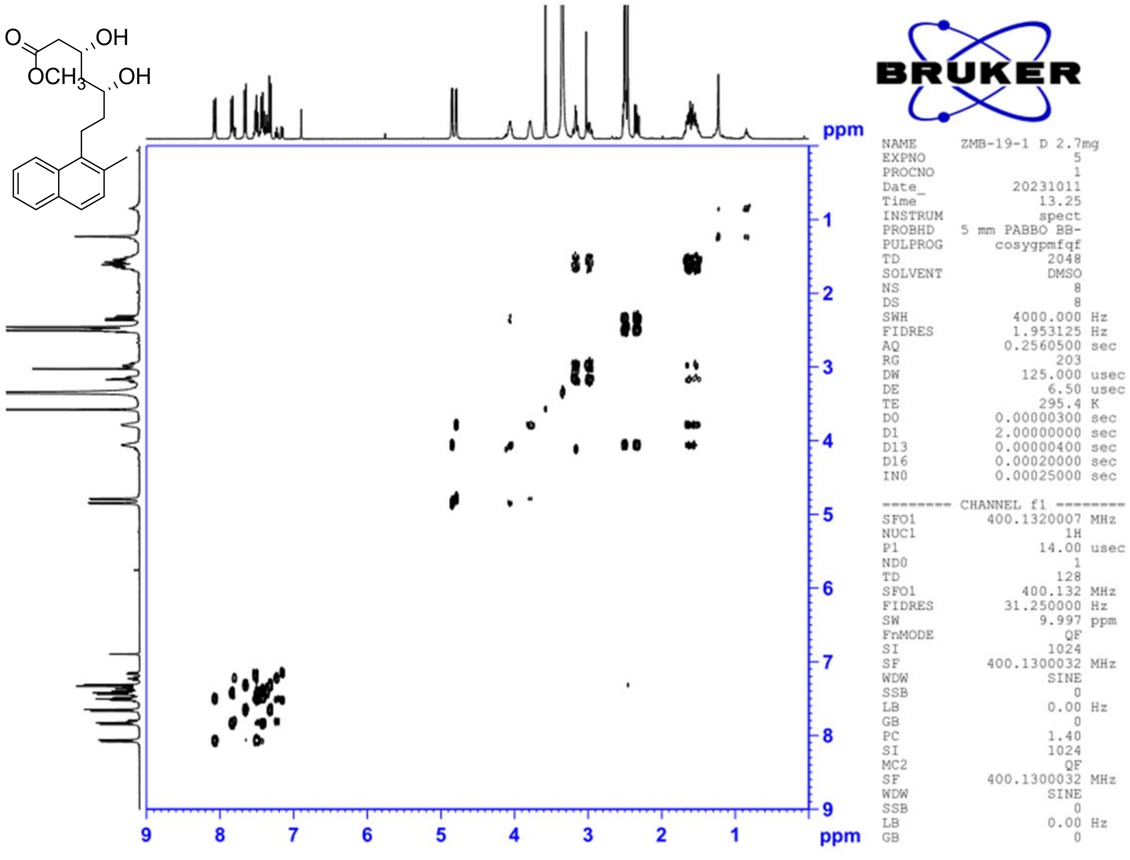

Figure S44. 2D COSY (DMSO-d_6) spectrum of **6**.

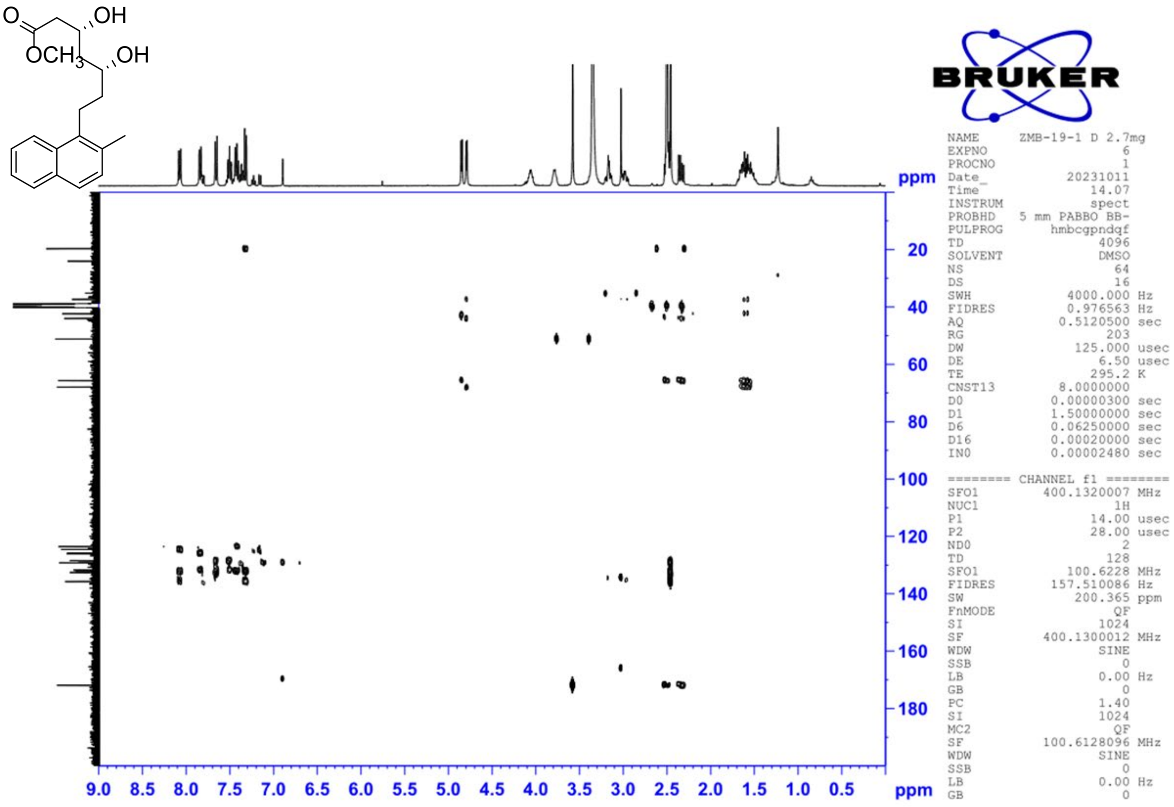

Figure S45. 2D HMBC (DMSO-d_6) spectrum of **6**.

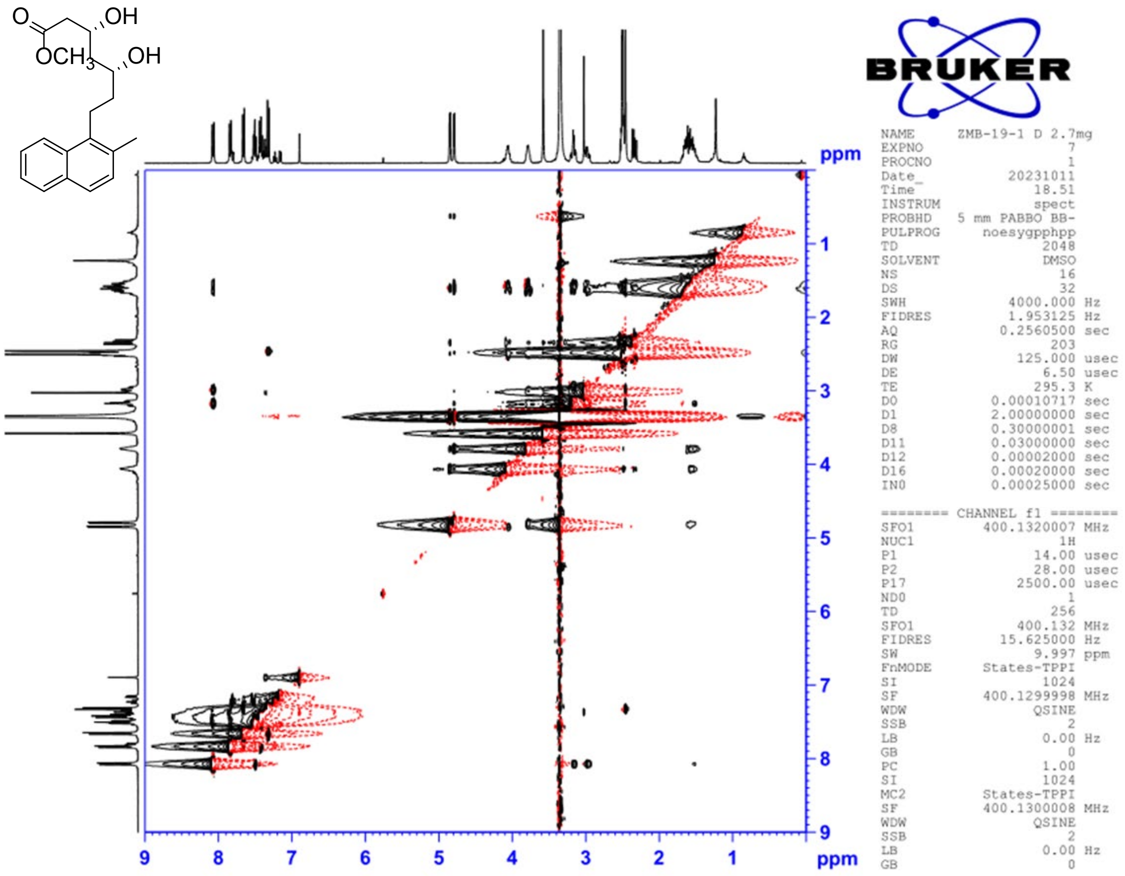

Figure S46. 2D NOESY (DMSO-d_6) spectrum of **6**.

Elemental Composition Report

Single Mass Analysis
Tolerance = 40.0 mDa / DBE: min = -1.5, max = 50.0
Element prediction: Off
Number of isotope peaks used for i-FIT = 3

Monoisotopic Mass, Even Electron Ions
292 formula(e) evaluated with 31 results within limits (up to 50 best isotopic matches for each mass)
Elements Used:
C: 0-34 H: 0-50 N: 0-5 O: 0-5 Na: 0-1
ZMB-19-1 118 (0.461) Cm (62:153)
1: TOF MS ES+

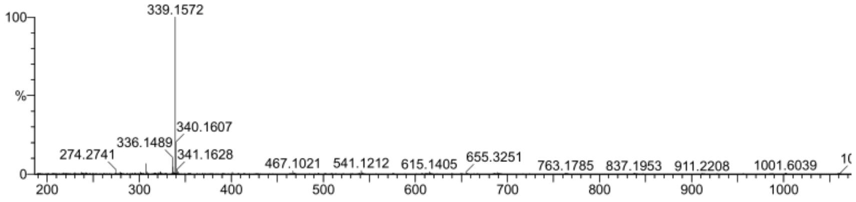

		mDa	PPM	DBE				
Minimum:				-1.5				
Maximum:		40.0	10.0	50.0				

Mass	Calc. Mass	mDa	PPM	DBE	i-FIT	Norm	Conf(%)	Formula
339.1572	339.1572	0.0	0.0	7.5	2402.0	0.283	75.32	C19 H24 O4 Na
	339.1685	-11.3	-33.3	7.5	2404.0	2.252	10.51	C18 H24 N2 O3 Na
	339.1457	11.5	33.9	11.5	2404.6	2.832	5.89	C18 H19 N4 O3
	339.1208	36.4	107.3	8.5	2404.8	3.103	4.49	C18 H20 O5 Na
	339.1920	-34.8	-102.6	5.5	2406.1	4.347	1.29	C17 H27 N2 O5
	339.1345	22.7	66.9	11.5	2406.6	4.838	0.79	C19 H19 N2 O4
	339.1321	25.1	74.0	8.5	2406.6	4.903	0.74	C17 H20 N2 O4 Na
	339.1797	-22.5	-66.3	7.5	2407.8	6.080	0.23	C17 H24 N4 O2 Na
	339.1433	13.9	41.0	8.5	2408.0	6.301	0.18	C16 H20 N4 O3 Na
	339.1709	-13.7	-40.4	10.5	2408.4	6.668	0.13	C20 H23 N2 O3
	339.1821	-24.9	-73.4	10.5	2408.7	6.994	0.09	C19 H23 N4 O2
	339.1668	-9.6	-28.3	6.5	2408.8	7.056	0.09	C15 H23 N4 O5
	339.1936	-36.4	-107.3	6.5	2409.1	7.316	0.07	C20 H28 O3 Na
	339.1596	-2.4	-7.1	10.5	2409.2	7.464	0.06	C21 H23 O4
	339.1232	34.0	100.2	11.5	2409.6	7.851	0.04	C20 H19 O5
	339.1586	-1.4	-4.1	12.5	2410.0	8.237	0.03	C20 H20 N4 Na
	339.1222	35.0	103.2	13.5	2410.2	8.475	0.02	C19 H16 N4 O Na
	339.1473	9.9	29.2	12.5	2410.9	9.157	0.01	C21 H20 N2 O Na
	339.1896	-32.4	-95.5	2.5	2411.3	9.542	0.01	C15 H28 N2 O5 Na
	339.1644	-7.2	-21.2	3.5	2411.8	10.061	0.00	C13 H24 N4 O5 Na
	339.1610	-3.8	-11.2	15.5	2412.0	10.230	0.00	C22 H19 N4
	339.1361	21.1	62.2	12.5	2412.2	10.494	0.00	C22 H20 O2 Na
	339.1246	32.6	96.1	16.5	2412.5	10.780	0.00	C21 H15 N4 O
	339.1497	7.5	22.1	15.5	2412.8	11.088	0.00	C23 H19 N2 O
	339.1960	-38.8	-114.4	9.5	2412.9	11.143	0.00	C22 H27 O3
	339.1837	-26.5	-78.1	11.5	2412.9	11.200	0.00	C22 H24 N2 Na
	339.1725	-15.3	-45.1	11.5	2413.1	11.365	0.00	C23 H24 O Na

Figure S47. HRMS spectrum and measurement of **6**.

Anton Paar GmbH
Anton Paar Strasse 10
8054 Graz
Austria

Anton Paar

Anton Paar Polarimeter - Measurement(s)

MCP 100
Software Version: 1.50.4098.87
Serial Number: 99032784

Unique Id 2283

- Sample Name: ZMB-19-1
- Date: 12/08/2023 - 12:55 PM
- Username: Administrator
- Sample State: Ok

- Measurement Mode: Specific Rotation
- Measurement Result: 18.000 °
- Concentration: 0.100 g/100ml
- Optical Rotation: 0.018 °
- Set Temperature: 25.0 °C
- Temperature: 25.0 °C

Figure S48. Specific rotation data of compound **6**.

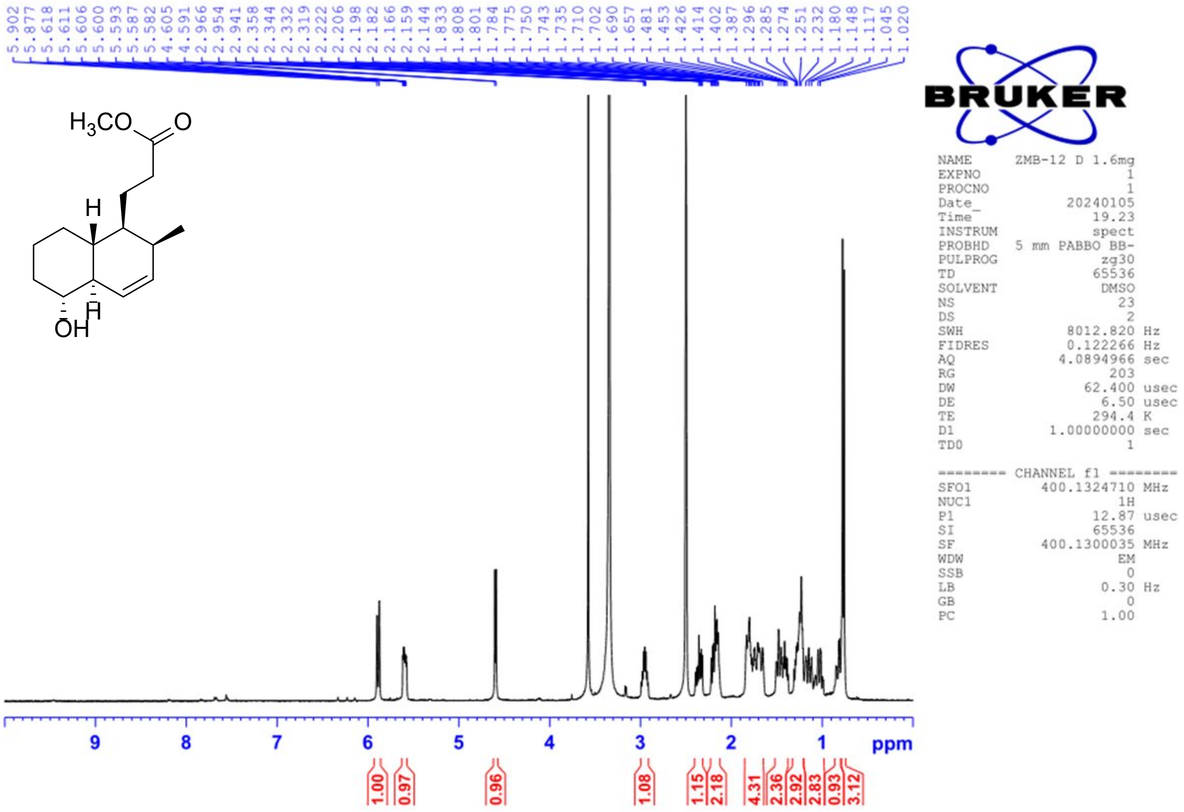

Figure S49. ^{1}H NMR (400 MHz, DMSO-d_6) spectrum of **7**.

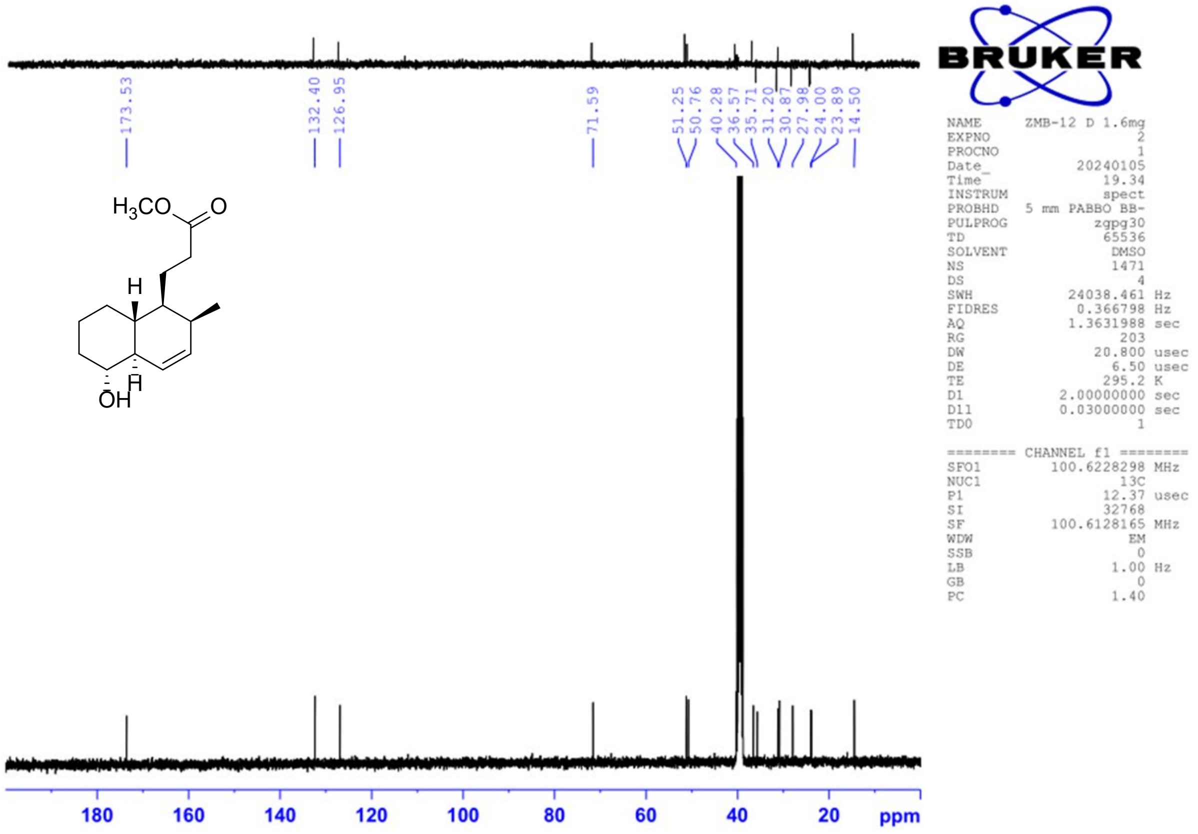

Figure S50. ^{13}C NMR (100 MHz, DMSO-d_6) spectrum of **7**.

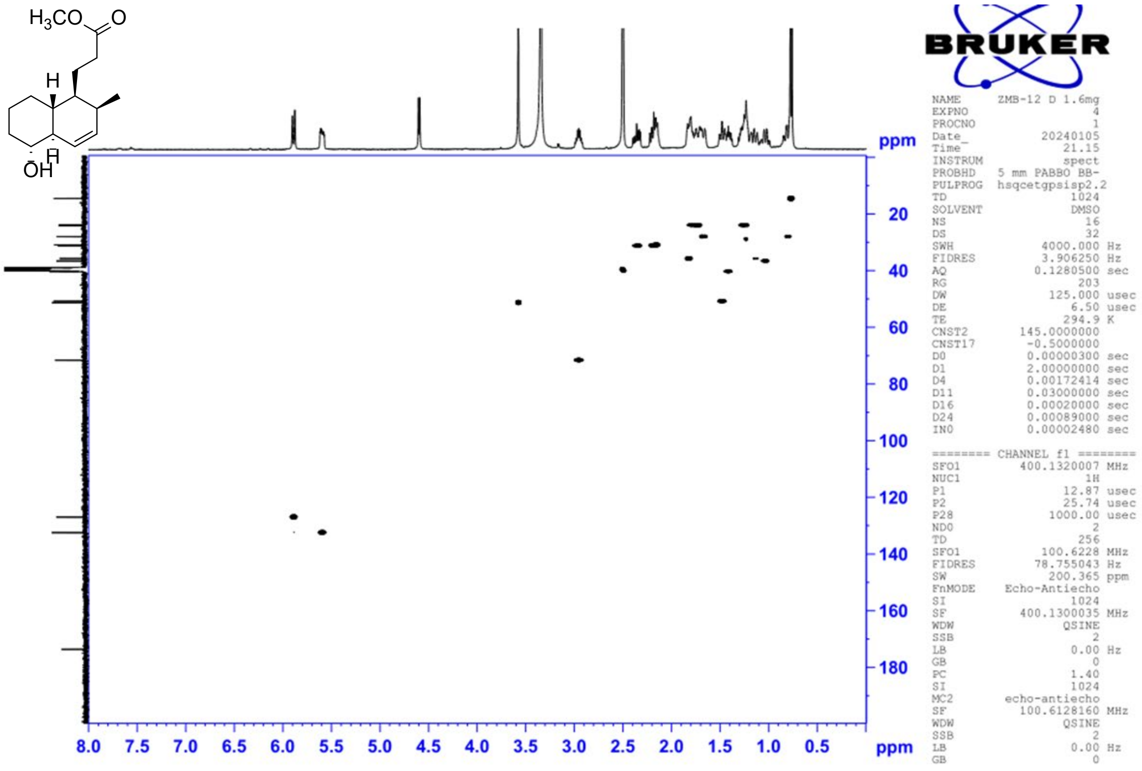

Figure S51. 2D HSQC (DMSO-d_6) spectrum of **7**.

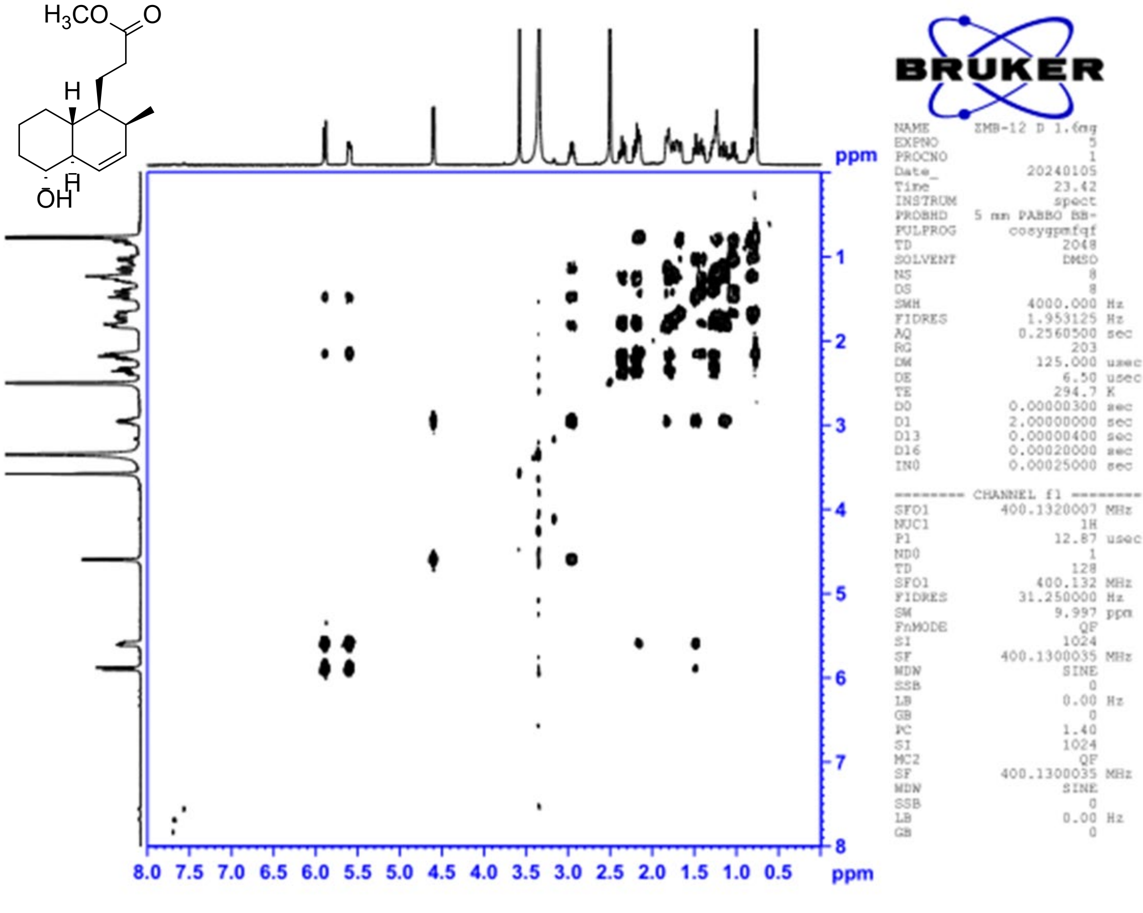

Figure S52. 2D COSY (DMSO-d_6) spectrum of **7**.

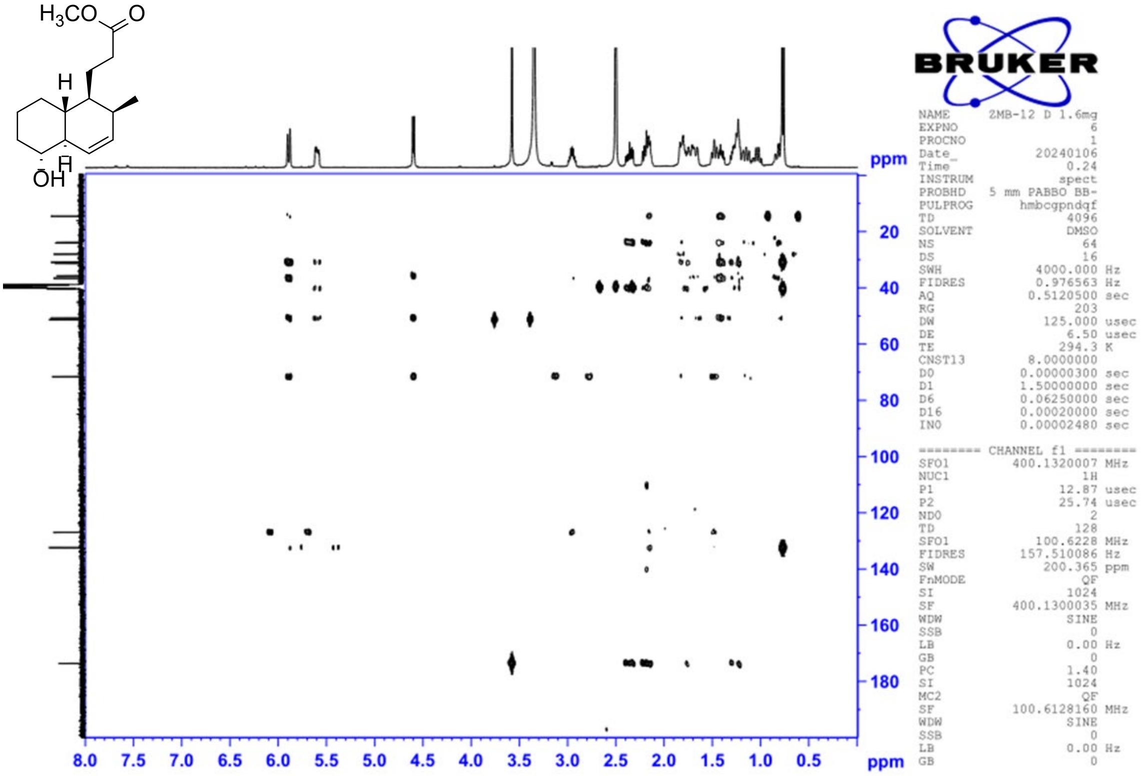

Figure S53. 2D HMBC (DMSO-d_6) spectrum of **7**.

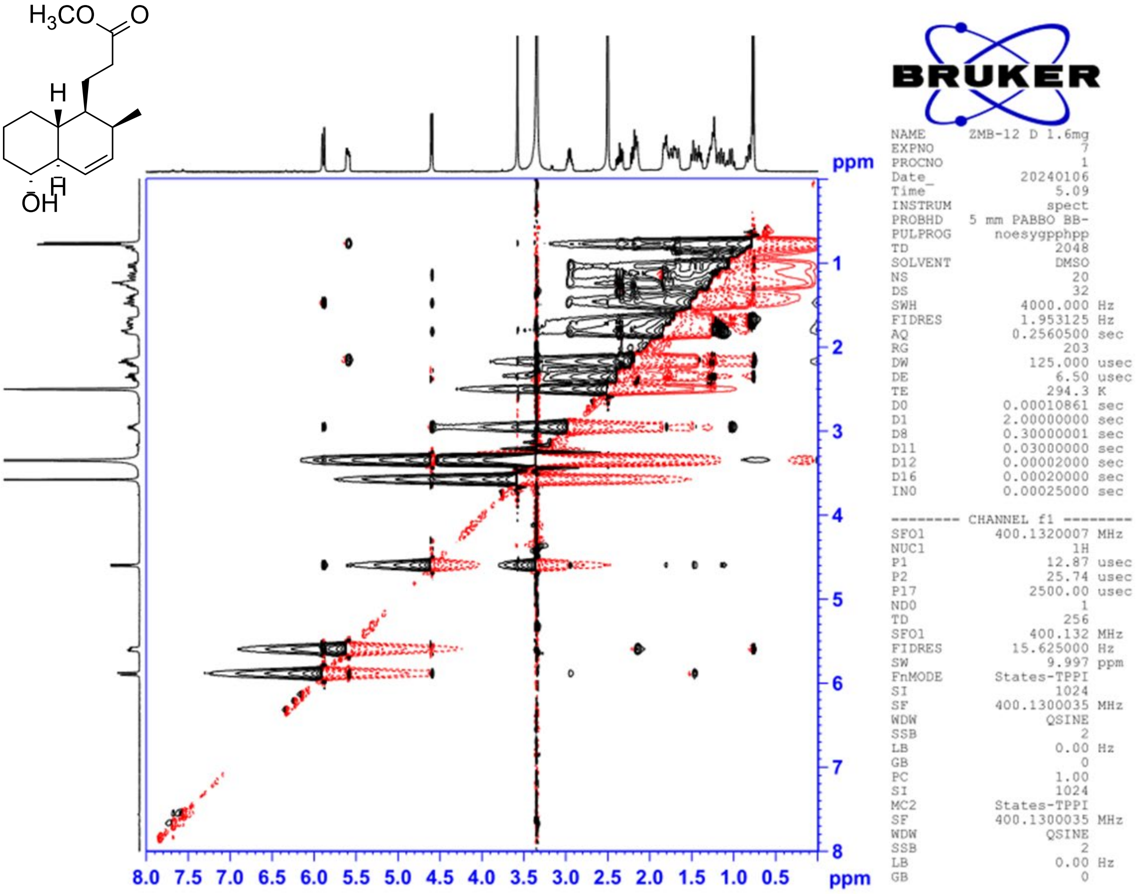

Figure S54. 2D NOESY (DMSO-d_6) spectrum of **7**.

Anton Paar GmbH
Anton Paar Strasse 10
8054 Graz
Austria

Anton Paar Polarimeter - Measurement(s)

MCP 100
Software Version: 1.50.4098.87
Serial Number: 99032784

Unique Id 2193

- Sample Name: ZMB-12
- Date: 09/12/2023 - 09:04 AM
- Username: Administrator
- Sample State: Ok

- Measurement Mode: Specific Rotation
- Measurement Result: 62.000 °
- Concentration: 0.200 g/100ml
- Optical Rotation: 0.124 °
- Set Temperature: 25.0 °C
- Temperature: 25.0 °C

Figure S55. Specific rotation data of compound **7**.

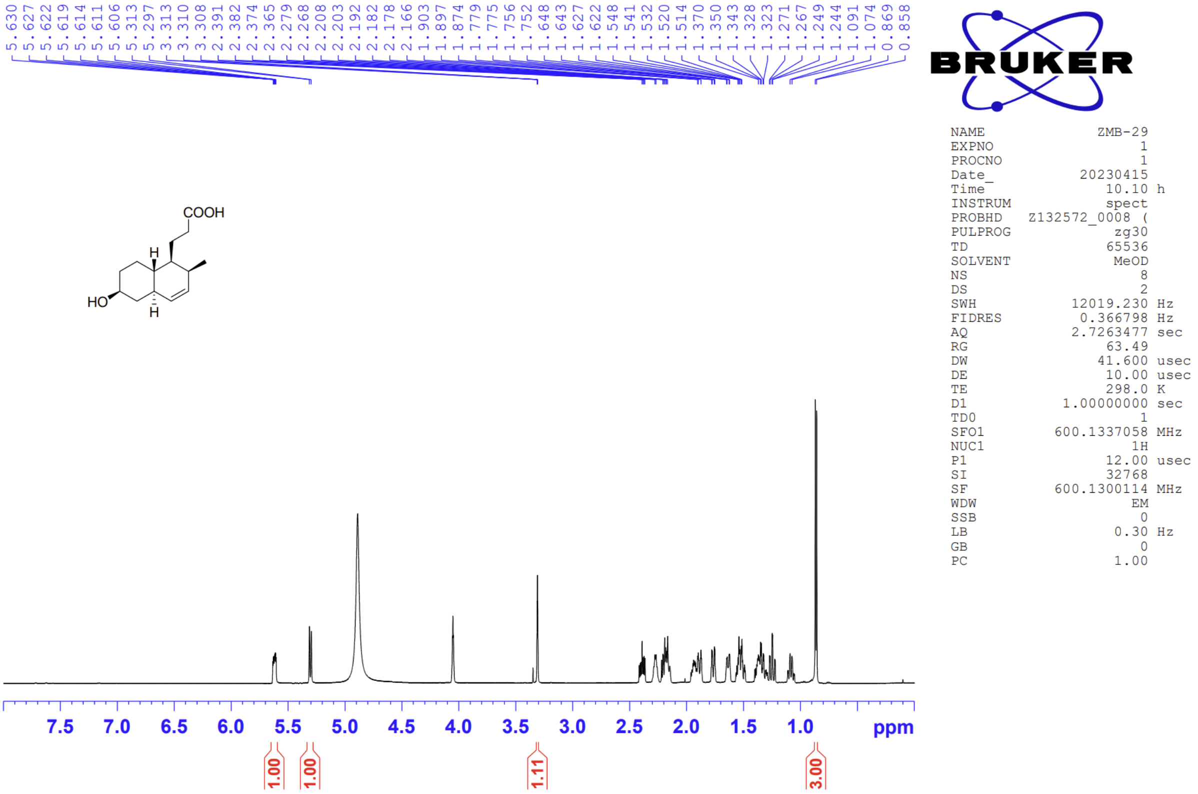

Figure S56. ^{1}H NMR (400 MHz, MeOD) spectrum of **8**.

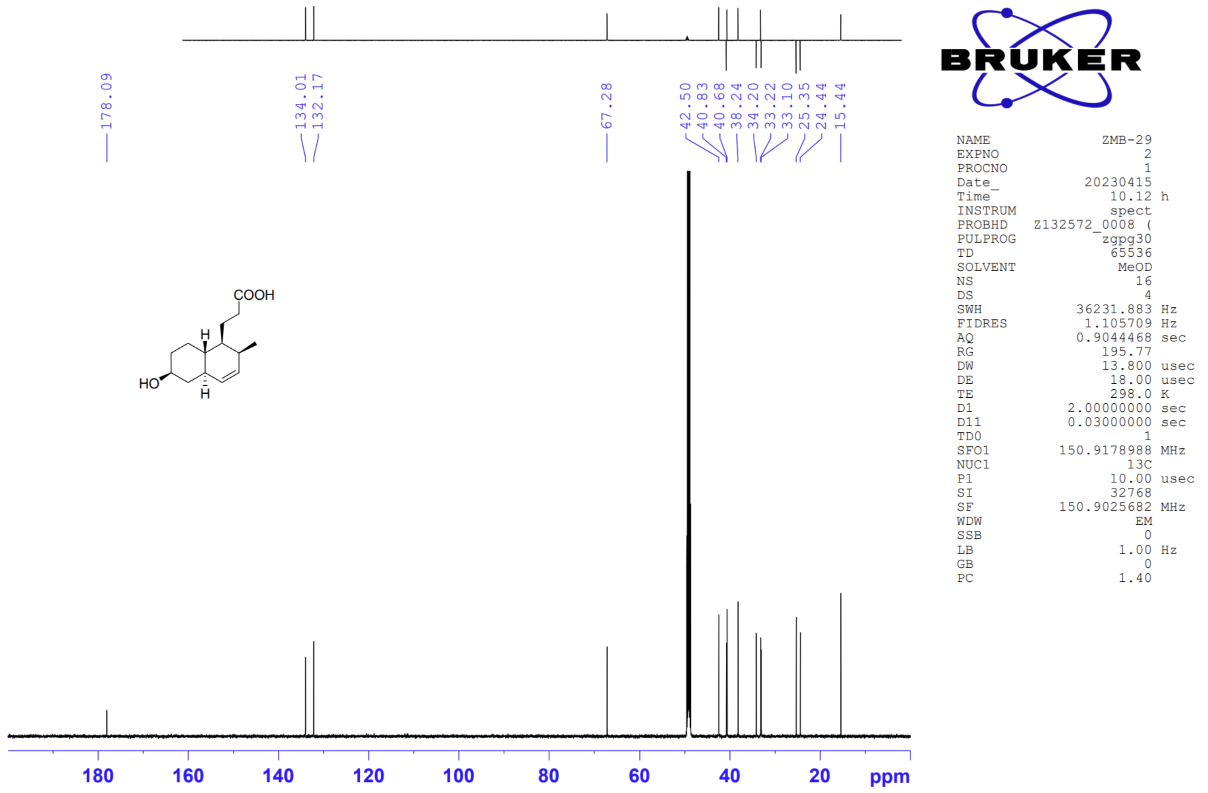

Figure S57. ^{13}C NMR (100 MHz, MeOD) spectrum of **8**.

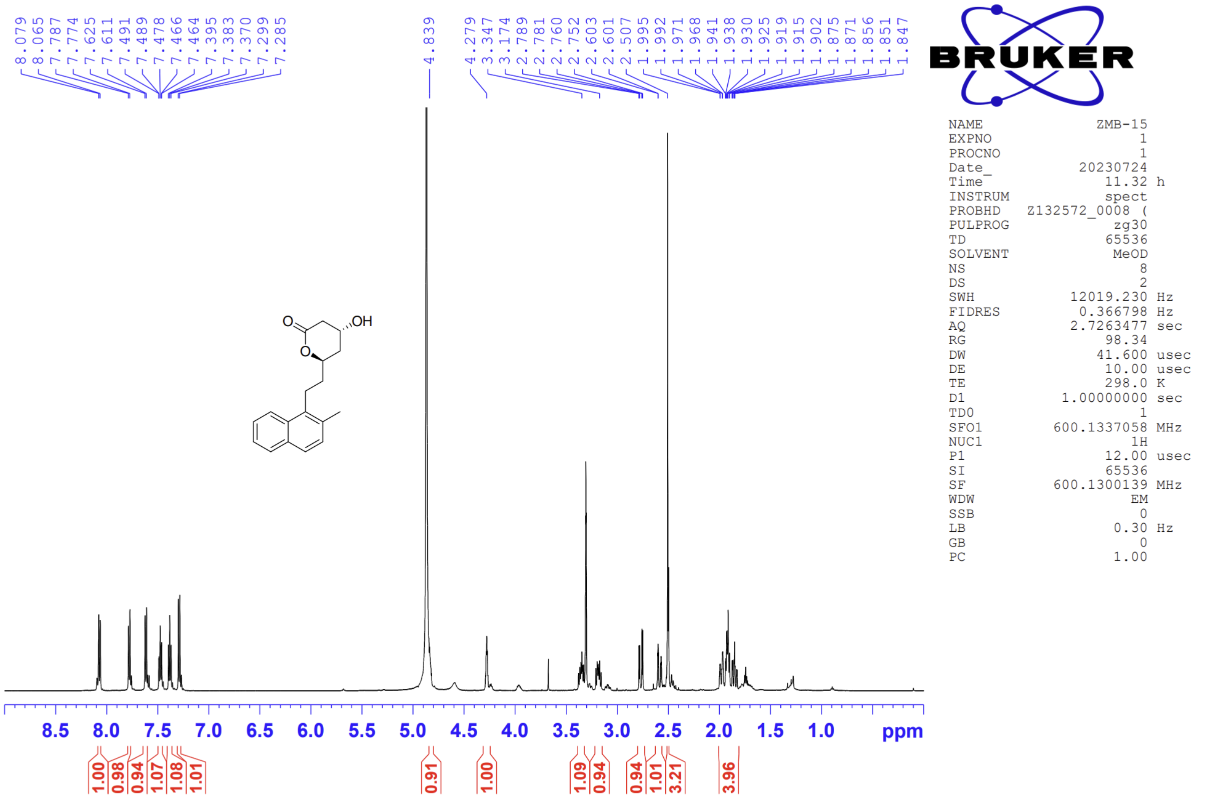

Figure S58. ^{1}H NMR (400 MHz, MeOD) spectrum of **9**.

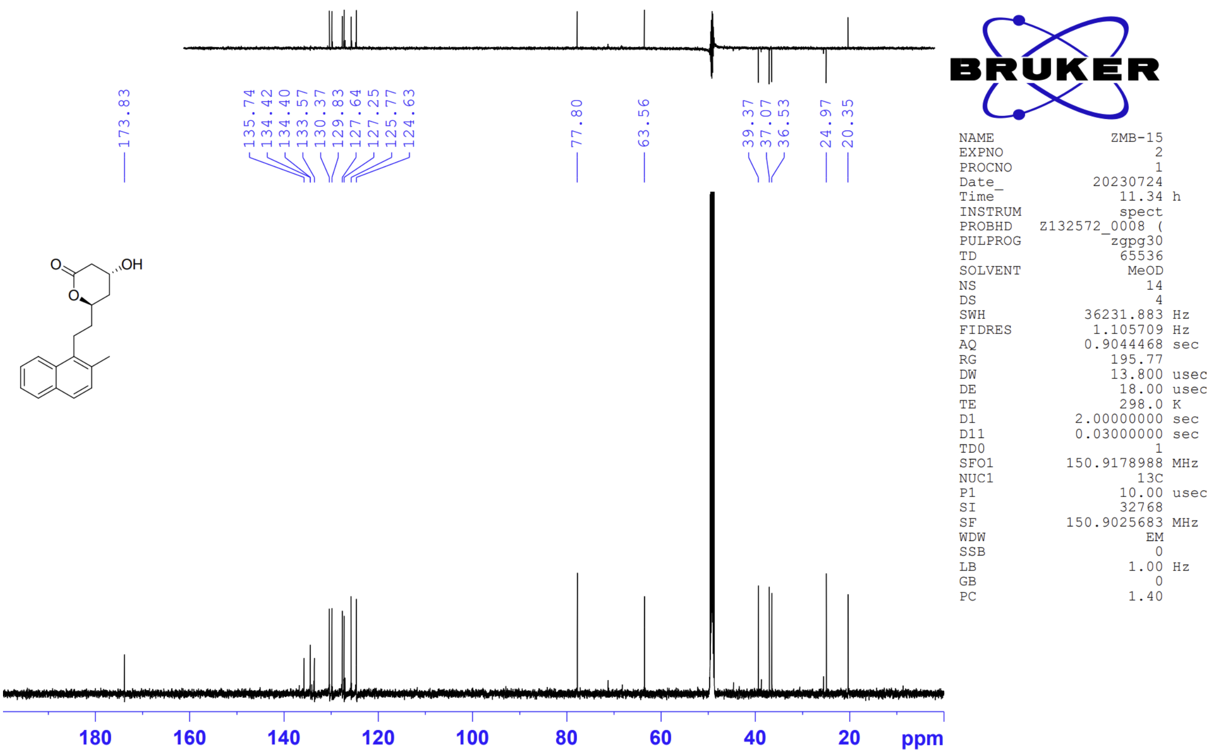

Figure S59. ^{13}C NMR (100 MHz, MeOD) spectrum of **9**.

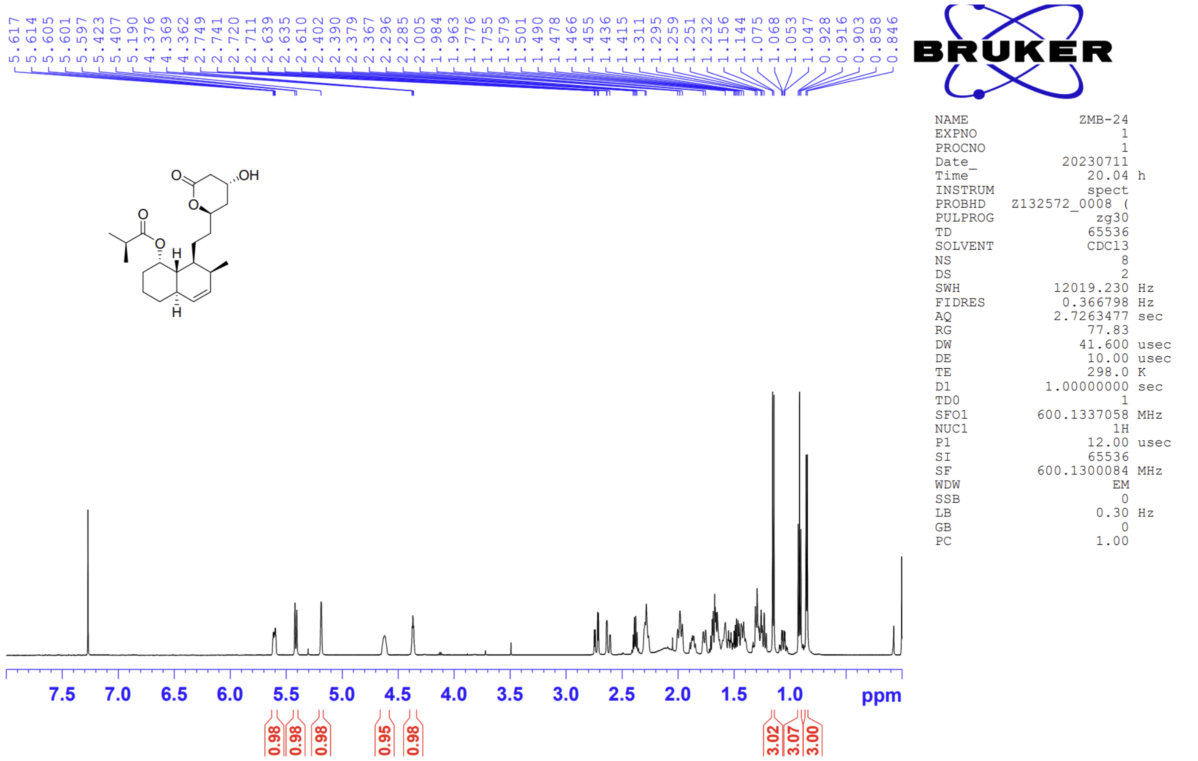

Figure S60. ^{1}H NMR (400 MHz, $CDCl_3$) spectrum of **10**.

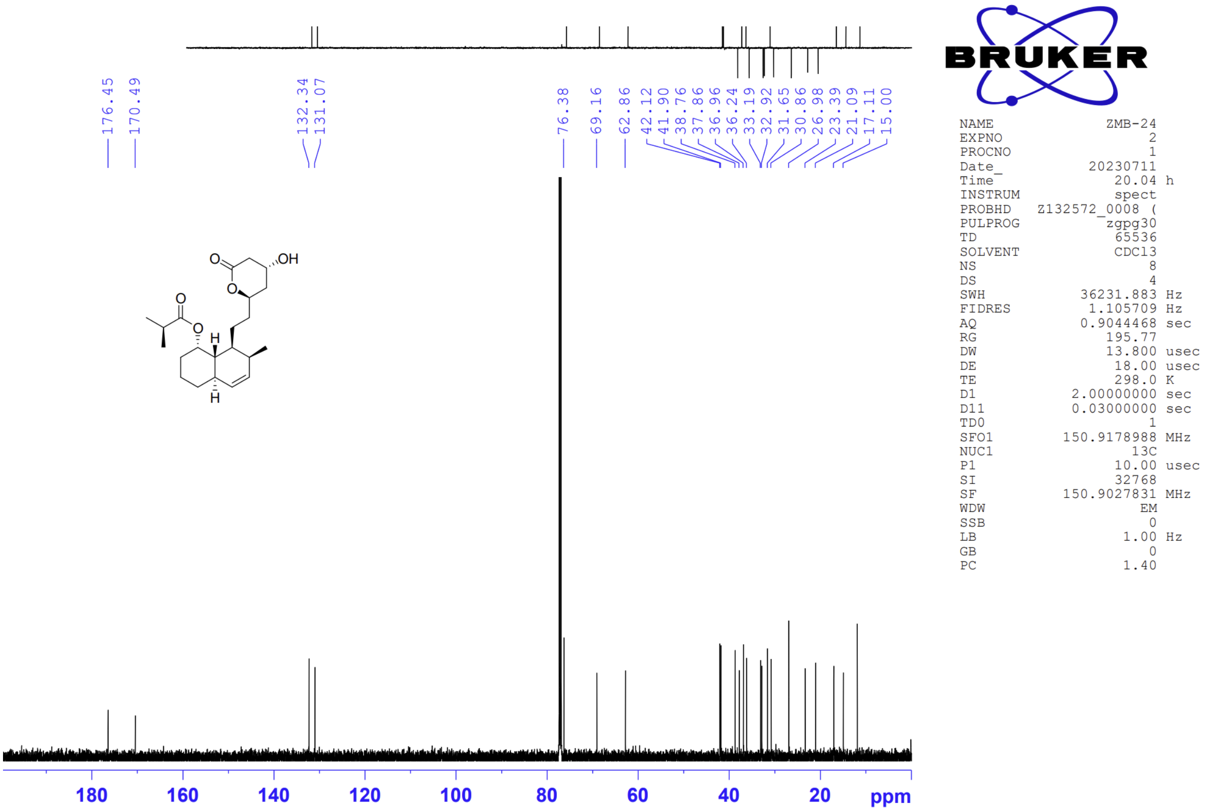

Figure S61. ^{13}C NMR (100 MHz, CDCl$_3$) spectrum of **10**.

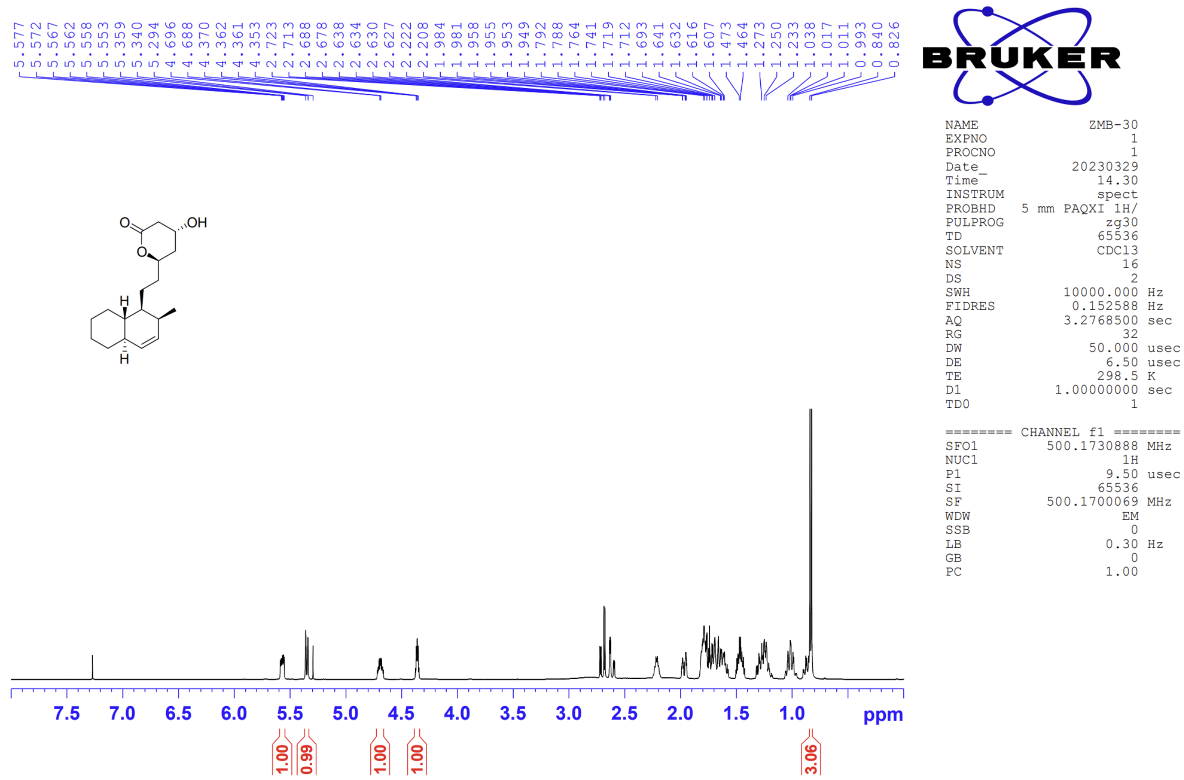

Figure S62. ^{1}H NMR (400 MHz, CDCl$_3$) spectrum of **11**.

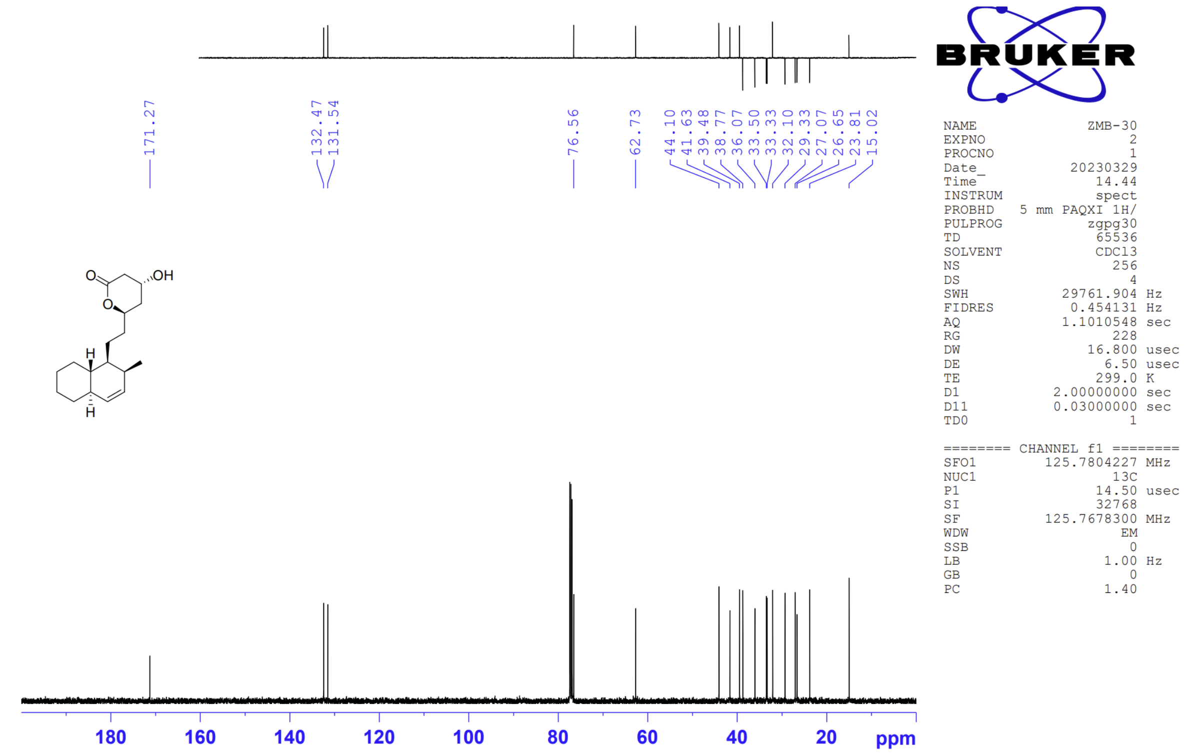

Figure S63. ^{13}C NMR (100 MHz, $CDCl_3$) spectrum of **11**.

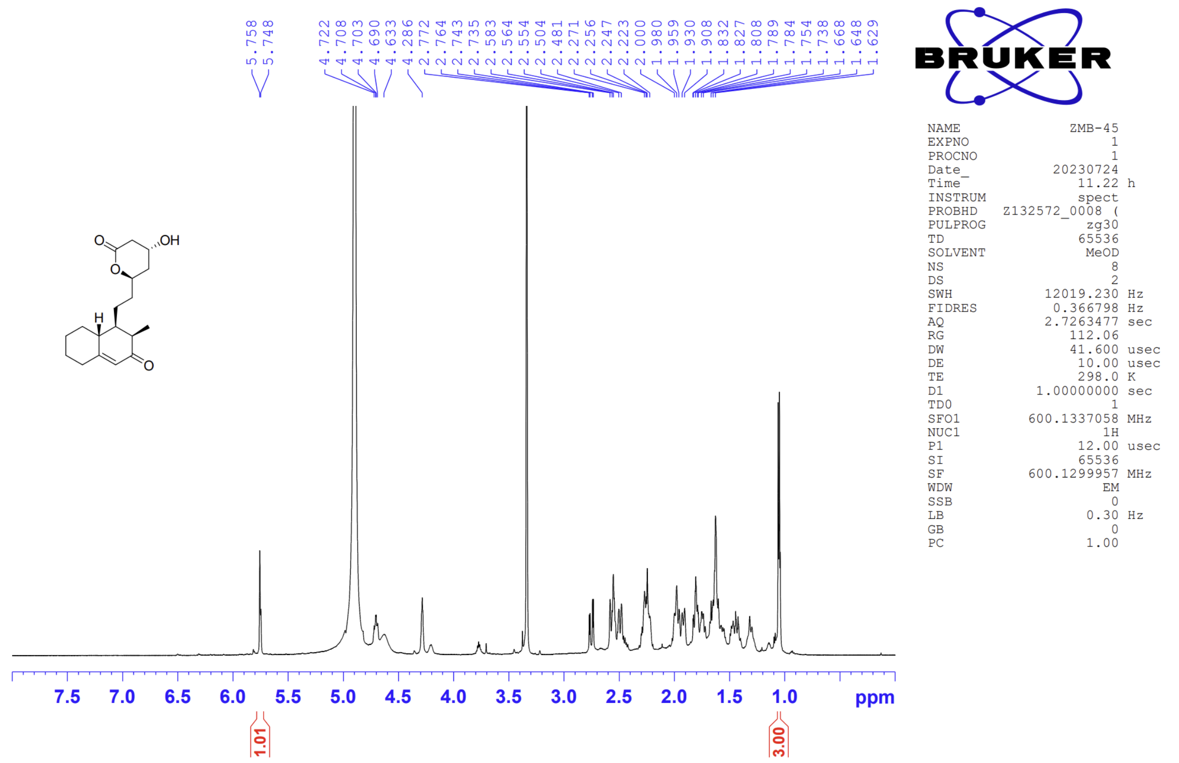

Figure S64. ^{1}H NMR (400 MHz, MeOD) spectrum of **12**.

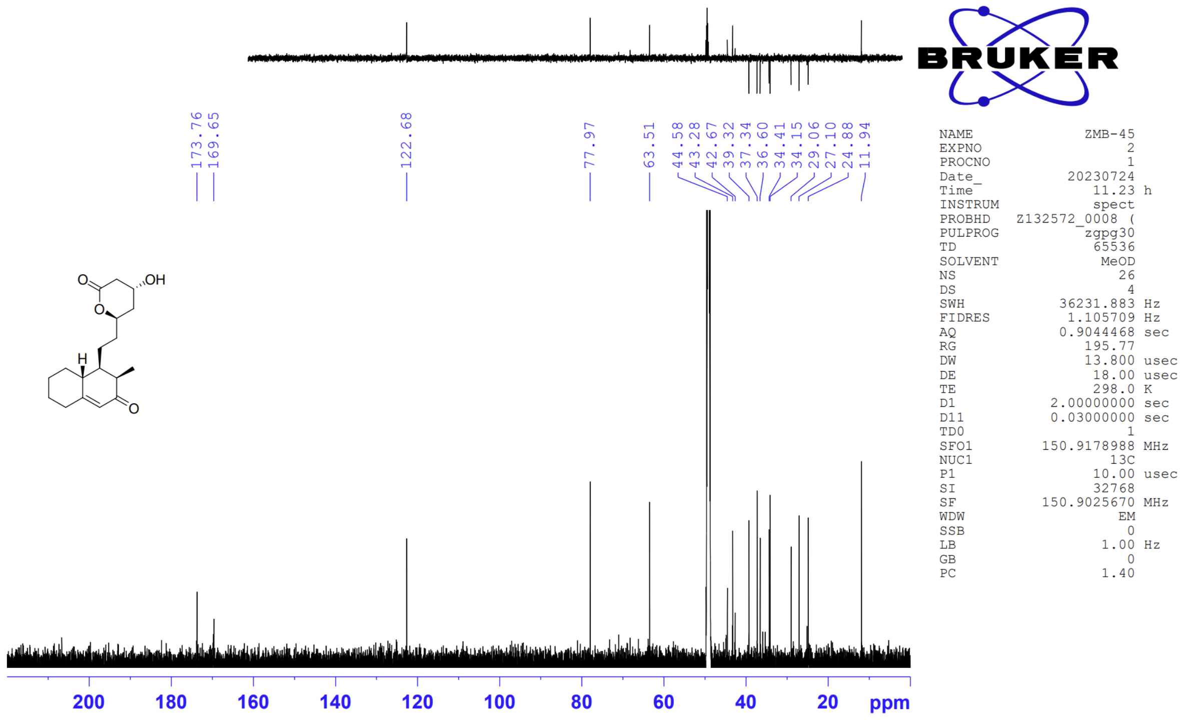

Figure S65. ^{13}C NMR (100 MHz, MeOD) spectrum of **12**.

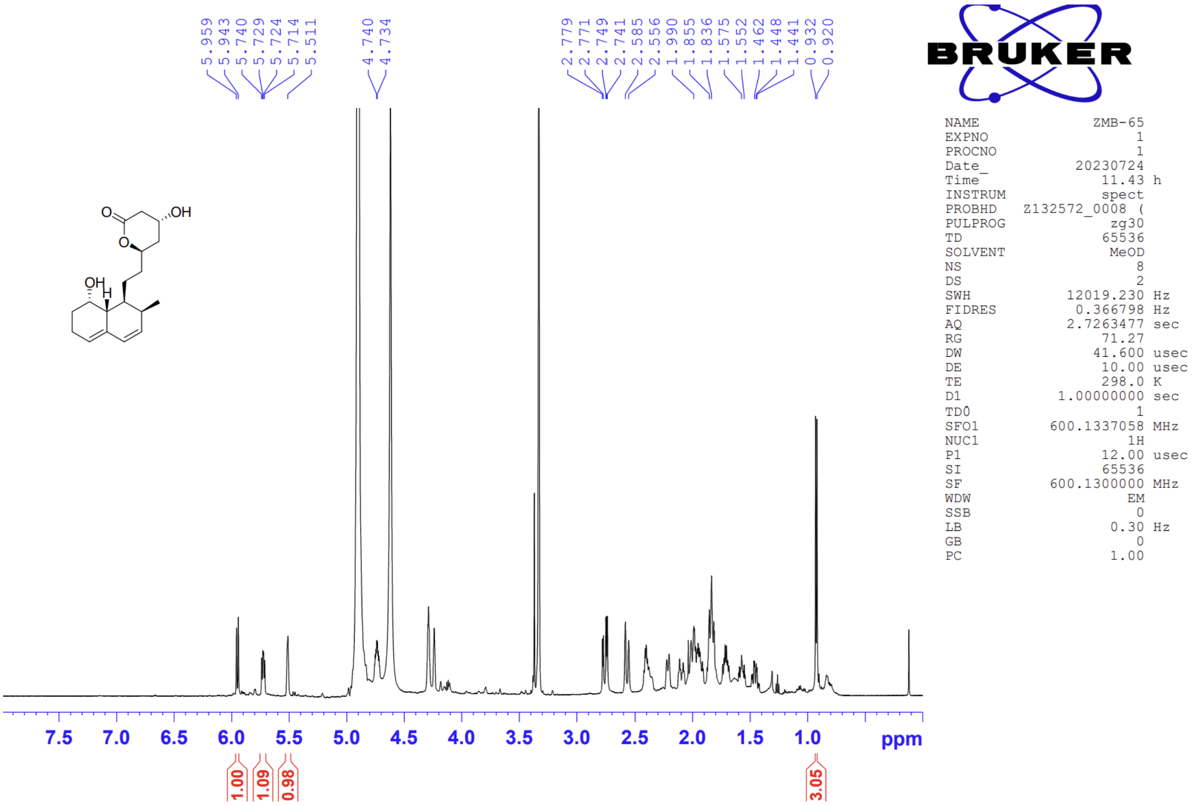

Figure S66. ^{1}H NMR (400 MHz, MeOD) spectrum of **13**.

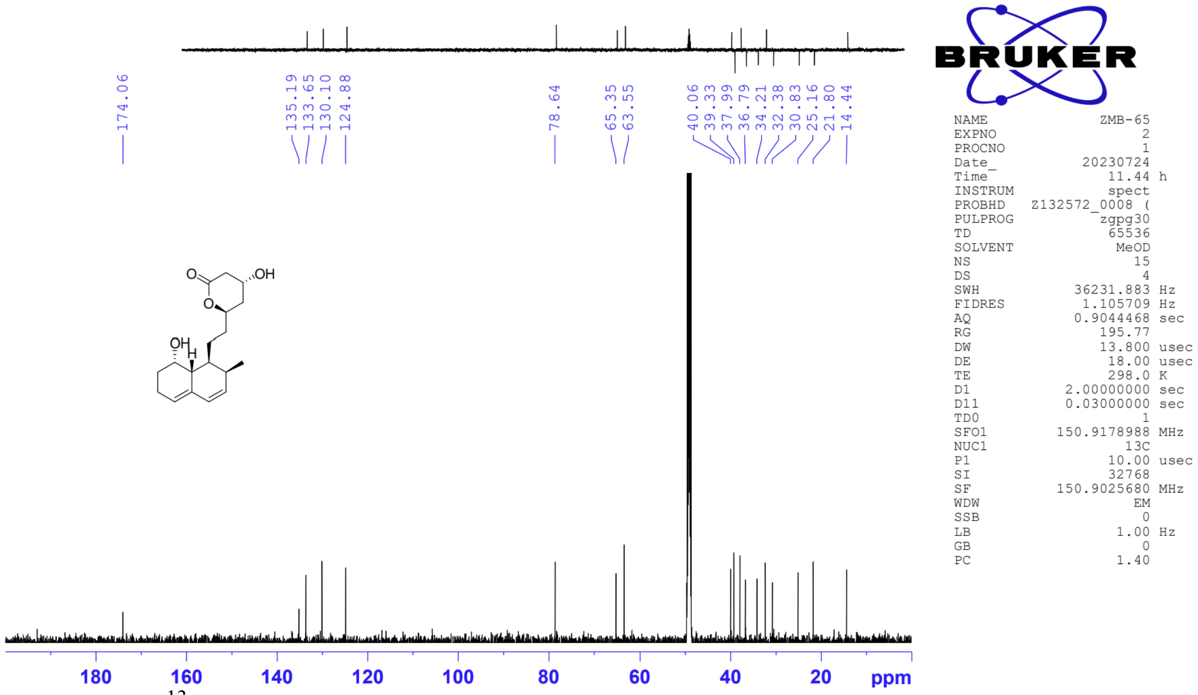

Figure S67. ^{13}C NMR (100 MHz, MeOD) spectrum of **13**.

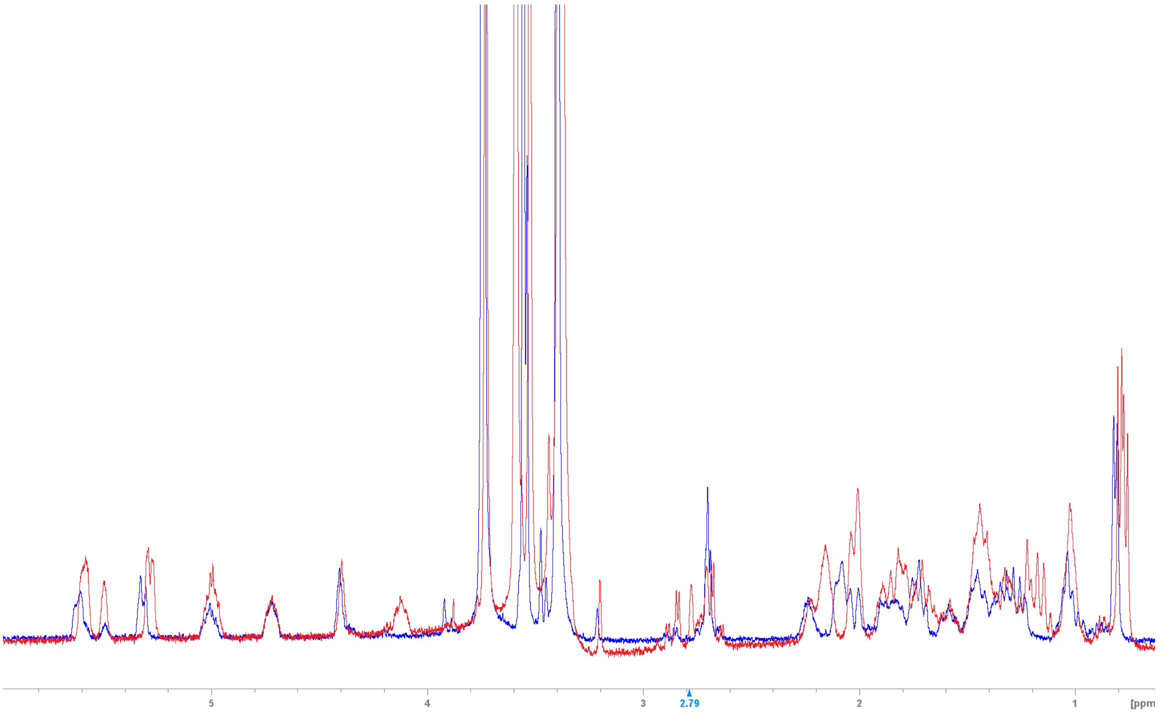

Figure S68. Stacked ^{1}H NMR spectra of 6,13-di-*S*-Mosher ester (red) and 6,13-di-*R*-Mosher ester of compound **1**.

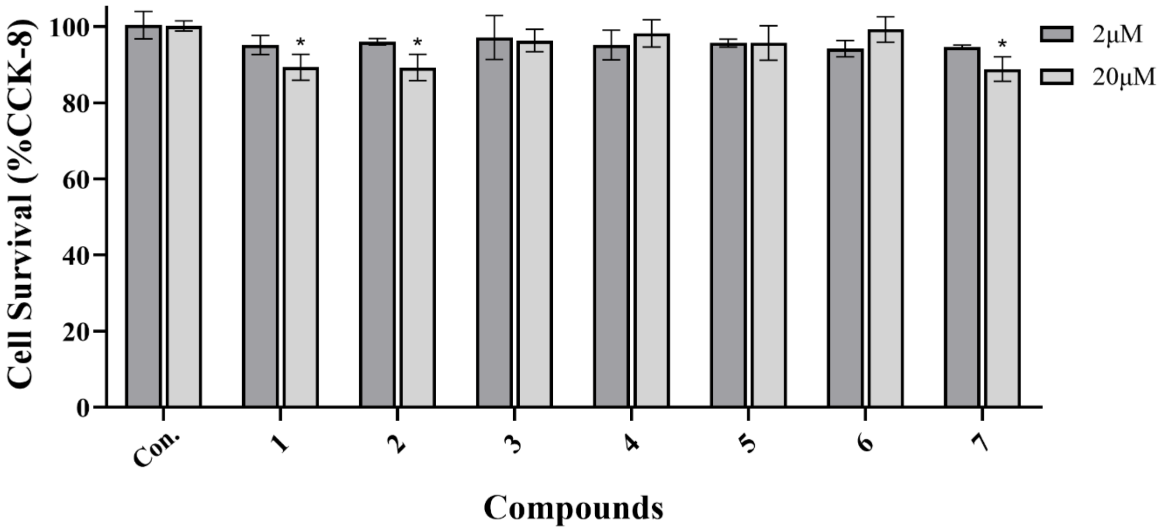

Figure S69. The cytotoxicity of compounds **1**–**7** against A549 cell line under two concentrations. Results are represented as means ± SEM of three independent experiments and were analyzed by Student's *t*-test using Prism 8.0 (GraphPad, La Jolla, CA, USA). Statistical significance was set at * $P < 0.05$.
